# Substrate Specificities of Variants of Barley (1,3)- and (1,3;1,4)-β-d-Glucanases Resulting from Mutagenesis and Segment Hybridization

**DOI:** 10.1021/acs.biochem.3c00673

**Published:** 2024-04-10

**Authors:** Mu-Rong Kao, Jake Parker, Daniel Oehme, Shu-Chieh Chang, Lin-Chen Cheng, Damao Wang, Vaibhav Srivastava, John M. Wagner, Philip J. Harris, Yves S. Y. Hsieh

**Affiliations:** † Division of Glycoscience, Department of Chemistry, School of Engineering Sciences in Chemistry, Biotechnology and Health, Royal Institute of Technology (KTH), AlbaNova University Centre, Stockholm SE-10691, Sweden; ‡ School of Pharmacy, College of Pharmacy, Taipei Medical University, 250 Wuxing Street, Taipei 11031, Taiwan; § School of Agriculture, Food and Wine, University of Adelaide, Waite Campus, Glen Osmond SA 5064, Australia; ∥ IBM Research Collaboratory for Life Sciences, Melbourne, Victoria 3010, Australia; ⊥ College of Food Science, Southwest University, Chongqing 400715, China; # School of Biological Sciences, The University of Auckland, Auckland Mail Centre, Private Bag 92019, Auckland 1142, New Zealand

## Abstract

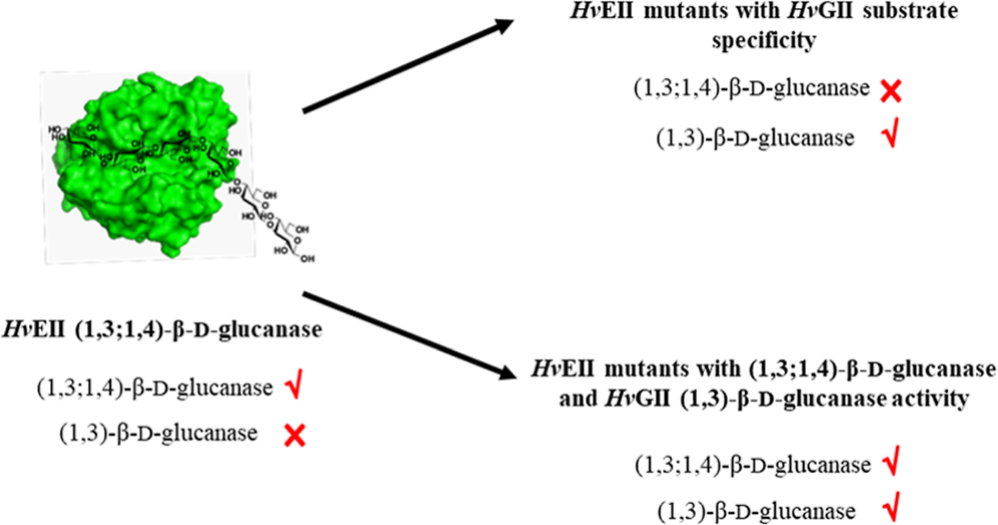

Barley (1,3;1,4)-β-d-glucanase is believed to have evolved from an ancestral monocotyledon (1,3)-β-d-glucanase, enabling the hydrolysis of (1,3;1,4)-β-d-glucans in the cell walls of leaves and germinating grains. In the present study, we investigated the substrate specificities of variants of the barley enzymes (1,3;1,4)-β-d-glucan endohydrolase [(1,3;1,4)-β-d-glucanase] isoenzyme EII (*Hv*EII) and (1,3)-β-d-glucan endohydrolase [(1,3)-β-d-glucanase] isoenzyme GII (*Hv*GII) obtained by protein segment hybridization and site-directed mutagenesis. Using protein segment hybridization, we obtained three variants of *Hv*EII in which the substrate specificity was that of a (1,3)-β-d-glucanase and one variant that hydrolyzed both (1,3)-β-d-glucans and (1,3;1,4)-β-d-glucans; the wild-type enzyme hydrolyzed only (1,3;1,4)-β-d-glucans. Using substitutions of specific amino acid residues, we obtained one variant of *Hv*EII that hydrolyzed both substrates. However, neither protein segment hybridization nor substitutions of specific amino acid residues gave variants of *Hv*GII that could hydrolyze (1,3;1,4)-β-d-glucans; the wild-type enzyme hydrolyzed only (1,3)-β-d-glucans. Other *Hv*EII and *Hv*GII variants showed changes in specific activity and their ability to degrade the (1,3;1,4)-β-d-glucans or (1,3)-β-d-glucans to larger oligosaccharides. We also used molecular dynamics simulations to identify amino-acid residues or structural regions of wild-type *Hv*EII and *Hv*GII that interact with (1,3;1,4)-β-d-glucans and (1,3)-β-d-glucans, respectively, and may be responsible for the substrate specificities of the two enzymes.

## Introduction

**Figure 1:**
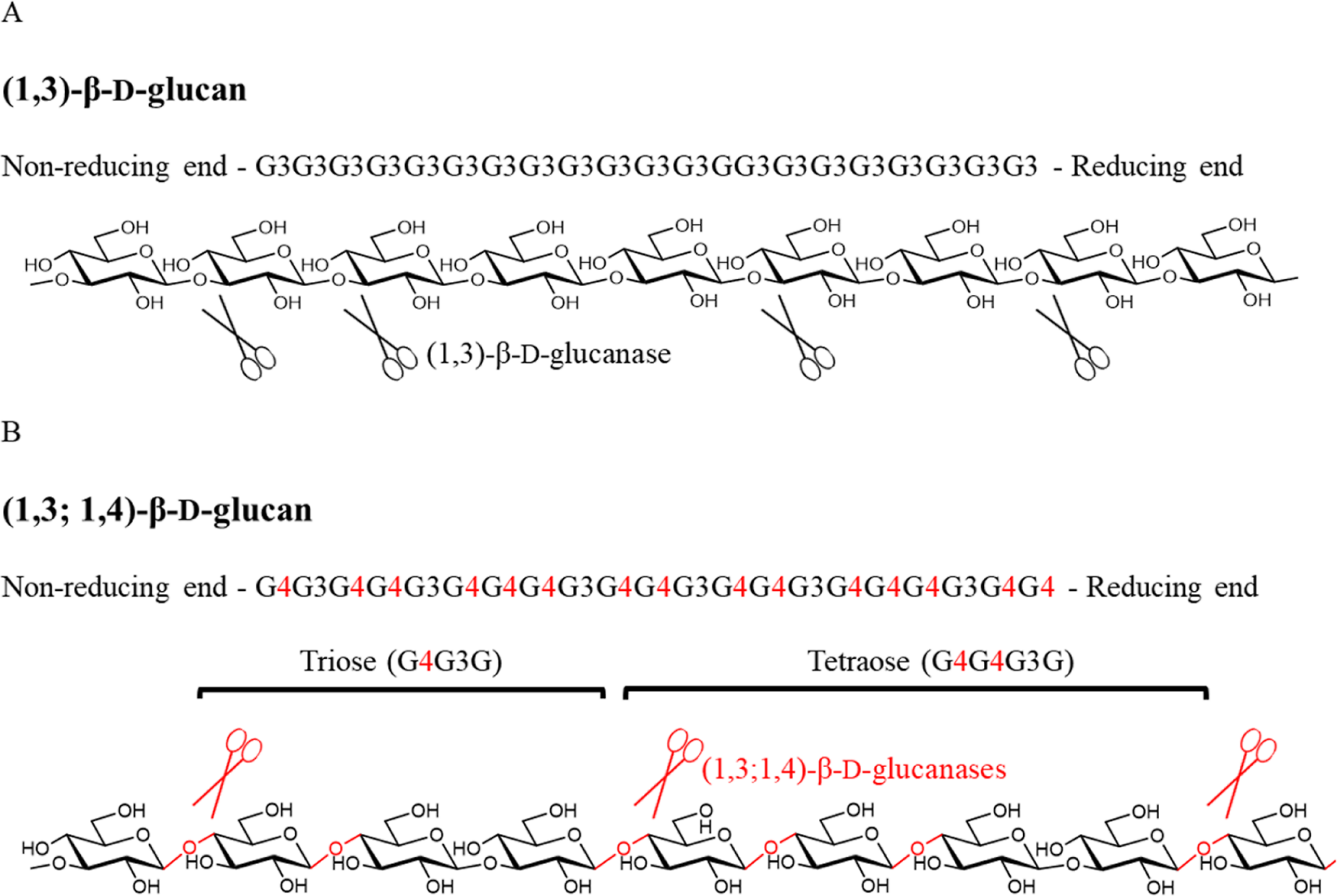
Structures of (1,3)-β-d-glucan (A) and (1,3;1,4)-β-d-glucan (B). β-1,3-Glycosidic bonds are in black, and β-1,4-glyosidic bonds are in red. The cleavage sites for (1,3)- and (1,3;1,4)-β-d-glucanases are represented by black and red scissors, respectively.

Grasses, including barley and other cereals, belong to the highly successful plant family Poaceae that is estimated to cover more than 20% of the Earth’s land surface.^1^ The cell walls of this family, and several related families within the order Poales, can be distinguished from those of other flowering plants (angiosperms) by the presence of (1,3;1,4)-β-d-glucans.^2,3^ Indeed, (1,3;1,4)-β-d-glucans with varying degrees of polymerization (DP) are highly abundant in nature as a consequence of the evolutionary success of the grass family.^3−5^ Given that the Poaceae family represents one of the most recently evolved groups of plants, it can be assumed that the evolution of the genes involved in the biosynthesis of (1,3;1,4)-β-d-glucans was also a relatively recent event.^5−7^ At the same time, for the long-term biological adoption of the newly synthesized (1,3;1,4)-β-d-glucans in the cell walls of grasses, it would have been necessary to evolve new genes that encode glycoside hydrolases (GH) that are specific for (1,3;1,4)-β-d-glucan degradation.^6^ Grass (1,3;1,4)-β-d-glucans are unbranched homoglucans, composed predominantly of two and three consecutive (1,4) linkages [β-Glc*p*-(1,4)-β-Glc*p*-(1,4)-β-Glc*p* (a cellotriosyl unit) and β-Glc*p*-(1,4)-β-Glc*p*-(1,4)-β-Glc*p*-(1,4)-β-Glc*p* (a cellotetraosyl unit), respectively] that are usually found separated by a single (1,3) linkage; two or more adjacent (1,3)-β-glucosyl residues are seldom if ever present (Figure 1). The glycoside hydrolase (GH) (1,3;1,4)-β-d-glucan endohydrolase [(1,3;1,4)-β-d-glucanase] specifically hydrolyses all the (1,4)-linkages in (1,3;1,4)-β-d-glucans that immediately follow (1,3)-linkages (on the reducing side), producing the triose β-Glc*p*-(1,4)-β-Glc*p*-(1,3)-β-Glc*p* (abbreviated to G4G3G, DP3) and the tetraose β-Glc*p*-(1,4)-β-Glc*p*-(1,4)-β-Glc*p*-(1,3)-β-Glc*p* (G4G4G3G, DP4) oligosaccharides that are easily separated and quantified.^6^ It should be noted that blocks of up to 10 adjacent (1,4)-linkages may occur in these polysaccharides and that hydrolysis will also release oligosaccharides with a higher DP.

The action of (1,3;1,4)-β-d-glucanases is critically important for grain germination.^8^ Barley (*Hordeum vulgare*) endosperm cell walls consist of approximately 70% (1,3;1,4)-β-d-glucans by weight.^9^ At a very early stage of germination, the barley (1,3;1,4)-β-d-glucanase *Hv*EI and *Hv*EII isozymes, which are expressed in the scutellar epithelium and aleurone of germinating grains, initiate the breakdown of the cell wall (1,3;1,4)-β-d-glucans, which allows the secretion of α-amylases, peptidases, and other hydrolytic enzymes that result in the mobilization of the endosperm reserves.^10−12^*Hv*EI and *Hv*EII have an absolute specificity for (1,3;1,4)-β-d-glucans.^13^ Because the (1,3;1,4)-β-d-glucans and (1,3;1,4)-β-d-glucanases in angiosperms are apparently confined to the Poaceae and related families, the *Hv*EI and *Hv*EII isozymes likely evolved relatively recently, at approximately 70–55 million years ago, at the divergence of these families from other commelinid monocotyledons.^14^

Petsko et al.^15^ proposed that the generation of new enzymes can occur via two different routes: (1) an ancestral enzyme capable of carrying out the required chemistry undergoes mutational fine-tuning to change its substrate specificity, which results in the appearance of new enzyme species; or (2) a protein without catalytic activity but with a well-defined binding affinity (such as a lectin), may accumulate mutations until a new catalytic ability occurs. Recent studies of several enzyme superfamilies, including enolases, nitroreductases, alkaline phosphatases, amidohydrolase/phosphotriesterases, radical *S*-adenosylmethionine (SAM) enzymes, ATP-grasp enzymes, and cupin enzymes,^16−22^ all support the proposed route 1. These studies have shown that the extant enzymes originated from a common ancestral enzyme via gene duplication and divergent evolution. As these enzymes’ functions diverged, significant changes in amino acid sequences occur, although the metal ion ligand and at least one general acid/base catalyst are always conserved.

It has been postulated that a (1,3)-β-d-glucan endohydrolase [(1,3)-β-d-glucanase] coding gene from monocots was recruited and modified during the evolution of Poaceae, resulting in a (1,3;1,4)-β-d-glucanase with a newly evolved substrate specificity.^6^ This hypothesis is supported by the similarities of the 3D crystal structures of barley (1,3)-β-d-glucanase *Hv*GII (EC 3.2.1.39), which hydrolyses (1,3)-β-d-glucans in angiosperm cell walls and (1,3;1,6)-β-d-glucans in fungal cell walls,^13,23^ and (1,3;1,4)-β-d-glucanase *Hv*EII (EC 3.2.1.73), which has an absolute specificity for (1,3;1,4)-β-d-glucans; both enzymes are in the GH17 family of glycoside hydrolases^24^ The two enzymes share the same (β/α)_8_ barrel (or TIM-barrel) structure with a root-mean-square deviation in C^α^ positions of 0.65 Å for 278 out of 306 amino acid residues.^25^ Because of this, only minor changes of amino acid dispositions at the substrate-binding and catalytic sites may be necessary to convert a (1,3)-β-d-glucanase to a (1,3;1,4)-β-d-glucanase,^26^ and that both barley *Hv*EII and *Hv*GII originated via divergent evolution from a common ancestral (1,3)-β-d-glucanase.^26^ The two barley β-d-glucanases could be considered as a model system to identify the amino acid residue(s) that are responsible for the differences in substrate specificity. In contrast, the (1,3;1,4)-β-d-glucanase from the bacterium *Bacillus subtilis* (GH16 family of glycoside hydrolases) has a “jelly-roll” β-barrel structure^27^ which is very different from the (β/α)_8_ barrel structure of the corresponding barley enzyme (GH17 family). This comparison indicates that the identical substrate specificities of barley and *B. subtilis* (1,3;1,4)-β-d-glucanases have arisen by convergent evolution.^6^

In the present study, we carried out molecular dynamics simulations to identify amino acid residues or structural regions of barley (1,3;1,4)-β-d-glucanase isozyme *Hv*EII and barley (1,3)-β-d-glucanase isozyme *Hv*GII that interact with (1,3;1,4)-β-d-glucans and (1,3)-β-d-glucans, respectively, and could be responsible for the substrate specificities of the two enzymes. We also used site-directed mutagenesis and protein segment hybridization to generate variants of these enzymes, which we tested for alterations in their substrate specificities.

## Materials and Methods

### Materials

Laboratory chemicals were of reagent grade and purchased from Sigma-Aldrich (St. Louis, MO, USA). Barley (1,3;1,4)-β-d-glucan and the oligosaccharides DP3 (G4G3G) and DP4 (G4G4G3G) derived from this glucan, as well as the oligosaccharides laminaribiose, laminaritriose, laminaritetraose, laminaripentaose, and laminarihexaose derived from Curdlan [a (1,3)-β-d-glucan] were obtained from Megazyme (Bray, Ireland). Laminarin from Laminaria digitata (the preferred substrate of *Hv*GII^13,23^) was used as the (1,3)-β-d-glucan substrate and was obtained from Sigma-Aldrich. Oligonucleotide primers were obtained from Geneworks (Thebarton, SA, Australia) and the site-directed mutagenesis kit was obtained from Stratagene (La Jolla, CA, USA). The competent *Escherichia coli* cells were obtained from Life Technologies (Carlsbad, CA, USA). The synthetic protein segment changed *Hv*EII and *Hv*GII mutants were purchased from GenScript (Piscataway, NJ, USA).

### Phylogeny Study of *Hv*EII and *Hv*GII

Sequences were aligned using Clustal Omega^28^ and the phylogenetic tree was built using the nearest neighbor-joining method with the Molecular Evolutionary Genetics Analysis (MEGA) software.^29,30^

### Protein and β-d-Glucan Structures

Crystal structures of *endo* (1,3)- and (1,3;1,4)-β-d-glucanase isoenzymes *Hv*GII (PDB ID: 1GHS) and *Hv*EII (PDB ID: 1GHR) from barley were obtained from the Protein Data Bank.^25^ Visualization of the 3D protein structure was performed with the PyMol Molecular Graphics System v2.2.3 (Schrodinger, LLC). All crystallized water molecules were discarded and the protonation states of histidine were visually inspected in Visual Molecular Dynamics (VMD)^31^ to ensure optimal hydrogen bonding. Models of (1,3)- and (1,3;1,4)-β-d-glucan oligosaccharides with a degree of polymerization of 14 (DP14) were constructed with an in-house version of CarbBuilder.^32^ The (1,3;1,4)-β-d-glucan oligosaccharide was built such that (1,3) linkages were separated by three (1,4)-linkages, and the reducing and nonreducing ends were padded with two (1,4)-linkages. All models were solvated in a box of transferable intermolecular potential 3P (TIP3P) water with at least 12 Å padding in the *x*, *y*, and *z* directions. Sodium and chloride ions were added to neutralize the system and ensure a salt concentration of 0.15 M.

### Substrate Docking

Molecular dynamics-assisted docking by manually positioned centroids of glucan clusters [i.e., DP14 (1,3)- and (1,3;1,4)-β-d-glucan oligosaccharides] were positioned above the substrate clefts of the two enzymes, and their reducing ends lay at the western end of the protein as predicted by Hrmova et al.,^33^ with restraints on glucosyl residues surrounding glycosidic linkage to be cleaved and bringing these residues into close proximity to the known catalytic residues. The resulting trajectories were visualized by using VMD and analyzed by using a combination of VMD tools and in-house scripts.

### Molecular Dynamics Simulation

All simulations were performed in duplicate using NAMD 2.9^34^ at 310 K. The CHARMM 36 carbohydrate force field^35−38^ was used for carbohydrate parameters while CHAMM 27 parameters with CMAP corrections were used for protein parameters. Periodic boundary conditions were applied in all three directions. An initial simulation stage of 5000 steps of minimization followed by 250,000 steps of constant pressure–temperature Langevin dynamics was performed to optimize the position of the added waters and ions. A time step of 2 fs and a nonbonded cutoff of 10 Å were utilized, with the RATTLE/SETTLE algorithms^39,40^ used to constrain the bond lengths from hydrogen to heavy atoms. The pressure was kept constant using a Langevin piston barostat, and the particle mesh Ewald method was used to calculate long-range electrostatic interactions.^41^ Phase simulations were performed in a constant pressure–temperature ensemble.

### Relaxed Substrate Conformations

Low energy conformations of linear forms of the β-glucan polysaccharides that could more effectively bind in the enzymatic cleft were produced using steered molecular dynamics (SMD). A two-step simulation protocol was followed in NAMD whereby the β-glucan molecule was first stretched at a constant velocity of 200 Å/ns with a spring constant of 200 kcal/mol/Å^2^ for 30 ps. The β-d-glucan was allowed to relax for 2 ns. Ten conformations were taken from 5 replicate simulations and clustered using the VMD clustering plugin. The centroid of the cluster that was most heavily populated was subsequently used as a representative structure for all future simulations.

### Synthesis of *HvEII* and *HvGII* Variants

Targeted mutations were introduced in *HvEII and HvGII* cDNAs using the QuikChange II Site-Directed Mutagenesis Kit (Stratagene, La Jolla, CA, USA). The mutagenesis reactions were carried out following the manufacturer’s instructions. Plasmids carrying putative mutations were sequenced to confirm the presence of the targeted changes at the Australian Genome Research Facility (AGRF, Adelaide, SA).

### Heterologous Expression of *Hv*EII and *Hv*GII Variants

Full-length cDNAs were available for both the (1,3)-β-d-glucanase *Hv*GII and the (1,3;1,4)-β-d-glucanase *Hv*EII, with the cDNA inserted in the *NdeI*/*BamHI* restriction site of a pET3a vector. The final constructs were transformed into *E. coli* BL21 cells (Invitrogen, Waltham, MA, USA) by heat shock treatment and were grown in LB broth and 50 μg/mL ampicillin at 37 °C until the absorbance at 600 nm was 0.5–0.6, after which IPTG was added to a final concentration of 1 mM. The cells were grown for 16 h at 23 °C before harvesting by centrifugation at 6000*g*. The cell pellet was resuspended in Xtractor (Clontech, Mountain View, CA, USA) at 4 °C for 1 h. The soluble fraction was applied to a TALON metal affinity resin (Clontech), and the target protein was eluted with 200 mM imidazole.^42^ The eluted material was desalted by buffer exchange using an Amicon centrifuge cartridge, and the protein concentration was measured using the Bradford assay with bovine serum albumin (BSA) as the reference protein.^43^ The purity of purified recombinant enzyme preparations (4 μg) was evaluated by separating the component proteins with SDS-PAGE and using the image processing software ImageJ.^44^ The specific activity was determined using purified enzyme preparation (4 μg) in 50 mM NaOAc buffer (pH 5) containing 0.25% of β-d-glucans, and the reaction was performed at 37 °C for 30 min. The reducing sugar concentration in the hydrolysis products was determined using the dinitrosalicylic acid (DNS) method^45^ with the standard curve obtained using glucose that had been serially diluted.

### Substrate Specificity Study

The substrate specificity of *Hv*EII and *Hv*GII variants was examined with two polysaccharides, (1,3)-β-d-glucan (laminarin; 1 mg/mL) and (1,3;1,4)-β-d-glucan (1 mg/mL), and the DP6 oligosaccharide laminarihexaose (1 mg/mL). The substrates and enzymes (25 nmol) were incubated for 16 h in 200 μL of 50 mM NaOAc buffer (pH 5), containing 0.2 mg/mL BSA, and the enzyme reactions were stopped by the addition of 4 volumes of ethanol. The hydrolysates were centrifuged at 13,000*g*, and the supernatants were diluted using Milli-Q water before separation by high-performance anion-exchange chromatography (HPAEC) on a Dionex BioLC system fitted with a pulsed amperometric detector (PAD) (Dionex ICS 5000 Dionex, Sunnyvale, CA, USA). Separation of oligosaccharides was performed on a Dionex CarboPac PA200 column (5.5 μm, 150 × 3 mm) at a flow rate of 0.5 mL min^–1^ using 100 mM NaOH (eluent A) and 100 mM NaOH + 1 M NaOAc (eluent B) with a linear gradient of 1–13% B over 15 min. The oligosaccharides laminaribiose, laminaritriose, laminaritetraose, laminaripentaose, and laminarihexaose were used as reference (1,3)-β-d-gluco-oligosaccharides. DP3 (G4G3G) and DP4 (G4G4G3G), derived from barley (1,3;1,4)-β-d-glucans, were also used as reference oligosaccharides. For mass spectrometry of the oligosaccharide products, enzyme hydrolysates were mixed with 10 mM 2,5-dihydroxybenzoic acid (DHB) and 10 mM NaCl in the ratio of 5:5:3, and dried on the metal plate of an Ultraflex II matrix-assisted laser desorption ionization time-of-flight/time-of-flight (MALDI-TOF/TOF) mass spectrometer (Bruker Daltonik GmbH, Bremen, Germany), before being analyzed in the positive-ion reflectron mode with mass spectra obtained over the mass to charge ratio (*m*/*z*) range of 400–1800.

## Results and Discussion

### Sequence Comparison of β-Glucanases within the Grass Family

To understand the evolutionary relationships among β-d-glucanases of different angiosperm species, we conducted a phylogenetic. The phylogenetic tree of angiosperm (1,3)-β-d-glucanases has two distinct clades, one containing the (1,3)-β-d-glucanases of the grass family Poaceae in the monocotyledons and the other containing the (1,3)-β-d-glucanases of the eudicotyledons (Figure S1). Barley (1,3)-β-d-glucanase isoenzymes *Hv*GI to *Hv*GV and *Hv*GVII are more closely related to barley (1,3;1,4)-β-d-glucanase isoenzymes *Hv*EI and *Hv*EII^6^ than to (1,3)-β-d-glucanases from eudicotyledons or from fungi (used as the outgroup) (Figures S1 and S2). This finding supports the hypothesis that these two barley β-d-glucanases evolved from an ancestral monocotyledon (1,3)-β-d-glucanase that underwent divergent evolution to become highly specialized.

**Figure 2:**
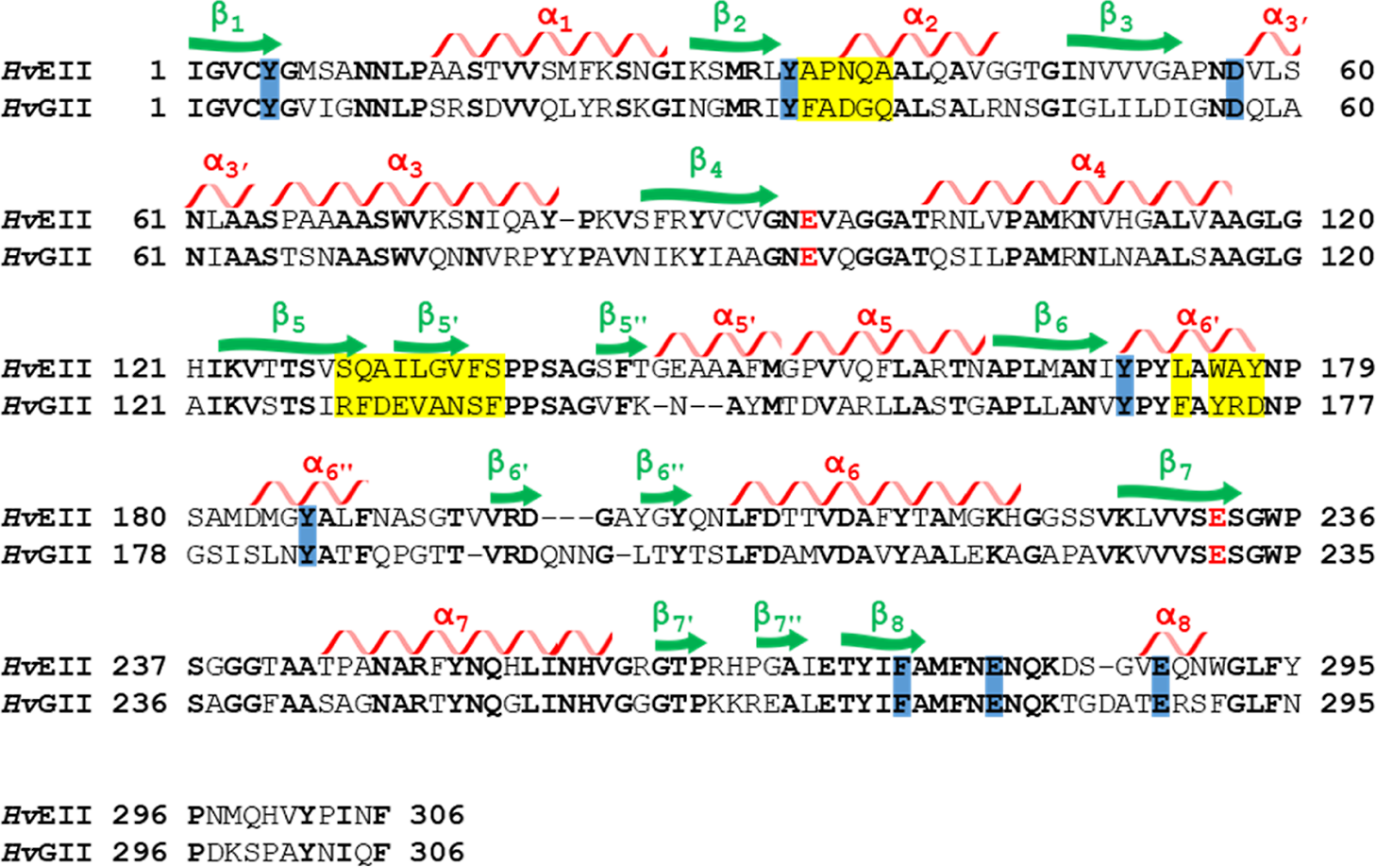
Sequence alignment of barley (1,3;1,4)-β-d-glucanase *Hv*EII and (1,3)-β-d-glucanase *Hv*GII. Conserved sequences and catalytic residues are colored black and red, respectively. Conserved hydrophobic and negatively charged amino acid residues surrounding the substrate cleft are highlighted in blue. Amino acid residues modified by site-directed mutagenesis in *Hv*EII or *Hv*GII in this study are highlighted in yellow. The α-helices and β-strands in the (α/β)_8_ barrel structure of *Hv*EII are indicated by red spirals and green arrows, respectively. Minor structures are indicated by the prime symbol ′ or the double prime symbol ″ after the helix and strand number.

The *endo*-(1,3)-β-d-glucanases are widespread in angiosperms (Figures S1 and S2), with crucial roles in the mobilization of callose [a (1,3-β-d-glucan)] during growth and development and in defense against fungal pathogens, with the enzymes also able to hydrolyze (1,3;1,6)-β-d-glucans in fungal cell walls. *Endo*-(1,3;1,4)-β-d-glucanases are mainly found in the grass family Poaceae (Figure S2), which coincides with the occurrence of (1,3;1,4)-β-d-glucans in their cell walls. Alignment of the amino acid sequences of the two β-d-glucanases *Hv*GII and *Hv*EII showed a sequence identity of 52%. The catalytic glutamic acids are conserved at Glu^94^/Glu^231^ of *Hv*GIIs and Glu^93^/Glu^232^ of *Hv*EII (Figure 2). In addition, within the Poaceae family, the catalytic nucleophile and proton donors were conserved among homologues of the enzymes EII and GII. Sequence alignment also showed that negatively charged amino acid residues (Asp^57^, Glu^280^, Glu^288^) and aromatic amino acid residues (Tyr^5^, Tyr^33^, Tyr^170^, Tyr^186^, Phe^275^) were highly conserved in the substrate-binding cleft of both EII and GII homologues (Figure S3). We have also identified four conserved aromatic amino acid residues in the substrate-binding cleft of *Hv*GII and its homologues, at positions Phe^34^, Phe^137^, Phe^171^, and Phe^291^, whereas Phe^135^, Tyr^177^, and Trp^291^ were conserved in *Hv*EII and its homologues. These aromatic residues are likely to be glucosyl-binding subsites along the polysaccharide-binding cleft.^33^ As such, the differences within the conserved aromatic residues at the substrate-binding cleft of the two isozyme clades could play a crucial role in the regulation of the difference in substrate specificity between *Hv*GII and *Hv*EII.

### Molecular Dynamics Simulation of β-d-Glucan Binding

In addition to sequence alignment studies, we have carried out simulations of docking of DP14 (1,3)- and (1,3;1,4)-β-d-glucan oligosaccharides into the deep substrate-binding groove of barley *Hv*EII and *Hv*GII, using the molecular dynamics simulation programs NAMD and AMBER. The goal was to pinpoint the key residues in *Hv*EII and *Hv*GII that interact with the β-d-glucans and that could be responsible for the substrate specificities of the two enzymes. The two enzymes have very similar structures (RMSD 0.65 Å over 280 amino acid residues).^25^ Molecular dynamics (MD)-assisted dockings were performed by manually positioned centroids of glucan clusters on both enzymes (Figure S4). The (1,3)- and (1,3;1,4)-β-d-glucan oligosaccharides were positioned in the substrate clefts of the two enzymes, with restraints on the glucosyl residues surrounding the glycosidic linkages to be hydrolyzed, bringing these residues in close proximity to known catalytic residues (Figure S4). The nonreducing and reducing ends of the two β-d-glucan oligosaccharides were positioned at the left and right sides of the substrate cleft, respectively (Figure S4).

Supported by the highest net increase in the entropy of the system, we identified seven amino acid residues, ranked from the most to least favorable interaction between *Hv*EII and the substrate in the following order: Tyr^177^, Ala^34^, Pro^35^, Val^134^, Phe^135^, Ser^128^, and Gln^129^. These amino acid residues span the edge of the substrate binding pocket and involve direct contact with the (1,3;1,4)-β-d-glucan oligosaccharide. The Tyr^177^ is highly conserved among (1,3;1,4)-β-d-glucanases in the Poaceae, and this residue gave the highest net increase of entropy. The aromatic side chains in the (1,3;1,4)-β-d-glucanases (Tyr^177^ and Phe^135^) could interact directly with the glucosyl moiety in a stacking geometry via CH/π interactions, which is not possible in *Hv*GII and other Poaceae (1,3)-β-d-glucanases, which have a glycine (Gly) or an aspartic acid (Asp) at the corresponding position (Figures 2 and S3). Indeed, BLAST analysis showed that this consensus site is conserved, with aromatic amino acid residues such as Tyr, Phe, and Trp found in over 100 homologous Poaceae (1,3;1,4)-β-d-glucanases. In contrast, *Hv*GII and other Poaceae homologues possess amino acid residues such as Asp and Gly that compared with Tyr have smaller, nonhydrophobic side chains. This suggests that the steric effect of Tyr^177^ in *Hv*EII could play a key role in substrate specificity.

**Figure 3:**
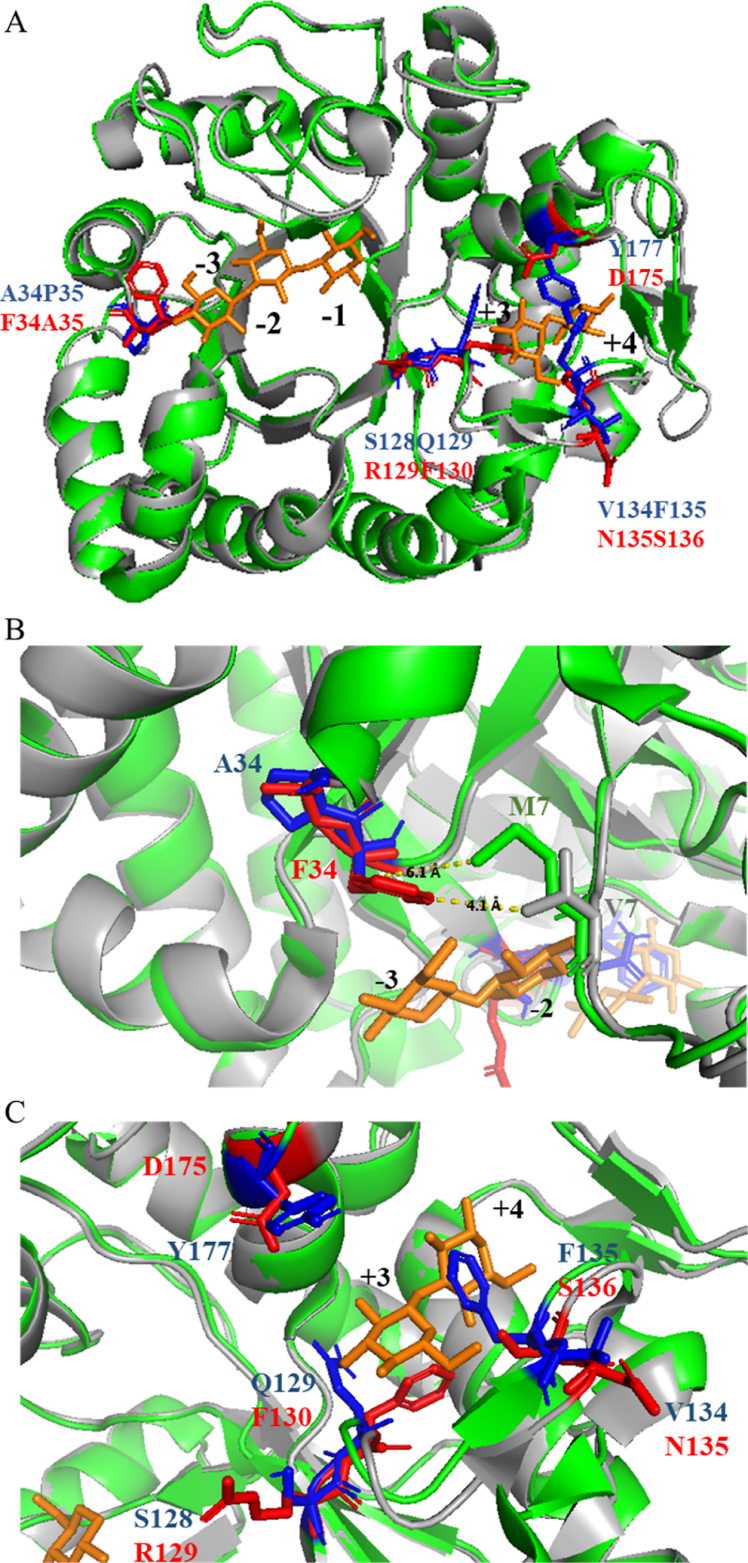
Molecular dynamics simulation identified specific amino acid residues of *Hv*EII and *Hv*GII that are likely to be involved in substrate binding. Structure superposition of (1,3;1,4)-β-d-glucanase *Hv*EII and (1,3)-β-d-glucanase *Hv*GII (A). Residue Ala^34^ of *Hv*EII is close to the corresponding substrate binding subsite −3 of potato (1,3)-β-d-glucanase (B). Residues Ser^128^, Gln^129^, Val^134^, Phe^135^, and Typ^177^ are located near the corresponding substrate binding subsites +3 and +4 of potato (1,3)-β-d-glucanase (C), these residues may play a role in stabilizing the substrate as it binds to the enzyme. The amino acid residues are represented by blue sticks on *Hv*EII, whereas their homologues are represented by red sticks on *Hv*GII. 3D Protein structures of *Hv*EII (PDB ID: 1GHR) and *Hv*GII (PDB ID: 1GHS) and the glucose units from potato (1,3)-β-d-glucanase GCLU20–2 (PDB ID: 4GZJ) are represented in green, in gray and in orange, respectively.

For better visualization of different amino acid residues determined by the molecular dynamics simulation, positions of substrate binding subsites in (1,3)-β-d-glucanase were determined by superimposing *Hv*GII with the eudicotyledon potato (*Solanum tuberosum*) (1,3)-β-d-glucanase GLUB20–2 (PDB ID: 4GZJ) (Figures 3A and S5).^46^ The Ala^34^ and Pro^35^ residues of *Hv*EII located close to the substrate binding subsite −3 of the (1,3)-β-d-glucanase are highly conserved among (1,3;1,4)-β-d-glucanases (Figure 3B). Additionally, the hydrophobic residue Met^7^, positioned on the nearby loop, is highly conserved. In *Hv*EII, the distance between Met^7^ and Ala^34^ is 6.1 Å, indicating that the proximity of the respective loops is unlikely to be stabilized by hydrophobic interactions. In contrast, in *Hv*GII, the distance between Val^7^ and Phe^34^ has been shortened to 4.1 Å, which suggests that there is a steric hindrance that may restrict access of (1,3;1,4)-β-d-glucan to the active site of the enzyme. The high ranking of Phe^135^ and Gln^129^ in the MD study could be associated with the orientation of the two amino acid side chain moieties, which protrude from the substrate-binding cleft and sterically hinder the binding of (1,3;1,4)-β-d-glucans (Figure 3C). The benzyl side chain of Phe^135^ in *Hv*EII was almost in contact with the glucopyranosyl unit at subsite +3; the substitution of Phe^135^ could also affect the binding of (1,3;1,4)-β-d-glucans.

### *Hv*EII and *Hv*GII Variants Generated via Site-Directed Mutagenesis

The current underlying assumption is that (1,3;1,4)-β-d-glucanase *Hv*EII evolved from an ancestral (1,3)-β-d-glucanase by changes in the dispositions of amino acid residues at the substrate-binding and catalytic sites. To confirm the in silico prediction of amino acid residues that could control substrate specificity, we generated enzyme variants bearing multiple mutations by site-directed mutagenesis (Figure S6 and Table 1). Variant enzymes were assayed against both (1,3)-β-d-glucans and (1,3;1,4)-β-d-glucans, and the oligosaccharides released were separated and qualitatively characterized by using high-performance anion-exchange chromatography with pulsed-amperometric detection (HPAEC-PAD) against known standards.

**Table 1:** Positions of Amino Acid Substituted in *Hv*EII and *Hv*GII Variants and Their Substrate Specificities

	substrate specificity		(1,3;1,4)-β-d-glucan oligosaccharides produced	
*Hv*EII wild-type and variants^a^	(1,3;1,4)-β-d-glucan	(1,3)-β-d-glucan	DP3:DP4 ratio	DP3 + DP4/total products (%)
*Hv*EII wild-type	+	–	2.05:1	89.3
*Hv*EII E93A	–	–	n.d.	n.d.
V1: A34F, P35A	+	–	2.11:1	91.9
V2: A34F, P35A, N36D, Q37G, A38Q	+	–	0.82:1	40.5
V3: Y177D	+	–	2.06:1	89.9
V4: Y177G	+	–	2.11:1	92.2
V5: L173F, W175Y, A176R	+	–	2.39:1	60
V6: S128R, Q129F	+	+	2.15:1	90.7
V7: L132V, G133A, V134N, F135S, S136F	+	–	1.33:1	59.6
V8: V134N, F135S, S136F	+	–	2.05:1	89.4
V9: A34F, P35A, Y177D	+	–	1.33:1	56.1
V10: A34F, P35A, S128R, Q129F, Y177D	–	–	n.d.	n.d.
V11: A34F, P35A, N36D, Q37G, A38Q, S128R, Q129F, Y177D	–	–	n.d.	n.d.

	substrate specificity		
*Hv*GII wild-type and variants^b^	(1,3;1,4)-β-d-glucan	(1,3)-β-d-glucan	(1,3)-β-d-glucan oligosaccharides produced
*Hv*GII wild-type	–	+	Hex_2_
*Hv*GII E94A	–	+	n.d.
W1: F34A	–	+	Hex_2_, Hex_3_
W2: A35P	–	+	Hex_2_, Hex_3_, Hex_5_
W3: F34A, A35P	–	+	Hex_2_, Hex_3_
W4: F34A, A35P, D36N, G37Q, Q38A	–	+	Hex_2_, Hex_3_, Hex_4_, Hex_5_
W5: D175Y	–	+	Hex_2_, Hex_3_, Hex_4_, Hex_5_, Hex_6_
W6: F171L, Y173W, R174A, D175Y	–	+	Hex_2_, Hex_3_, Hex_4_, Hex_5_, Hex_6_
W7: R129S, F130Q	–	+	Hex_2_
W8: D131A, E132I, V133L, A134G	–	+	Hex_2_, Hex_3_, Hex_4_, Hex_5_, Hex_6_
W9: S136F, F137S	–	+	Hex_2_, Hex_3_, Hex_4_

a Figures S7 and S8 contain information about *Hv*EII mutant V variants, including the protein sequences and oligosaccharide profiles.

b Figures S9 and S10 contain information about *Hv*GII mutant W variants, including the protein sequences and oligosaccharide profiles n.d.: not detected.

The results indicated that the recombinant wild-type *Hv*EII (WT *Hv*EII) hydrolyzed only the (1,3;1,4)-β-d-glucan with a specific activity of 10.2 ± 0.4 U/mg (Figures S7A and S8A,B). The products of enzymatic hydrolysis were mostly DP3 and DP4 oligosaccharides (about 89.3%) with a DP3:DP4 ratio of 2.05:1. The wild-type *Hv*GII (WT *Hv*GII), on the other hand, hydrolyzed only the (1,3)-β-d-glucans, with a specific activity of 10.9 ± 1.2 U/mg (Figures S9A and S10A). We also substituted the conserved catalytic glutamic acid (i.e., Glu^93^ in *Hv*EII and Glu^94^ in *Hv*GII) with an alanine (Ala) (variants E93A and E94A, respectively) and found no reaction products (Figures S7B, S8C, S9B, and S10B), indicating that this glutamic acid residue is essential for the enzymatic activity of both *Hv*EII and *Hv*GII.

To determine if the substrate specificity of *Hv*EII can be converted from that of a (1,3;1,4)-β-d-glucanase to a (1,3)-β-d-glucanase, we performed site-directed mutagenesis on *Hv*EII to substitute specific amino acid residues with those found in *Hv*GII. Seven amino acids, predicted by MD simulation to be important in substrate binding and specificity in *Hv*EII, are positioned distinctly across the substrate binding site (Figure 3A). These residues are Ala^34^, Pro^35^, Ser^128^, Gln^129^, Val^134^, Phe^135^, and Tyr^177^. Subsequently, substitutions were made at positions around Ala^34^/Pro^35^, Ser^128^/Gln^129^, Val^134^/Phe^135^, and Tyr^177^ (Table 1). Variants V1 and V2 carried mutations around Ala^34^/Pro^35^, which were conserved and located near binding subsite −3 (Figure 3B). Variants V3 to V5, V6 and V7 to V8 had mutations around Tyr^177^, Ser^128^/Gln^129^ and Val^134^/Phe^135^, respectively (Figure 3C). Finally, variants V9–V11 had multiple substitutions around all three positions.

Variants V1 to V5 indicated that modifications around Ala^34^ or Tyr^177^ did not affect *Hv*EII’s substrate specificity (Figures S7C–G and S8D–H). The *Hv*EII variant V9, carrying a triple mutation (A34F + P35A + Y177D), also maintained its (1,3;1,4)-β-d-glucanase activity, but multiple substitutions of amino acid residues surrounding Ala^34^, Ser^128^, and Tyr^177^ in variants V10 and V11 completely abolished hydrolytic activity (Figures S7K–M and S8L–N).

**Figure 4:**
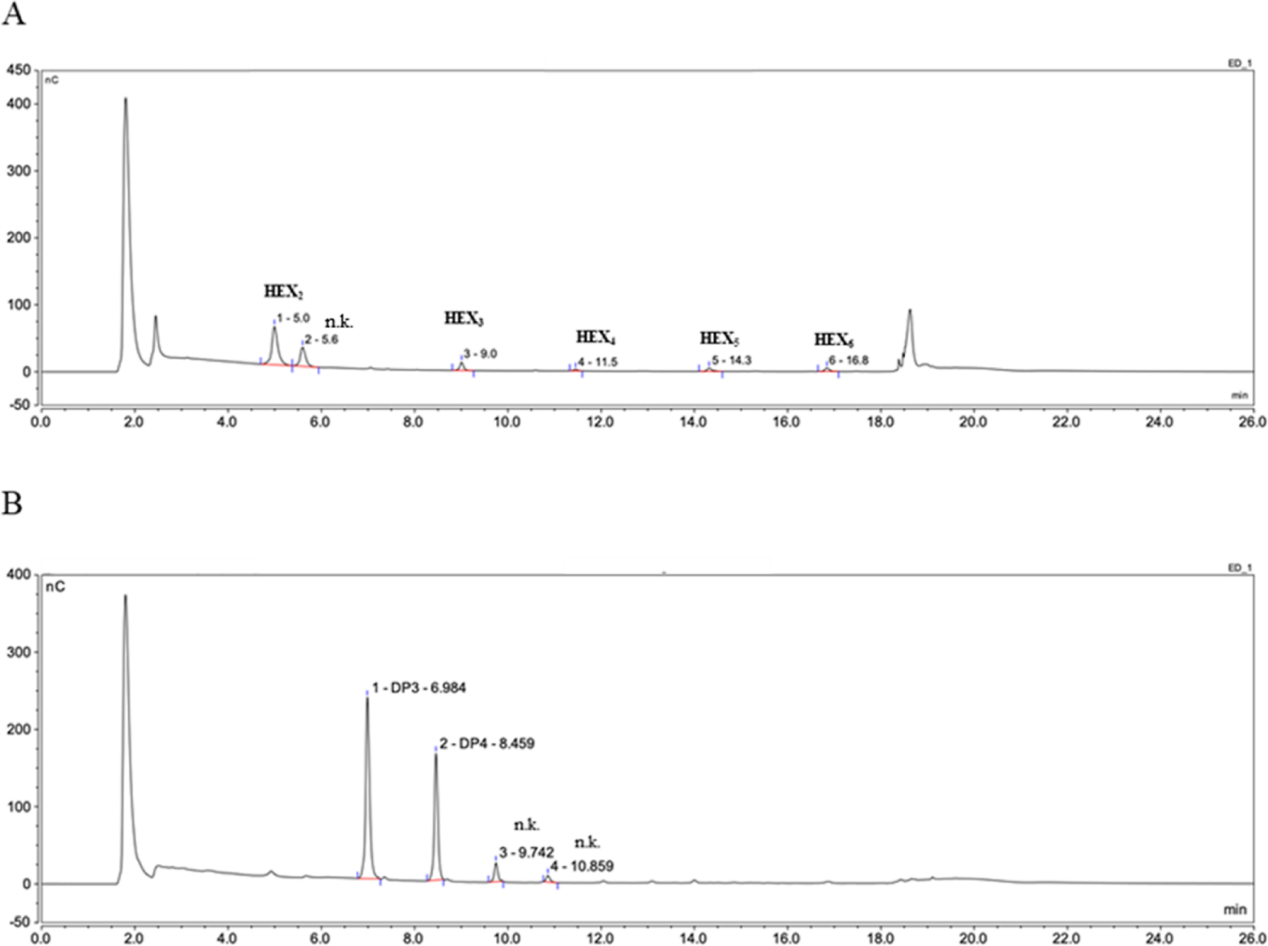
*Hv*EII variant V6 (S128R + Q129F) hydrolyzes both (1,3)- and (1,3;1,4)-β-d-glucans. After digestion of (1,3)-β-d-glucan (A) and (1,3;1,4)-β-d-glucan (B) by the *Hv*EII variant V6, hydrolysis products were detected by HPAEC-PAD. The peaks were assigned using the reference standards G4G3G (DP3), G4G4G3G (DP4), laminaribiose (Hex_2_), laminaritriose (Hex_3_), laminaritetraose (Hex_4_), laminaripentaose (Hex_5_), and laminarihexaose (Hex_6_). The (1,3)-β-d-glucanase activity of the V6 variant was confirmed using MALDI-TOF MS (Supporting Information Figure S13). n.k. = not known.

The *Hv*EII variant V6 (S128R + Q129F) showed hydrolytic activity against (1,3)-β-d-glucans (Figures 4A, S7H, and S8I), but because of its low activity, its specific activity against (1,3)-β-d-glucans could not be measured by the DNS colorimetric assay. The V6 variant also hydrolyzed the (1,3)-β-d-gluco-oligosaccharide laminarihexaose. However, the V6 variant maintained its (1,3;1,4)-β-d-glucanase activity, producing the DP3 and DP4 oligosaccharides, with a specific activity of 9.9 ± 0.3 U/mg, similar to that of WT *Hv*EII (10.2 ± 0.4 U/mg) (Figures 4B, S7A, and S7H). Although the substrate specificity of the *Hv*EII variant V6 was not completely switched to the substrate specificity of *Hv*GII, the V6 variant showed the ability to accommodate both (1,3)-β-d-glucans and (1,3;1,4)-β-d-glucans in the substrate binding groove.

We also found that some of the *Hv*EII variants had substrate affinities toward (1,3;1,4)-β-d-glucans that were modified (Table 1). With WT *Hv*EII, 89.3% of the hydrolysis products were the DP3 and DP4 oligosaccharides with a DP3:DP4 ratio of 2.05:1. Incomplete enzymatic hydrolysis led to an increase in oligosaccharides with a DP > 4. For example, the *Hv*EII variant V9 (A34F + P35A + Y177D) gave a DP3 + DP4/total product percentage of 56.1%, with a DP3:DP4 ratio of 1.33:1 (Table 1). Decreases in these two values could signify incomplete hydrolysis of the substrate, with variant V9 giving a high abundance of oligosaccharides at retention times of 13.14 and 14.11 min, corresponding to putative DP6 and DP7 oligosaccharides, respectively (Figure S8L). Both oligosaccharides were purified on a carbon-cartridge column, eluted with 40% acetonitrile, and used as substrates in a second round of enzyme hydrolysis. We found that WT *Hv*EII was able to hydrolyze the putative DP6 to the DP3 oligosaccharide, and the putative DP7 to the DP3 and DP4 oligosaccharides (Figure S8L). However, *Hv*EII variant V9 was not able to further hydrolyze the DP6 and DP7 products. This indicates that the V9 variant can be used to generate the DP6 and DP7 oligosaccharides as standards. With the *Hv*EII variants V1, V3, V5, V6, and V8 (Figures S7J and S8K), the ratios of DP3:DP4 oligosaccharides produced were in the range 2.05 to 2.39:1 (Table 1), but with the *Hv*EII variants V2, V7 (Figures S7I and S8J) and V9, the ratios were in the range of 0.82–1.33:1, with significant changes in substrate affinity. These results suggested that mutations at positions surrounding Ala^34^, Ser^128^, and Tyr^177^ slow or otherwise impair the hydrolysis of (1,3;1,4)-β-d-glucans.

*Hv*GII variants were generated to investigate if substrate specificity can be converted from that of a (1,3)-β-d-glucanase to a (1,3;1,4)-β-d-glucanase (Table 1). Nine variants (W1–W9) carrying mutations around positions Phe^34^/Ala^35^, Arg^129^/Phe^130^, Asn^135^/Ser^136^, and Asp^175^ and/or their neighboring residues were generated, but all these variants showed only (1,3)-β-d-glucanase activity (Figures S9 and S10), indicating that the substrate specificity had not been changed or loosened by substituting these amino acid residues. However, the catalytic efficiency was modified in the *Hv*GII variants W5, W8, and W9. For example, when the variant W5 hydrolyzed (1,3)-β-d-glucan, it generated an oligosaccharide profile with a higher proportion of the Hex_4_, Hex_5_, and Hex_6_ (1,3)-β-d-gluco-oligosaccharides than that with WT *Hv*GII (Figure S11A,G), indicating that substrate affinity was modified by the single mutation D175Y.

The W8 and W9 variants of *Hv*GII had mutations close to the substrate-binding subsites +3 and +4, specifically near Ser^136^ (Figure 3C), which resulted in decreased specific activities compared with the wild-type enzyme. The specific activities of W8 and W9 were 1.0 ± 0.1 and 7.5 ± 0.2 U/mg, respectively (Figure S9J,K). The other variants of *Hv*GII had specific activities ranging from 9.2 to 11.6 U/mg, similar to that of the wild-type enzyme (10.9 ± 1.2 U/mg).

Additionally, the mutation in the *Hv*EII V8 variant, which was located near Phe^135^ close to subsites +3 and +4, also negatively affected the catalytic activity, with a specific activity of 6.7 ± 1.3 U/mg compared with 10.2 ± 0.4 U/mg for the specific activity of the wild-type enzyme (Figure S7A,J). As these substitutions are close to conserved hydrophobic residues near subsites +3 and +4 in *Hv*GII and *Hv*EII, we speculate that the substrate binding affinity was affected negatively in these mutants, thereby decreasing their catalytic activities.

### *Hv*EII and *Hv*GII Variants Generated via Segment Hybridization

**Figure 5:**
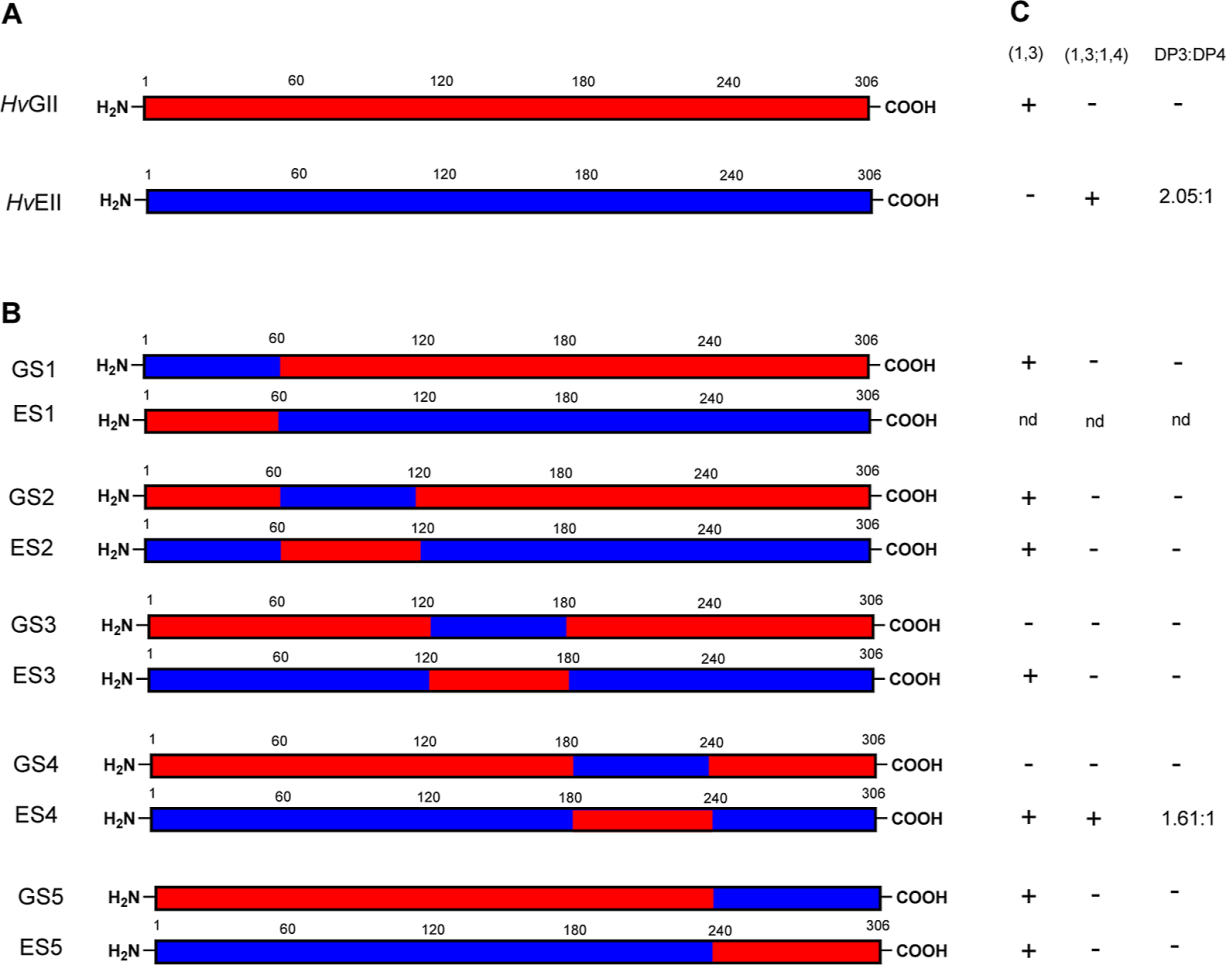
*Hv*EII and *Hv*GII variants derived from segment hybridization. (A) Schematics show the protein sequences of *Hv*GII (red) and *Hv*EII (blue). (B) Chimeric proteins with segments reciprocally swapped between *Hv*GII (red) and *Hv*EII (blue). (C) Substrate specificities toward (1,3) [= (1,3)-β-d-glucan], and (1,3;1,4) [= (1,3;1,4)-β-d-glucan]. n.d. = not determined.

**Figure 6:**
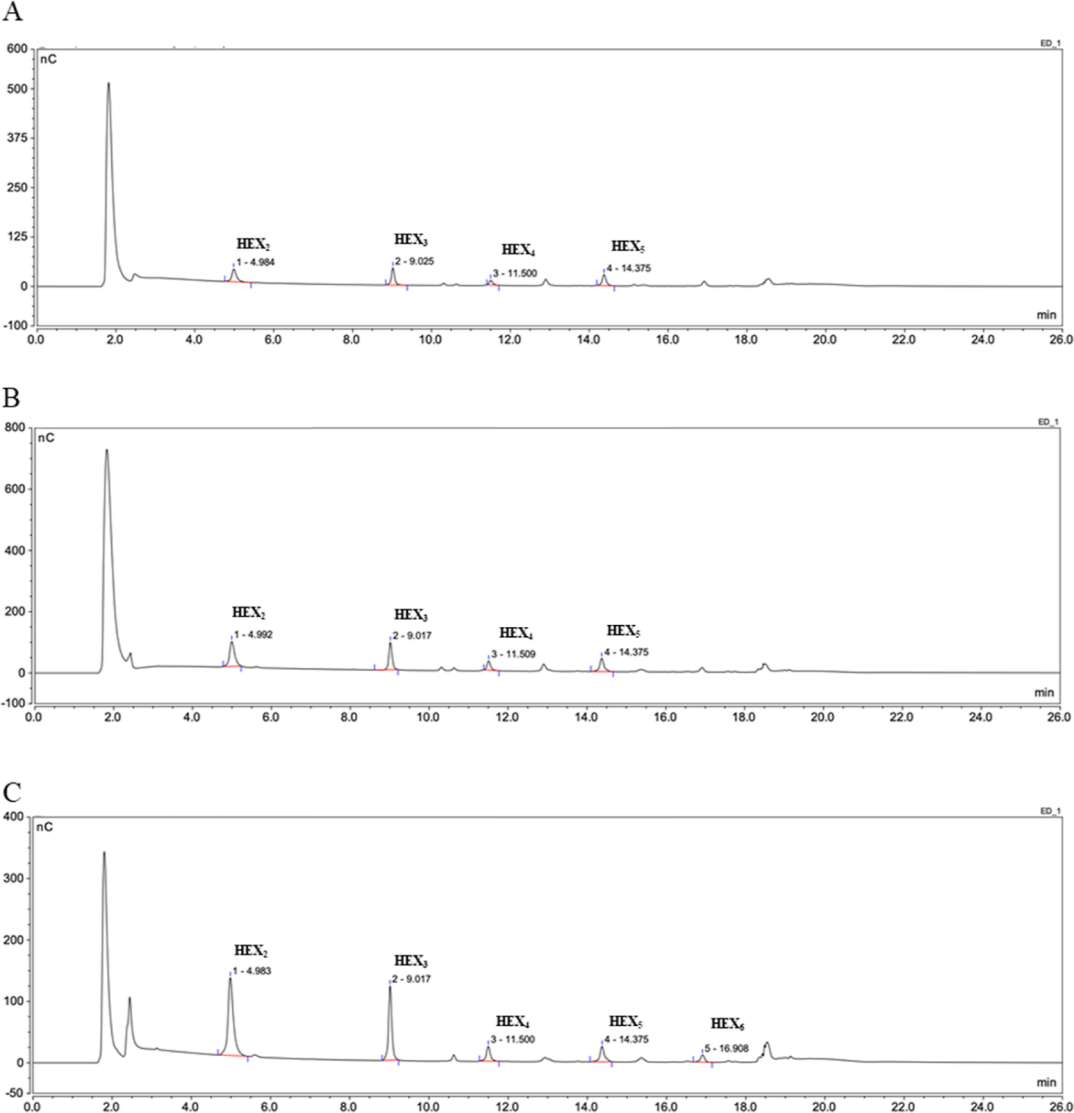
*Hv*EII variants ES2, ES3, and ES5 show (1,3)-β-d-glucanase activity. The *Hv*EII variants ES2 (A), ES3 (B), and ES5 (C) contain a hybrid protein sequence from *Hv*GII between amino acids 61–120, 120–176, and 237–307, respectively. Hydrolysis products after digestion of the (1,3)-β-d-glucan were detected using HPAEC-PAD. The (1,3)-β-d-glucanase activity of the ES5 variant was confirmed using MALDI-TOF MS (Supporting Information Figure S15). The commercially available oligosaccharide standards laminaribiose = Hex_2_; laminaritriose = Hex_3_; laminaritetraose = Hex_4_; laminaripentose = Hex_5_; laminarihexaose = Hex_6_ were used to assign individual peaks.

**Figure 7:**
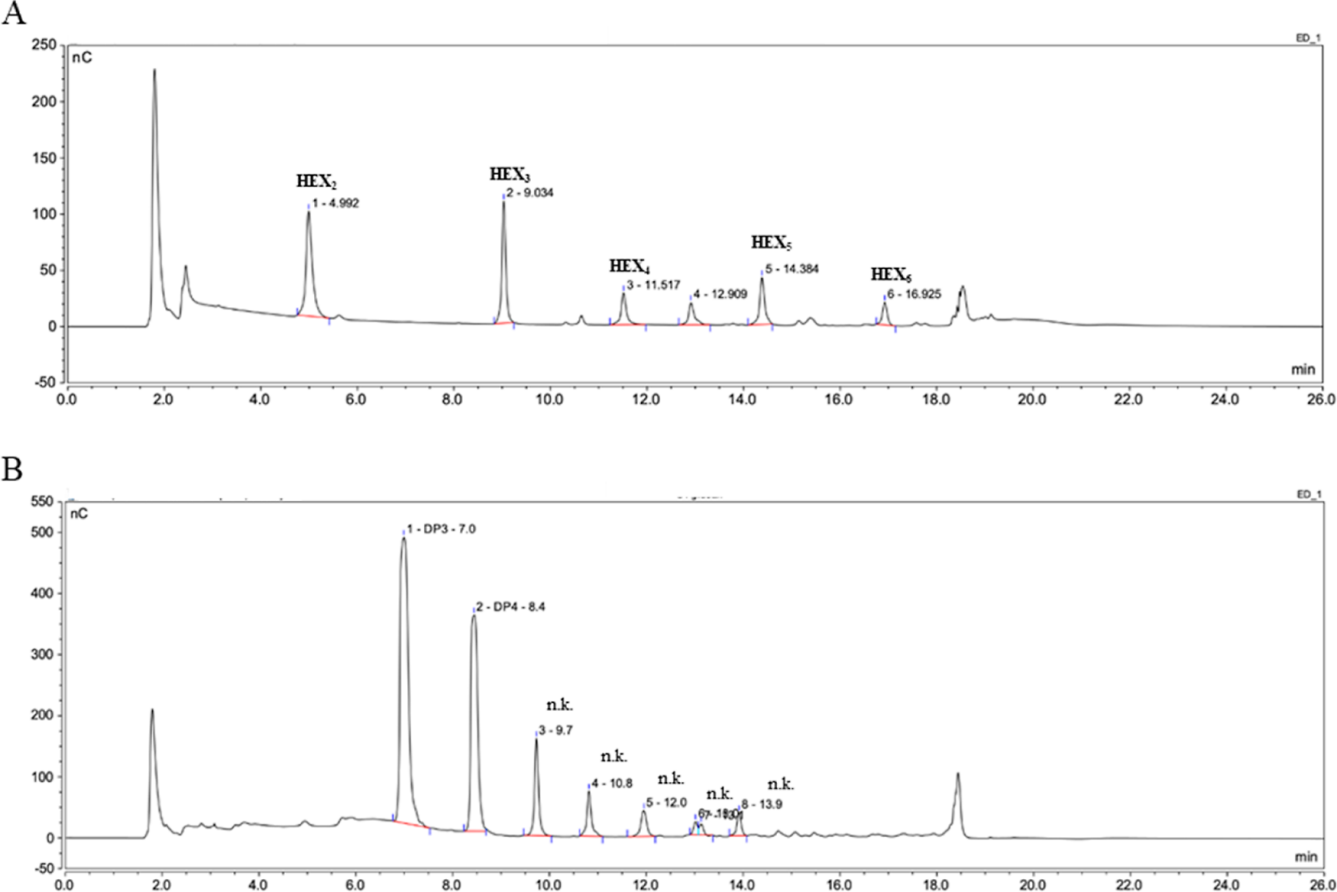
*Hv*EII variant ES4 is able to degrade both (1,3)- and (1,3;1,4)-β-d-glucans. Hydrolysis products after digestion of (1,3)-β-d-glucan (A) and (1,3;1,4)-β-d-glucan (B) were characterized by HPAEC-PAD with reference oligosaccharides. n.k. = not known.

During the phylogenetic study, we observed that the amino acid residues conserved between the (1,3)- and (1,3;1,4)-β-d-glucanases were evenly distributed along the whole sequences (Figures S1 and S3). To investigate if the change of substrate specificity was due to multiple mutations in one protein segment, we designed five *Hv*EII (ES1 to ES5) and five *Hv*GII (GS1 to GS5) variants with the amino-acid sequences changed approximately every 60 residues (Figure 5A,B). All of the *Hv*EII variants, except ES1 with substitution of the Ile^1^ to Ser^60^ segment by the equivalent segment of *Hv*GII (Figure 5), were successfully expressed by *E. coli*. The ES2, ES3, and ES5 variants of *Hv*EII had (1,3)-β-d-glucanase activity, but had completely lost the ability to hydrolyze (1,3;1,4)-β-d-glucans (Figures 5, 6, S11A,B,D and S12A–C). The conversion of the ES2, ES3, and ES5 variants to (1,3)-β-d-glucanases indicated that, in addition to key amino acid residues crucial for specifically binding to (1,3;1,4)-β-d-glucans, there were amino acid residues or segments of the enzyme that were not involved directly in substrate binding and hydrolysis but contribute to a change in the conformation of the substrate binding pocket that changes the specificity. In addition to (1,3)-β-d-glucanase activity, the ES4 variant maintained its ability to hydrolyze (1,3;1,4)-β-d-glucans with a specific activity of 7.7 ± 0.4 U/mg (Figures 7 and S11C), and with the DP3 and DP4 oligosaccharides accounting for 80.2% of the hydrolysis products with a DP3/DP4 ratio of 1.61:1 (Figure 5C). This segment did not contain any amino acid residues that were exposed in the enzymatic cleft. It is therefore likely that the ES4 variant slightly altered the overall shape of the cleft, not affecting the binding of (1,3;1,4)-β-d-glucans, but leading to a moderate loosening of the binding capacity of (1,3)-β-d-glucans and thereby broadening the enzyme’s substrate specificity.

Segment hybrid studies were also carried out with the *Hv*GII enzyme, but we found no changes in substrate specificity in the resultant GS variants (Figures S11E–I and S12D–H). (1,3)-β-d-Glucanase activity was found in the GS1, GS2 and GS5 variants, but this catalytic activity was abolished in the GS3 and GS4 variants (Figure 5C). Our segment hybrid studies clearly showed that substrate specificity can be switched from the (1,3;1,4)-β-d-glucanase activity of *Hv*EII to that of a (1,3)-β-d-glucanase, but not vice versa. Although the ES4 variant of *Hv*EII was shown to have dual specificities using HPAEC-PAD and MALDI-TOF MS (Figure S14), the specific activity toward (1,3)-β-d-glucans was too low to be quantified with the DNS colorimetric assay.

## Conclusions

In the present study, variants of barley (1,3;1,4)-β-d-glucanase *Hv*EII were produced that had (1,3)-β-d-glucanase activity. This is consistent with the similarity in structure between *Hv*EII and *Hv*GII and is evidence for ancestral (1,3)-β-d-glucanase being the ancestors of the (1,3;1,4)-β-d-glucans rather than for example (1,4)-β-d-glucanase (cellulases). Overall, the point mutation and segment hybrid experiments led to changes in or loosening the specificity of *Hv*EII, but the experiments did not result in any major changes to the specificity of *Hv*GII. This suggested that the evolution of *Hv*EII involved the acquisition of cumulative mutations necessary for this enzyme to become specialized to accommodate the new (1,3;1,4)-β-d-glucan substrate. The study supports the hypothesis of gene duplication of an ancestral (1,3)-β-d-glucanase that later became specialized to hydrolyze (1,3;1,4)-β-d-glucans.

Three of the *Hv*EII variants, ES2, ES3, and ES5, had switched substrate specificity from that of a (1,3;1,4)-β-d-glucanase to a (1,3)-β-d-glucanase, and two of the *Hv*EII variants, V6 and ES4, showed dual substrate specificities. We also generated the *Hv*EII variants V2, V5, V7, and V9 that liberated (1,3;1,4)-β-d-gluco-oligosaccharides with higher DPs than did the wild-type enzyme. It can be concluded, as expected, that the substrate specificities of these enzymes were not controlled by amino acid residues located in a single section of the substrate cleft but rather in multiple regions. More importantly, the sequence segment Gly^180^-Pro^236^ in *Hv*EII is not important in defining (1,3;1,4)-β-d-glucanase activity. The study provided clues as to how the (1,3;1,4)-β-d-glucanase *Hv*EII evolved from an ancestral (1,3)-β-d-glucanase, and provided new comparative information about the substrate specificities of the barley (1,3;1,4)-β-d-glucanase and those of other β-d-glucanases.

## Supporting Information Available

Phylogenetic study and protein structure analysis of barley (1,3;1,4)-β-d-glucanase *Hv*EII and (1,3)-β-d-glucanase *Hv*GII; substrate specificity study of *Hv*EII and *Hv*GII variants using HPAEC analysis (PDF)
