# Supplementary material for: Substrate Specificities of Variants of Barley (1,3)- and (1,3;1,4)-β-d-Glucanases Resulting from Mutagenesis and Segment Hybridization
Source: Biochemistry. 2024 Apr 10;63(9):1194–205. doi: 10.1021/acs.biochem.3c00673 (PMC11080057; doi:10.1021/acs.biochem.3c00673)
Supplement: Supplementary file 1 — bi3c00673_si_001.pdf [file bi3c00673_si_001.pdf]

Supporting Information

Substrate specificities of variants of barley (1,3)- and (1,3;1,4)-β-D-glucanases resulting from mutagenesis and segment hybridization

Mu-Rong Kao[a,b], Jake Parker[c,d], Daniel Oehme[d], Shu-Chieh Chang[a], Lin-Chen Cheng[a], Damao Wang[a,e], Vaibhav Srivastava[a], John M. Wagner[d], Philip J. Harris[f], Yves S. Y. Hsieh[a,b*]

[a] Division of Glycoscience, Department of Chemistry, School of Engineering Sciences in Chemistry, Biotechnology and Health, Royal Institute of Technology (KTH), AlbaNova University Centre, Stockholm, SE-10691, Sweden.

[b] School of Pharmacy, College of Pharmacy, Taipei Medical University, 250 Wuxing Street, Taipei, 11031, Taiwan.

[c] Australian Research Council Centre of Excellence in Plant Cell Walls, School of Agriculture, Food and Wine, University of Adelaide, Waite Campus, Glen Osmond, SA 5064, Australia.

[d] IBM Research Collaboratory for Life Sciences – Melbourne, Victoria, Australia.

[e] College of Food Science, Southwest University, Chongqing 400715, China.

[f] School of Biological Sciences, The University of Auckland, Auckland Mail Centre, Private Bag 92019, Auckland 1142, New Zealand.

[*]To whom correspondence should be addressed.

Email. yvhsieh@kth.se

Supplementary Figure 1: Phylogeny of angiosperm (1,3)-β-D-glucanases

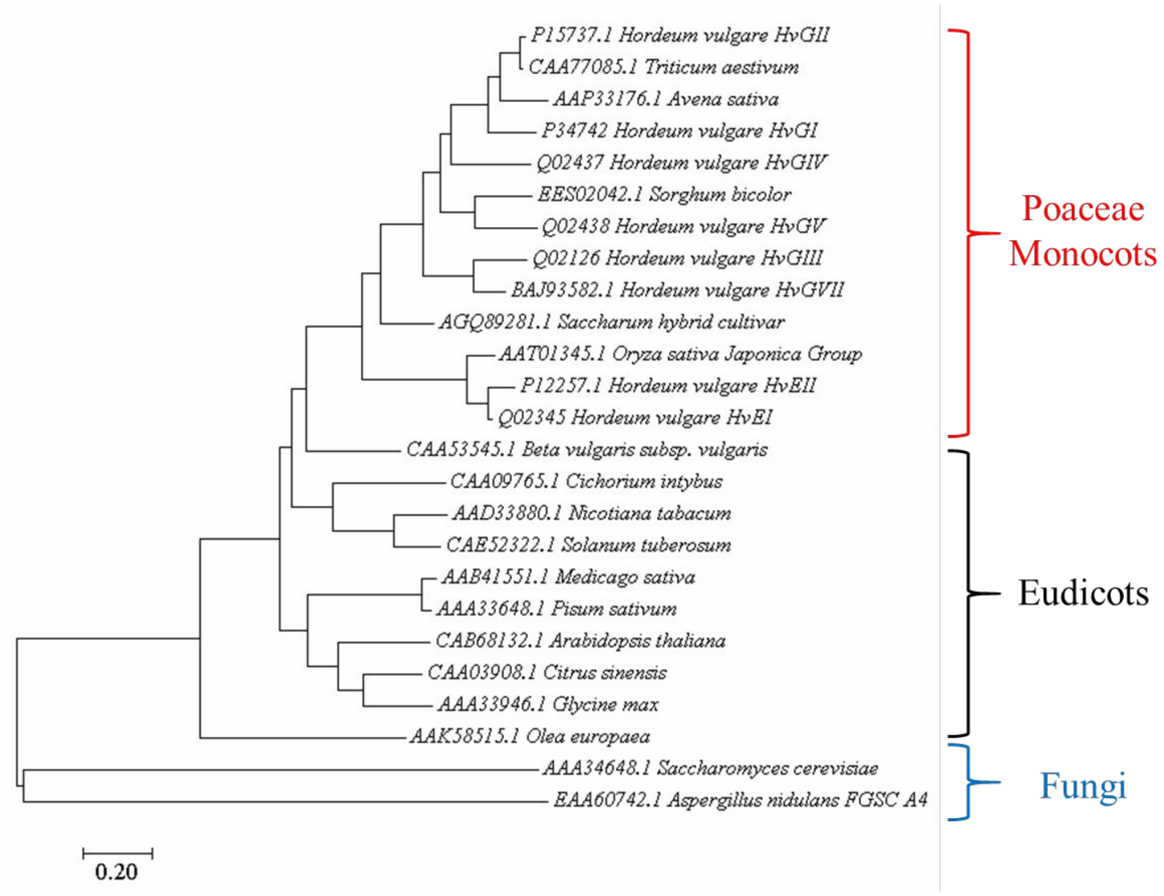

Figure S1: Phylogeny of angiosperm (1,3)-β-D-glucanases, with two fungal enzymes as an outgroup. Barley (1,3;1,4)-β-D-glucanase isoenzymes *Hv*EI and *Hv*EII are also included for comparison. The phylogeny was built using the Maximum Likelihood method in MEGA7. All the enzymes, including the barley (1,3;1,4)-β-D-glucanases *Hv*EI and *Hv*EII and two fungal (1,3)-β-D-glucanases (Table S1), are glycoside hydrolase family 17 (GH17) enzymes selected from the Carbohydrate-Active enZYmes database (www.cazy.org).

Supplementary Figure 2: Phylogeny of Poaceae (1,3;1,4)-β-D-glucanases and (1,3)-β-D-glucanases

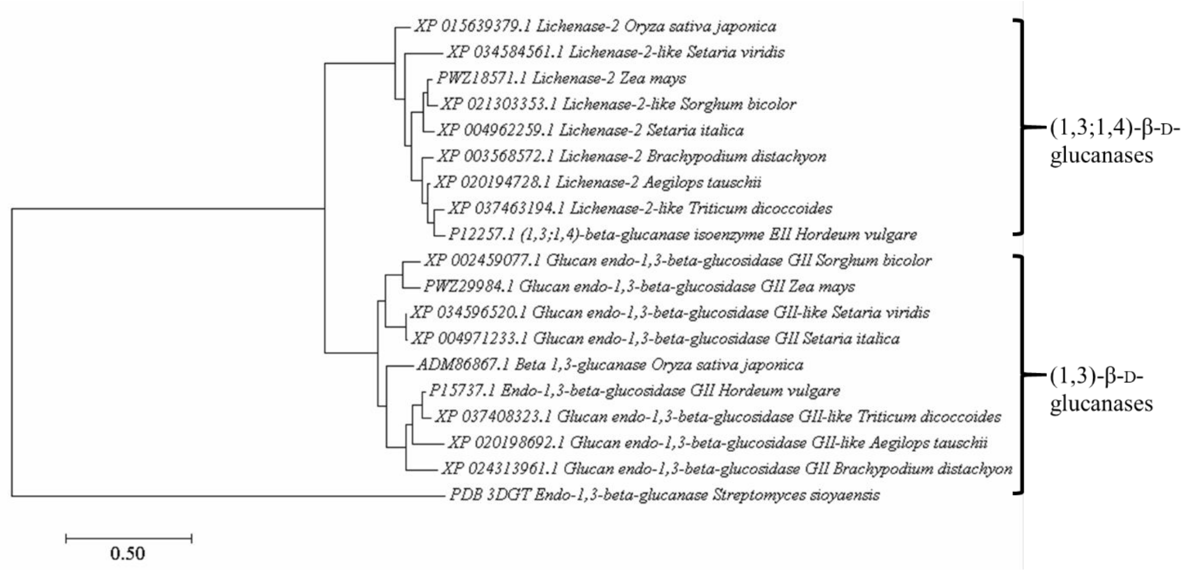

Figure S2: Phylogeny of Poaceae (1,3;1,4)-β-D-glucanases (lichenases) and (1,3)-β-D-glucanases, including *Hv*EII and *Hv*GII, with the bacterial (1,3)-β-D-glucanase from *Streptomyces sioyaensis* used as the outgroup. The phylogeny was obtained using the Maximum Likelihood method in MEGA7. NCBI accession numbers were used for the Poaceae enzymes and the PDB ID number for the bacterial enzyme (Table S1).

Supplementary figure 3: Sequence alignments of Poaceae (1,3)- and (1,3;1,4)-β-D-glucanases

```
XP_037463194.1          IGVCYGMSANNLPAASTVVGMFKSNGIKSMRLYAPDKAALQAVGGTGIYVVVG
XP_020194728.1          IGVCYGMSANNLPAASTVVSMFKSNGINSMRLYAPDQAALQAVGGTGVNVVVG
XP_003568572.1          IGVCYGMSANNLPAANTVVGMFKSNGINAMRLYAPDQAALQAVGGTGVSVAVG
XP_004962259.1          IGVCYGMSANNLPPASTVIDMYKANGISAMRLYAPDQGALQAVGGSGISVTVG
XP_015639379.1          IGVCYGMSANNLPPASSVVGMYRSNGITSMRLYAPDQAALQSVGGTGISVVVG
XP_034584561.1          IGVSYGMSGDNLPPASTVVGMYKANGIPLMRIYAPDQAALEAVGGTGIRVVVG
P12257.1 HvEII      1   IGVCYGMSANNLPAASTVVSMFKFNGIKSMRLYAPNQAALQAVGGTGINVVVG   53
P15737.1 HvGII      1   IGVCYGVIGNNLPSRSDVVQLYRSKGINGMRIYFADGQALSALRNSGIGLILD   53
ADM86867.1              IGVCYGVLGNNLPSRSEVVQLYKSKGINGMRIYYPDKEALNALRNSGIALILD
XP_004971233.1          IGVCYGVLGNNLPSRSDVVQLYRSRGINGMRIYFPDRQALDALRGSGMALILD
XP_034596520.1          IGVCYGVLGNNLPSRSDVVQLYRSRGINGMRIYFPDRQALDALRGSGMALILD
XP_020198692.1          VGVCYGVIANNLPPANEVVQLYRSKGITGMRIYFADANALSALRNSGISLILD
XP_037408323.1          IGVCYGVIGNNLPSRSDVVQLYRSKGINGMRIYFADGQALSALRNSGIGLILD
XP_024313961.1          IGVCYGVIGNNLPSRGDVVNLYRSKGINSMRIYFADAQALSALRNSGIALILD

XP_037463194.1          APNDVLSNLAASPAAAASWVRSNIQ-AYPKVSFRYVCVGNEVAGGATRNLVPA
XP_020194728.1          APNDVLSNLAASPAAAASWVRSNIQ-AYPKVSFRYVCVGNEVAGGATQNLVPA
XP_003568572.1          APNDVLSNIASSPAAAASWVRNNIQ-AYPSVSFRYVVVGNEVAGGATQNLVPA
XP_004962259.1          APNDVLSNIAASPAAAASWVRNNIQ-AYPSVSFRYICVGNEVAGGAAQNLAPA
XP_015639379.1          APNDVLSNLAASPAAAASWVRNNIQ-AYPSVSFRYVAVGNEVAGGATSSLVPA
XP_034584561.1          APNDVLSSLAASPAAAAWVRNNIA-AYPDVTFRCVCVGNEVEGGAAQNLVPA
P12257.1 HvEII     54   APNDVLSNLAASPAAAASWVKSNIQ-AYPKVSFRYVCVGNEVAGGATRNLVPA  105
P15737.1 HvGII     54   IGNDQLANIAASTSNAASWVQNNVRPYYPAVNIKYIAAGNEVQGGATQSILPA  106
ADM86867.1              VG-DQLSNLAASSSNAAAWVRDNVRPYYPAVNIKYIAVGNEVEGGATSSILPA
XP_004971233.1          TGNDVLGQLASSPSSAASWVQSNVRPYYPAVNIKYIAVGNEVAGSATQSILPA
XP_034596520.1          TGNDVLGQLASSPSSAASWVQNNVRPYYPAVNIKYIAVGNEVAGSATQSILPA
XP_020198692.1          VGNDQLANLAASTSNAASWVQKNVRPYYPAVNIKYIAAGNEVQGGATQSIVPA
XP_037408323.1          IGNDQLSNIAASTSNAASWVQNNVRPYYPAVNIKYIAAGNEVQGGDTQSIVPA
XP_024313961.1          IGNDNLAGIASSASNAATWVNNNVKPYYPAVNIKYIAAGNEILGGATGSIVPA

XP_037463194.1          MKNVHGALTSAGLG-HIKVTTSVSQAILGVYSPPSAGSFTAEAAAFMGPVVQF
XP_020194728.1          MKNVQGALASAGLG-HIKVTTSVSQAILGVYSPPSAGSFTGEADAFMGPVVQF
XP_003568572.1          MKNVHSALASAGLG-HIKVTTSVSQAILGVYSPPSAGSFTGEADAFMGPVVQF
XP_004962259.1          MENVHAALAAAGLG-HIKVTTSVSQAILGVYSPPSAAEFTGEAKGYMGPVLSF
XP_015639379.1          MENVRGALVSAGLG-HIKVTTSVSQALLAVYSPPSAAEFTGESQAFMAPVLSF
XP_034584561.1          MENIRAALAAAGLD-GIKVTTSVSQAILGGYKPPSAAEFTDEAQGFMGPVLEF
P12257.1 HvEII    106   MKNVHGALVAAGLG-HIKVTTSVSQAILGVFSPPSAGSFTGEAAAFMGPVVQF  157
P15737.1 HvGII    107   MRNLNAALSAAGLG-AIKVSTSIRFDEVANSFPPSAGVFKN---AYMTDVARL  155
ADM86867.1              IRNVNSALASSGLG-AIKASTAVKFDVISNSYPPSAGVFRD---AYMKDIARY
XP_004971233.1          MRNLNAALAAAGLG-SIKVSTSVQSNVIANSFPPSSGVFAQ---GYMVEIARY
XP_034596520.1          MRNLNAALAAAGLG-SIKVSTSVQSNVIANSFPPSSGVFAQ---GYMVEIARY
XP_020198692.1          MRNLNAALSAAGLG-AIKVSTSIRFDEVDKSFPPSDGVFKN---AYMTDVARL
XP_037408323.1          MRNLNAVLSAAGLS-AIKVSTSIRFDAVANSFPPSAGVFAQ---SYMTDVARL
XP_024313961.1          MRNLNAALASAGLGDRIKVSTSIRFDAVADSFPPSKGVFKD---AYMSDVARL
```

```
XP_037463194.1         LARTRAPLMANIYPYLAWAYNPSAMDMSYALFTASGTVVQDG-GFGYQNLFDT
XP_020194728.1         LARTGAPLMANIYPYLAWAYNPSAMDMSYALFTASGTVVQDG-SYGYQNLFDT
XP_003568572.1         LASAGSPLMANIYPYLAWAYNPSAMDMSYALFTASGTVVQDG-AYGYQNLFDT
XP_004962259.1         LARTGSPLMANIYPYLAWAYNPSAMDMSYALFTSKGTVVQDG-AYGYQNLFDT
XP_015639379.1         LARTGAPLLANIYPYFSYTYSQGSVDVSYALFTAAGTVVQDG-AYGYQNLFDT
XP_034584561.1         LARTGAPLMASIYPYFTYATNPSAMDLSYALFTAPGTVLQDG-TYGYQNLFDA
P12257.1 HvEII     158 LARTNAPLMANIYPYLAWAYNPSAMDMGYALFNASGTVVRDG-AYGYQNLFDT 209
P15737.1 HvGII     156 LASTGAPLLANVYPYFAYRDNPGSISLNYATFQPGTTVRDQNNGLTYTSLFDA 208
ADM86867.1             LASTGAPLLANVYPYFAYRGNPRDISLNYATFRPGTTVRDPNNGLTYTNLFDA
XP_004971233.1         LASTGAPLLANVYPYFAYRGNPRDISLGYATFQPGTTVRDGGNGLTYTNLFDA
XP_034596520.1         LASTGAPLLANVYPYFAYRGNPRDISLGYATFQPGTTVRDGGNGLTYTNLFDA
XP_020198692.1         LASTGAPLLANVYPYFAYKRDPQNIKLNYATFRPGPTVRDDKNGLTYTCLFDA
XP_037408323.1         LASTGAPLLANVYPYFAYRDNPRDISLNYATFQPGTTVRDQNNGLTYTSLFDA
XP_024313961.1         LASTGAPLLANVYPYFAYRDSPSAIQLNYATFQPGTQVRDDGNGLVYTNLFDA

XP_037463194.1         TMDAFYTAMAKHGGSNVKLVVSESGWPSGGGTAATPANARFYNQHLINHVGRG
XP_020194728.1         TVDAFYTAMAKHGGSNVKLVVSESGWPSGGGTAATPANARIYNQYLINHVGRG
XP_003568572.1         TVDAFYNAMAKHGGNGVKLVVSESGWPSAGGTAATPANARVYNQYLINHVGRG
XP_004962259.1         TVDAFYFAMGRHGGSGVPLVVSESGWPSGGGEQANAANARIYNQYLINHVGRG
XP_015639379.1         TVDAFYAAMAKHGGSGVSLVVSETGWPSAGGMSASPANARIYNQNLINHVGRG
XP_034584561.1         TVDSFYVAMANHGGAGVTLVVSESGWPSAGGVAASPENAALYNQNLINHVGRG
P12257.1 HvEII     210 TVDAFYTAMGKHGGSSVKLVVSESGWPSGGGTAATPANARFYNQHLINHVGRG 262
P15737.1 HvGII     209 MVDAVYAALEKAGAPAVKVVVSESGWPSAGGFAASAGNARTYNQGLINHVGGG 261
ADM86867.1             MMDAVYAALEKAGAGNVRVVVSESGWPSAGGFGASVDNARAYNQGLIDHVGRG
XP_004971233.1         MVDATVAALEKAGAPNVRIVISESGWPSAGGFGASVENARNYNQGLIDHVGRG
XP_034596520.1         MVDATVAALEKAGAPNVRIVISESGWPSAGGFGASVENARNYNQGLIDHVGRG
XP_020198692.1         MVDAVVAALEKAGAPAVRVVVSESGWPSASGFAATADNARAYNQGLIDHVGGG
XP_037408323.1         MVDAVYAALEKAGAPGVKVVISESGWPSAGGFAASADNARTYNQGLINHVGGG
XP_024313961.1         MVDAVHAAMEKAGAGGVKVVVSESGWPSDGGFAANADNARAYNQGLIDHVGKG

XP_037463194.1         TPRHQGAIETYIFAMFNENQKTS-GVEQHWGLFYPNMQHVYPINF
XP_020194728.1         TPRHPGAIETYVFSMFNENQKDS-GVEQNWGLFYPNMQHVYPISF
XP_003568572.1         TPRHPGAIETYVFSMFNENQKDS-GVEQNWGLFYPNMQHVYPISF
XP_004962259.1         TPRHPGGIETYLFSMFNENQKDS-GVEQNWGLFYPNMQHVYPISF
XP_015639379.1         TPRHPGAIETYVFSMFNENQKDA-GVEQNWGLFYPNMQHVYPISF
XP_034584561.1         TPRHPGAIETILFSMFNENLKES-GVEQNWGLFYPNKQRVYPISF
P12257.1 HvEII     263 TPRHPGAIETYIFAMFNENQKDS-GVEQNWGLFYPNMQHVYPINF 306
P15737.1 HvGII     262 TPKKREALETYIFAMFNENQKTGDATERSFGLFNPDKSPAYNIQF 306
ADM86867.1             TPKRPGALEAYIFAMFNENQKNGDPTERNFGLFYPNKSPVYPIRF
XP_004971233.1         TPKRSGALDTFIFAMFNENQKSGDPTERNFGLFYPNKQPVYSIRF
XP_034596520.1         TPKRSGAMETFIFAMFNENQKSGDPTERNFGLFYPNKQPVYSIRF
XP_020198692.1         TPKRRGALETYIFAMFNENFKRGELVEKHFGLFNPDKSPAYPIRF
XP_037408323.1         TPKKRQALETYIFAMFNENQKTGDATERSFGLFNPDKSPAYNIQF
XP_024313961.1         TPKKPGPLEAYIFAMFNENQKDGNAVERNFGLFKPDKSPAYDIRF
```

Figure S3: Sequence aligments of Poaceae (1,3)- and (1,3;1,4)-β-D-glucanases. The NCBI accession numbers corresponding to (1,3)- and (1,3;1,4)-β-D-glucanases (from Figure S2) are in blue and in red, respectively**.** Identical residues in the (1,3)- and (1,3;1,4)-β-D-glucanases are highlighted in yellow. Among the identical residues, catalytic residues are in bold red, and hydrophobic and negatively charged amino acid residues surrounding the substrate cleft are in

bold black. Conserved hydrophobic residues surrounding the substrate cleft in (1,3;1,4)-β-D-glucanases and in (1,3)-β-D-glucanases are highlighted in brown and green, respectively. The amino acid residue at the same position as the Y177 in *Hv*EII is highlighted in blue in (1,3)-β-D-glucanases.

Supplementary figure 4: Molecular dynamic-assisted substrate docking on *Hv*EII and *Hv*GII

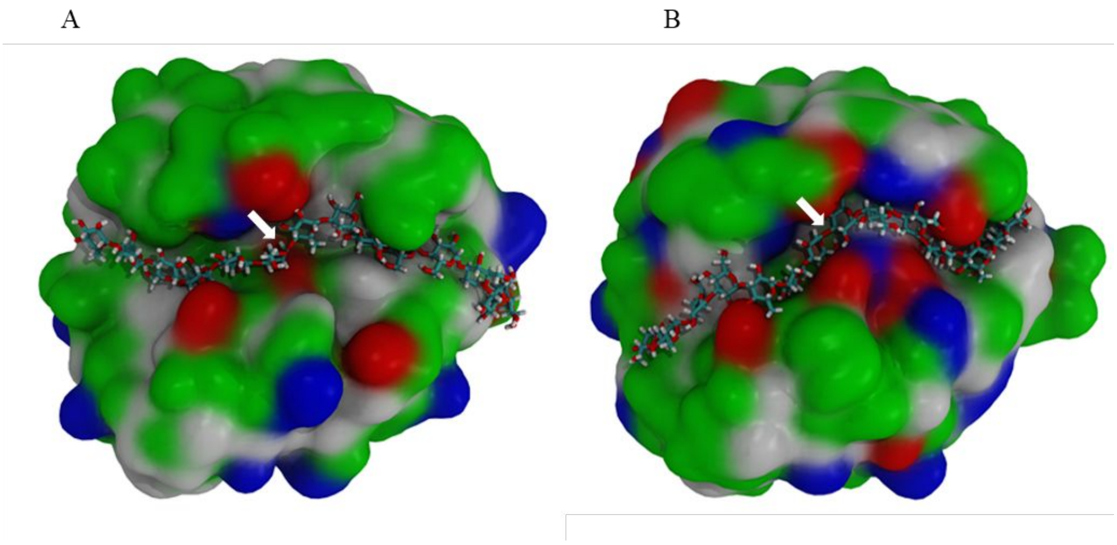

Figure S4: Molecular dynamic-assisted substrate docking was performed with a DP14 (1,3;1,4)-β-D-glucan oligosaccharide for *Hv*EII (A) and with a DP14 (1,3)-β-D-glucan oligosaccharide for *Hv*GII (B). The β-D-glucans oligosaccharides were positioned in the direction from the non-reducing end to the reducing end, which corresponds to from the left to the right of the substrate cleft. Amino acids coloured "red" are negatively charged residues, "blue" are positively charged residues, "green" are hydrophobic residues, and "grey" are all other amino acid residues. The cleavage site is indicated with a white arrow.

Supplementary figure 5: Superposition of the protein structures of barley (1,3)-β-D-glucanase *Hv*GII and potato (1,3)-β-glucanase GLUB20-2

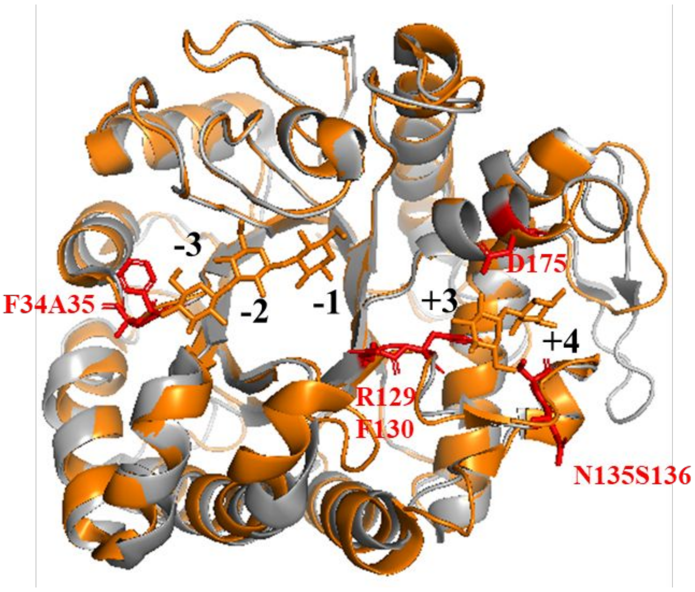

Figure S5: Barley (1,3)-β-D-glucanase *Hv*GII (PDB ID: 1GHS, in grey) and potato (1,3)-β-D-glucanase GLUB20-2 (PDB ID: 4GZJ, in orange) have been superposed. The amino acid residues F34, A35, R129, F130, N15, S136, and D175 of *Hv*GII are represented as red sticks. The glucosyl residues at different substrate sub-sites in the GLUB20-2 co-crystal structure are numbered and represented as orange sticks.

Supplementary figure S6: pET3a-*Hv*EII and pET3a-*Hv*GII constructs

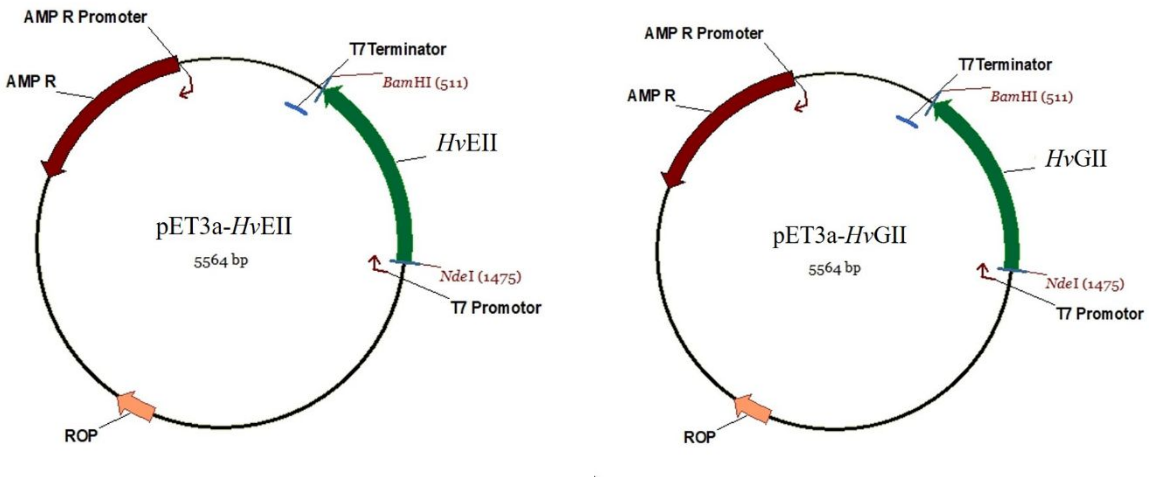

Figure S6: pET3a-*Hv*EII and pET3a-*Hv*GII constructs. These expression vectors were used for the heterogeneous expression of barley (1,3;1,4)-β-D-glucanase *Hv*EII and barley (1,3)-β-D-glucanase *Hv*GII in *E. coli* strain BL21.

Supplementary figure S7: Amino acid sequences of WT *Hv*EII and variants generated by mutagenesis

A) Sequence of WT *Hv*EII (yield: about 0.25 mg/L of culture; purity > 95%; specific activity towards the (1,3;1,4)-β-D-glucan: 10.2 ± 0.4 U/mg):

IGVCYGMSANNLPAASTVVSMFKFNGIKSMRLYAPNQAALQA
VGGTGINVVVGAPNDVLSNLAASPAAAASWVKSNIQAYPKVS
FRYVCVGNEVAGGATRNLVPAMKNVHGALVAAGLGHIKVTT
SVSQAILGVFSPPSAGSFTGEAAAFMGPVVQFLARTNAPLMAN
IYPYLAWAYNPSAMDMGYALFNASGTVVRDGAYGYQNLFDT
TVDAFYTAMGKHGGSSVKLVVSESGWPSGGGTAATPANARF
YNQHLINHVGRGTPRHPGAIETYIFAMFNENQKDSGVEQNWG
LFYPNMQHVYPINF

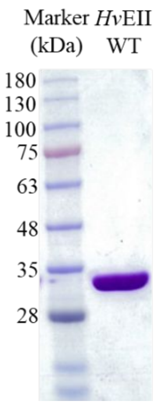

B) Sequence of *Hv*EII E93A:
IGVCYGMSANNLPAASTVVSMFKFNGIKSMRLYAPNQAALQAVGGTGINVVVGAP
NDVLSNLAASPAAAASWVKSNIQAYPKVSFRYVCVGNAVAGGATRNLVPAMKNV
HGALVAAGLGHIKVTTSVSQAILGVFSPPSAGSFTGEAAAFMGPVVQFLARTNAPLM
ANIYPYLAWAYNPSAMDMGYALFNASGTVVRDGAYGYQNLFDTTVDAFYTAMGK
HGGSSVKLVVSESGWPSGGGTAATPANARFYNQHLINHVGRGTPRHPGAIETYIFAM
FNENQKDSGVEQNWGLFYPNMQHVYPINF

C) Sequence of *Hv*EII variant V1 (A34F, P35A):
IGVCYGMSANNLPAASTVVSMFKFNGIKSMRLYFANQAALQAVGGTGINVVVGAP
NDVLSNLAASPAAAASWVKSNIQAYPKVSFRYVCVGNEVAGGATRNLVPAMKNVH
GALVAAGLGHIKVTTSVSQAILGVFSPPSAGSFTGEAAAFMGPVVQFLARTNAPLMA
NIYPYLAWAYNPSAMDMGYALFNASGTVVRDGAYGYQNLFDTTVDAFYTAMGKH
GGSSVKLVVSESGWPSGGGTAATPANARFYNQHLINHVGRGTPRHPGAIETYIFAMF
NENQKDSGVEQNWGLFYPNMQHVYPINF

D) Sequence of *Hv*EII variant V2 (A34F, P35A, N36D, Q37G, A38Q):
IGVCYGMSANNLPAASTVVSMFKFNGIKSMRLYFADAQALQAVGGTGINVVVGAP
NDVLSNLAASPAAAASWVKSNIQAYPKVSFRYVCVGNEVAGGATRNLVPAMKNVH

GALVAAGLGHIKVTTSVSQAILGVFSPPSAGSFTGEAAAFMGPVVQFLARTNAPLMA
NIYPYLAWAYNPSAMDMGYALFNASGTVVRDGAYGYQNLFDTTVDAFYTAMGKH
GGSSVKLVVSESGWPSGGGTAATPANARFYNQHLINHVGRGTPRHPGAIETYIFAMF
NENQKDSGVEQNWGLFYPNMQHVYPINF

E) Sequence of *Hv*EII variant V3 (Y177D; yield: about 0.23 mg/L of culture; purity about 94%; specific activity towards the (1,3;1,4)-β-D-glucan: 9.3 ± 0.5 U/mg):

IGVCYGMSANNLPAASTVVSMFKFNGIKSMRLYAPNQAALQA
VGGTGINVVVGAPNDVLSNLAASPAAAASWVKSNIQAYPKVS
FRYVCVGNEVAGGATRNLVPAMKNVHGALVAAGLGHIKVTT
SVSQAILGVFSPPSAGSFTGEAAAFMGPVVQFLARTNAPLMAN
IYPYLAWADNPSAMDMGYALFNASGTVVRDGAYGYQNLFDT
TVDAFYTAMGKHGGSSVKLVVSESGWPSGGGTAATPANARF
YNQHLINHVGRGTPRHPGAIETYIFAMFNENQKDSGVEQNWG
LFYPNMQHVYPINF

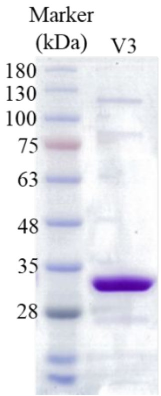

F) Sequence of *Hv*EII variant V4 (Y177G; yield: about 0.08 mg/L of culture; purity about 82%; specific activity towards the (1,3;1,4)-β-D-glucan: 7.5 ± 1.3 U/mg):
IGVCYGMSANNLPAASTVVSMFKFNGIKSMRLYAPNQAALQA
VGGTGINVVVGAPNDVLSNLAASPAAAASWVKSNIQAYPKVS
FRYVCVGNEVAGGATRNLVPAMKNVHGALVAAGLGHIKVTT
SVSQAILGVFSPPSAGSFTGEAAAFMGPVVQFLARTNAPLMAN
IYPYLAWAGNPSAMDMGYALFNASGTVVRDGAYGYQNLFDT
TVDAFYTAMGKHGGSSVKLVVSESGWPSGGGTAATPANARF
YNQHLINHVGRGTPRHPGAIETYIFAMFNENQKDSGVEQNWG
LFYPNMQHVYPINF

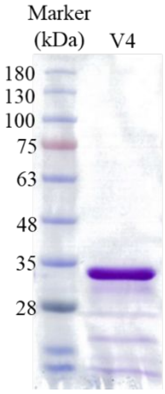

G) Sequence of *Hv*EII variant V5 (L173F, W175Y, A176R):
IGVCYGMSANNLPAASTVVSMFKFNGIKSMRLYAPNQAALQAVGGTGINVVVGAP
NDVLSNLAASPAAAASWVKSNIQAYPKVSFRYVCVGNEVAGGATRNLVPAMKNVH
GALVAAGLGHIKVTTSVSQAILGVFSPPSAGSFTGEAAAFMGPVVQFLARTNAPLMA
NIYPYFAYRYNPSAMDMGYALFNASGTVVRDGAYGYQNLFDTTVDAFYTAMGKH

GGSSVKLVVSESGWPSGGGTAATPANARFYNQHLINHVGRGTPRHPGAIETYIFAMF
NENQKDSGVEQNWGLFYPNMQHVYPINF

H) Sequence of HvEII variant V6 (S128R, Q129; yield: about 0.46 mg/L of culture; purity about 86%; specific activity towards the (1,3;1,4)-β-D-glucan: 9.9 ± 0.3 U/mg):

IGVCYGMSANNLPAASTVVSMFKFNGIKSMRLYAPNQAALQA
VGGTGINVVVGAPNDVLSNLAASPAAAASWVKSNIQAYPKVS
FRYVCVGNEVAGGATRNLVPAMKNVHGALVAAGLGHIKVTT
SVRFAILGVFSPPSAGSFTGEAAAFMGPVVQFLARTNAPLMAN
IYPYLAWAYNPSAMDMGYALFNASGTVVRDGAYGYQNLFDT
TVDAFYTAMGKHGGSSVKLVVSESGWPSGGGTAATPANARF
YNQHLINHVGRGTPRHPGAIETYIFAMFNENQKDSGVEQNWG
LFYPNMQHVYPINF

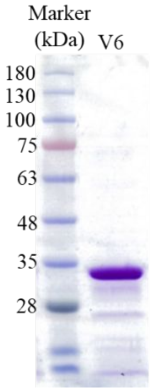

I) Sequence of *Hv*EII variant V7 (L1332V, G133A, V134N, F135S, S136):

IGVCYGMSANNLPAASTVVSMFKFNGIKSMRLYAPNQAALQAVGGTGINVVVGAP
NDVLSNLAASPAAAASWVKSNIQAYPKVSFRYVCVGNEVAGGATRNLVPAMKNVH
GALVAAGLGHIKVTTSVSQAIVANSFPPSAGSFTGEAAAFMGPVVQFLARTNAPLMA
NIYPYLAWAYNPSAMDMGYALFNASGTVVRDGAYGYQNLFDTTVDAFYTAMGKH
GGSSVKLVVSESGWPSGGGTAATPANARFYNQHLINHVGRGTPRHPGAIETYIFAMF
NENQKDSGVEQNWGLFYPNMQHVYPINF

J) Sequence of *Hv*EII variant V8 (V134N, D135S, S136; yield: about 0.1 mg/L of culture; purity about 84%; specific activity towards the (1,3;1,4)-β-D-glucan: 6.7 ± 1.3 U/mg):

IGVCYGMSANNLPAASTVVSMFKFNGIKSMRLYAPNQAALQA
VGGTGINVVVGAPNDVLSNLAASPAAAASWVKSNIQAYPKVS
FRYVCVGNEVAGGATRNLVPAMKNVHGALVAAGLGHIKVTT
SVSQAILGNSFPPSAGSFTGEAAAFMGPVVQFLARTNAPLMAN
IYPYLAWAYNPSAMDMGYALFNASGTVVRDGAYGYQNLFDT
TVDAFYTAMGKHGGSSVKLVVSESGWPSGGGTAATPANARF
YNQHLINHVGRGTPRHPGAIETYIFAMFNENQKDSGVEQNWG
LFYPNMQHVYPINF

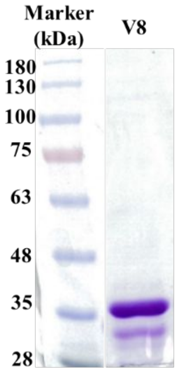

K) Sequence of *Hv*EII variant V9 (A34F, P35A, Y177D):

IGVCYGMSANNLPAASTVVSMFKFNGIKSMRLYFANQAALQAVGGTGINVVVGAP
NDVLSNLAASPAAAASWVKSNIQAYPKVSFRYVCVGNEVAGGATRNLVPAMKNVH
GALVAAGLGHIKVTTSVSQAILGVFSPPSAGSFTGEAAAFMGPVVQFLARTNAPLMA
NIYPYLAWADNPSAMDMGYALFNASGTVVRDGAYGYQNLFDTTVDAFYTAMGKH
GGSSVKLVVSESGWPSGGGTAATPANARFYNQHLINHVGRGTPRHPGAIETYIFAMF
NENQKDSGVEQNWGLFYPNMQHVYPINF

L) Sequence of *Hv*EII variant V10 (A34F, P35A, S128R, Q129, Y177D):

IGVCYGMSANNLPAASTVVSMFKFNGIKSMRLYFANQAALQAVGGTGINVVVGAP
NDVLSNLAASPAAAASWVKSNIQAYPKVSFRYVCVGNEVAGGATRNLVPAMKNVH
GALVAAGLGHIKVTTSVRFAILGVFSPPSAGSFTGEAAAFMGPVVQFLARTNAPLMA
NIYPYLAWADNPSAMDMGYALFNASGTVVRDGAYGYQNLFDTTVDAFYTAMGKH
GGSSVKLVVSESGWPSGGGTAATPANARFYNQHLINHVGRGTPRHPGAIETYIFAMF
NENQKDSGVEQNWGLFYPNMQHVYPINF

M) Sequence of *Hv*EII variant V11(A34F, P35A, N36D, Q37G,A38Q, S128R, Q129, Y177D):

IGVCYGMSANNLPAASTVVSMFKFNGIKSMRLYFADAQALQAVGGTGINVVVGAP
NDVLSNLAASPAAAASWVKSNIQAYPKVSFRYVCVGNEVAGGATRNLVPAMKNVH
GALVAAGLGHIKVTTSVRFAILGVFSPPSAGSFTGEAAAFMGPVVQFLARTNAPLMA
NIYPYLAWADNPSAMDMGYALFNASGTVVRDGAYGYQNLFDTTVDAFYTAMGKH
GGSSVKLVVSESGWPSGGGTAATPANARFYNQHLINHVGRGTPRHPGAIETYIFAMF
NENQKDSGVEQNWGLFYPNMQHVYPINF

Figure S7: Amino acid sequences of WT *Hv*EII and variants. Amino acid residues substituted with the residues from *Hv*GII are colored red. A) WT *Hv*EII, B) inactivated mutant *Hv*EII E93A, C) variant V1, D) variant V2, E) variant V3, F) variant V4, G) variant V5, H) variant V6, I) variant V7, J) variant V8, K) variant V9, L) variant V10, M) variant V11. A, E, F, H and J also show SDS-PAGE gels of the purified proteins to indicate their molecular weights compared with a molecular weight marker ladder.

Supplementary figure S8: Hydrolysis products of WT *Hv*EII and variants generated by mutagenesis analysed by HPAEC

A) Substrate only

(1,3)-β-D-glucan only

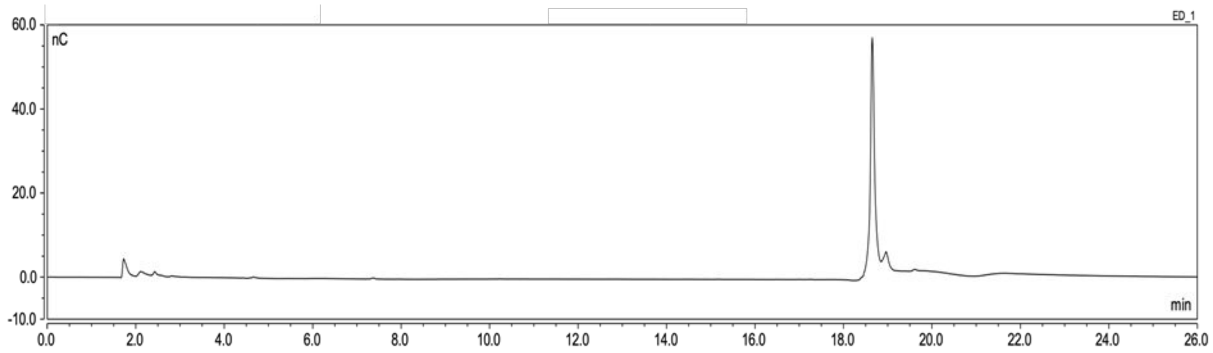

(1,3;1,4)-β-D-glucan only

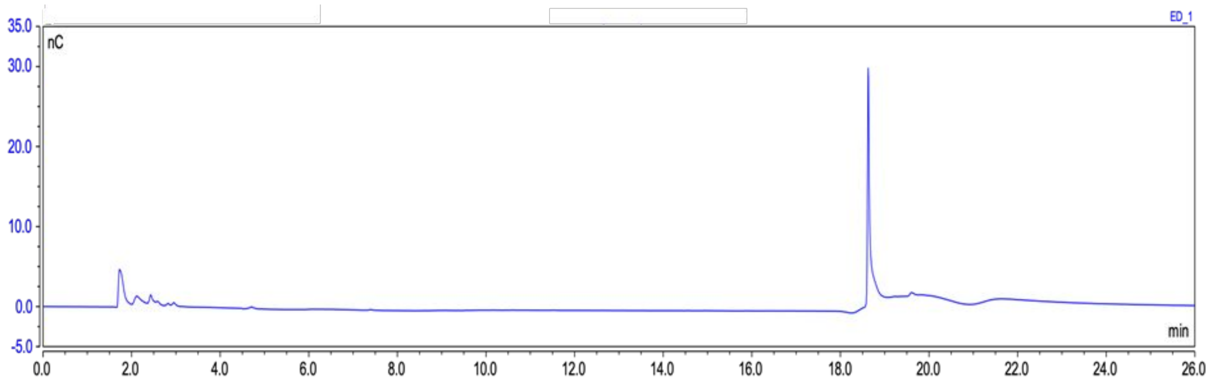

B) Wild-type *Hv*EII

Wild-type *Hv*EII + (1,3)-β-D-glucan

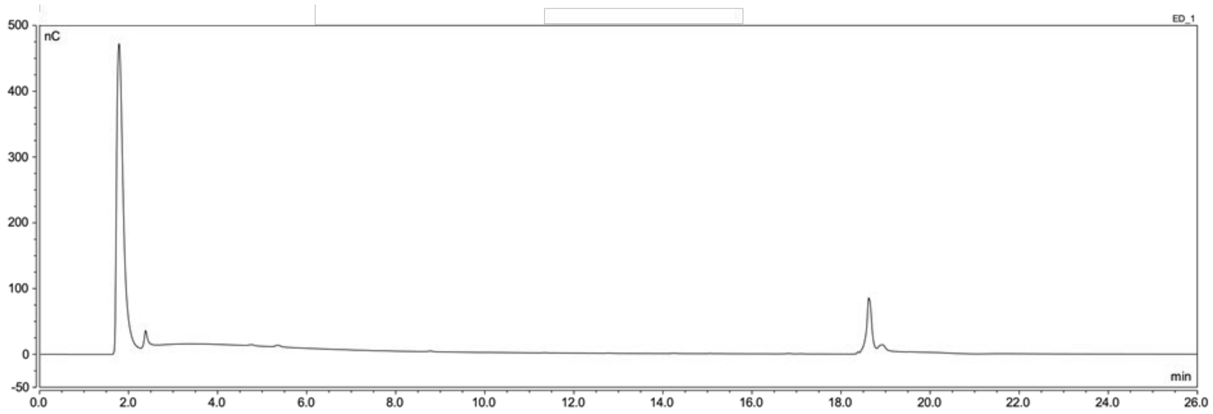

Wild-type *Hv*EII + (1,3;1,4)-β-D-glucan

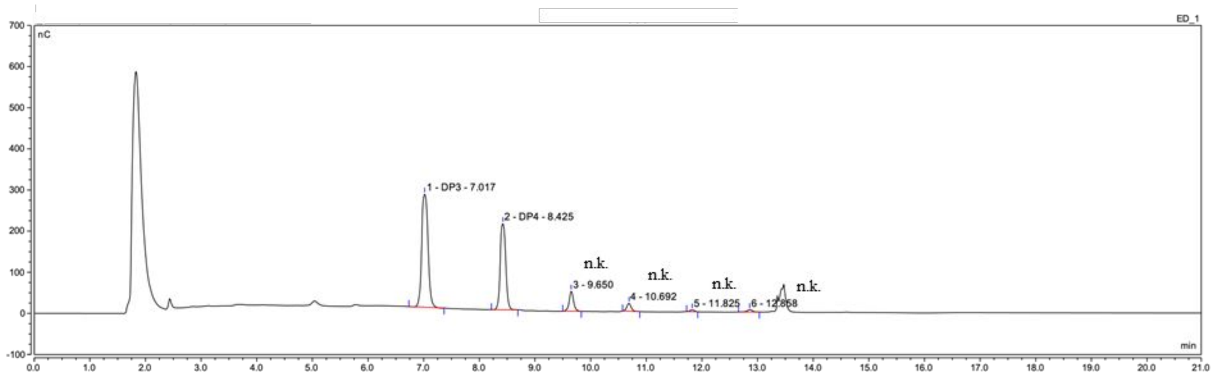

C) *Hv*EII E93A mutant

*Hv*EII mutant E93A + (1,3)-β-D-glucan

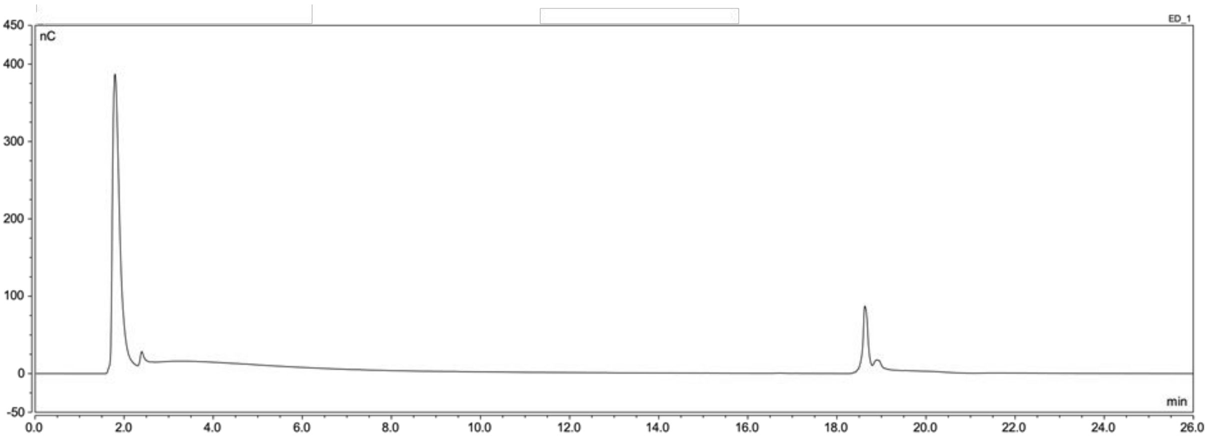

*Hv*EII mutant E93A + (1,3;1,4)-β-D-glucan

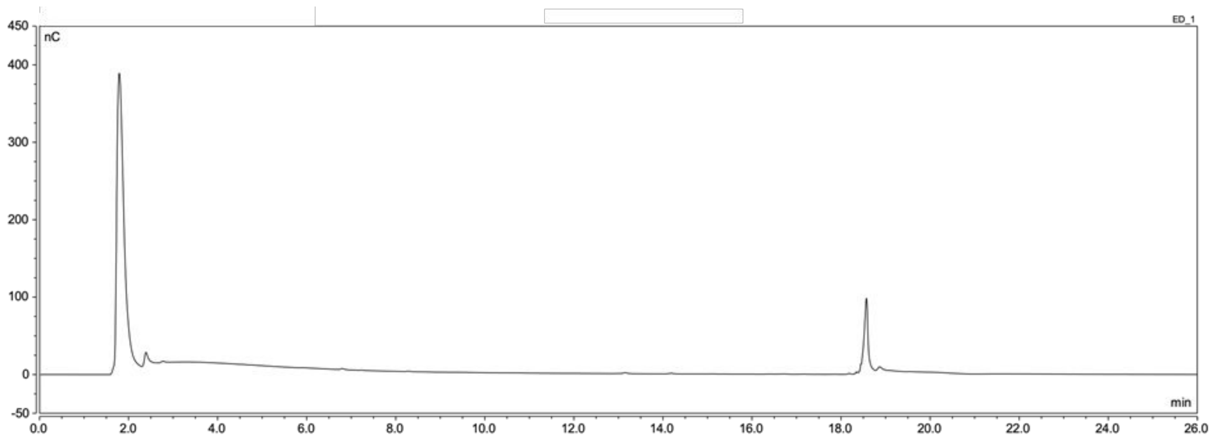

D) *Hv*EII variant V1 (A34F, P35A):

*Hv*EII V1 (A34F, P35A) + (1,3)-β-D-glucan

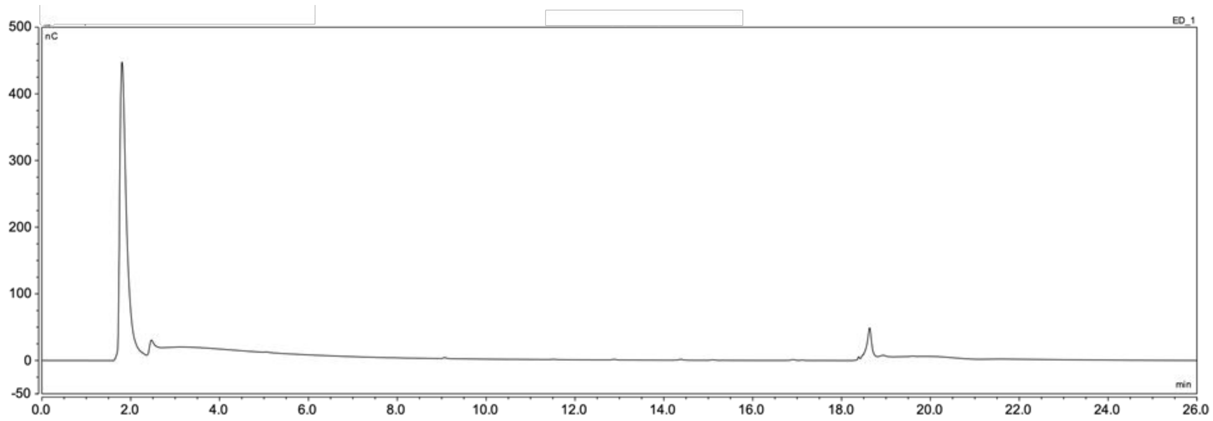

*Hv*EII V1 (A34F, P35A) + (1,3;1,4)-β-D-glucan

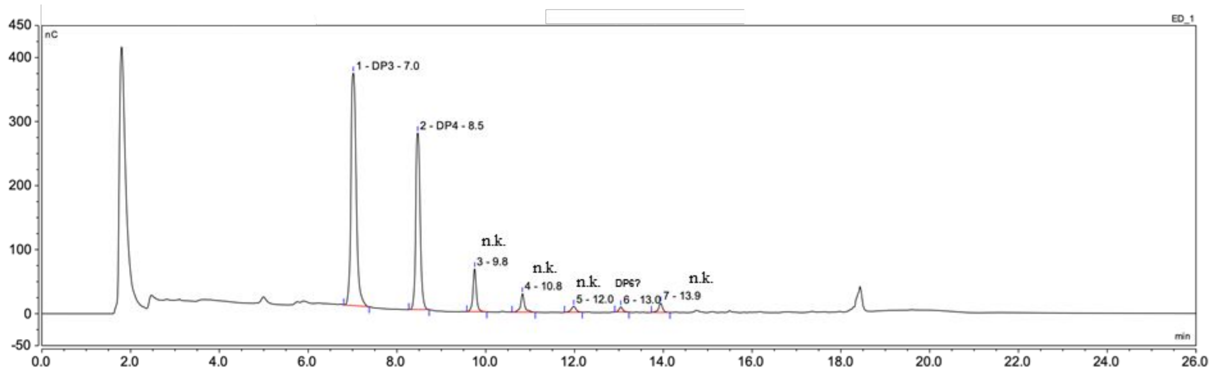

E) *Hv*EII variant V2 (A34F, P35A, N36D, Q37A, A38Q):

*Hv*EII V2 (A34F, P35A, N36D, Q37A, A38Q) + (1,3)-β-D-glucan

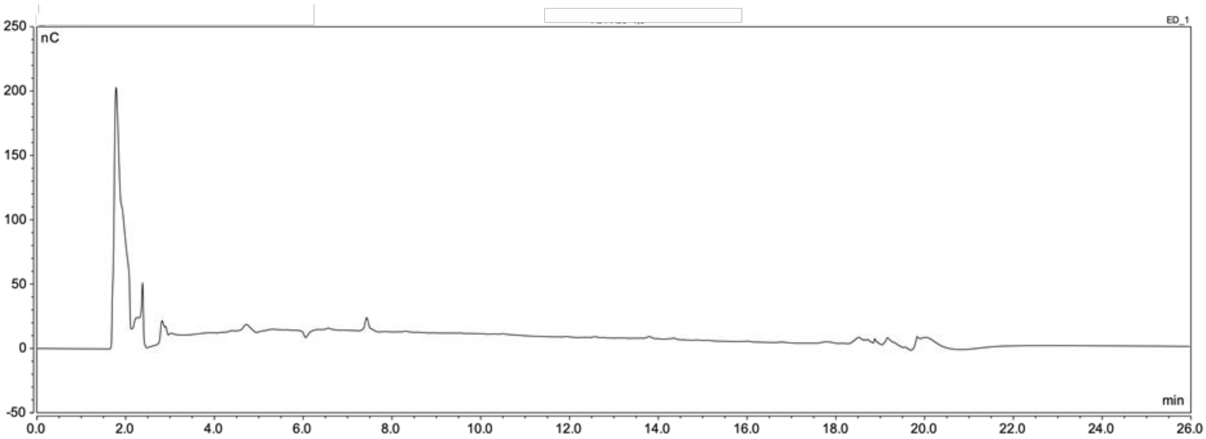

*Hv*EII V2 (A34F, P35A, N36D, Q37A, A38Q) + (1,3;1,4)-β-D-glucan

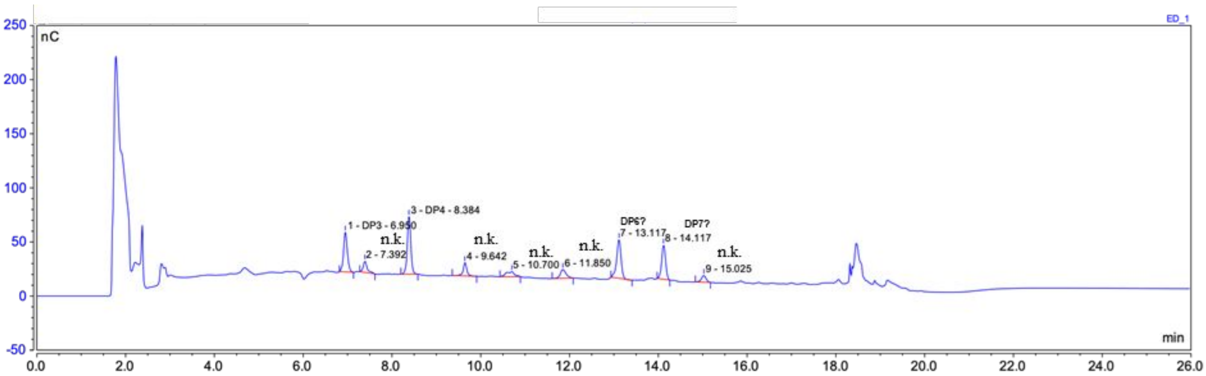

F) *Hv*EII variant V3 (Y177D):

*Hv*EII V3 (Y177D) + (1,3)-β-D-glucan

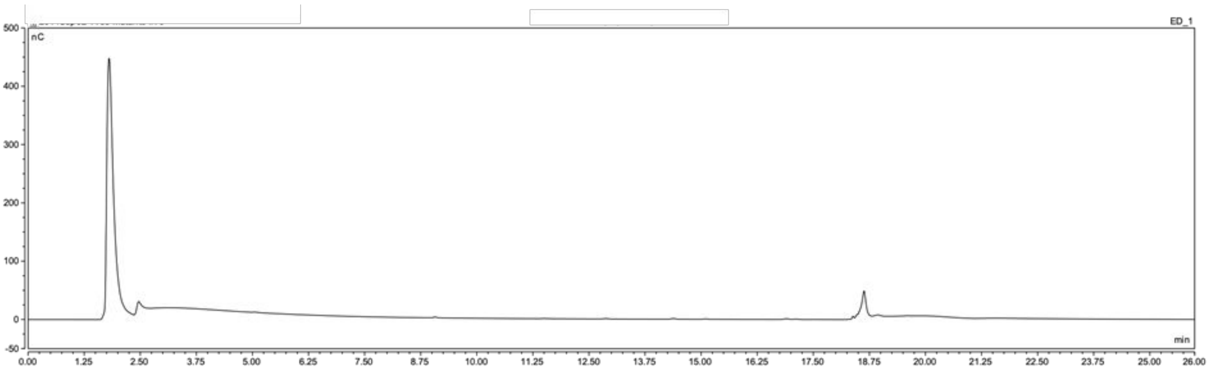

*Hv*EII V3 (Y177D) + (1,3;1,4)-β-D-glucan

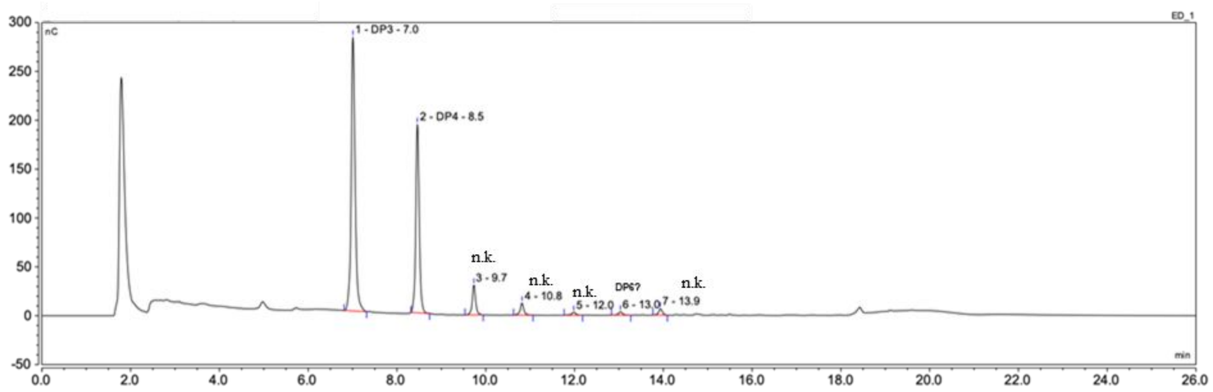

G) *Hv*EII variant V4 (Y177G):

*Hv*EII V4 (Y177G) + (1,3)-β-D-glucan

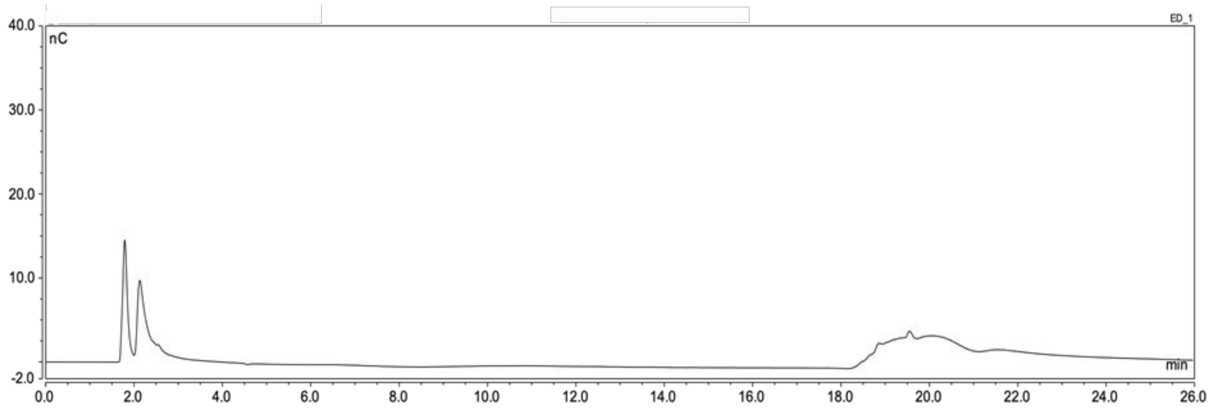

*Hv*EII V4 (Y177G) + (1,3;1,4)-β-D-glucan

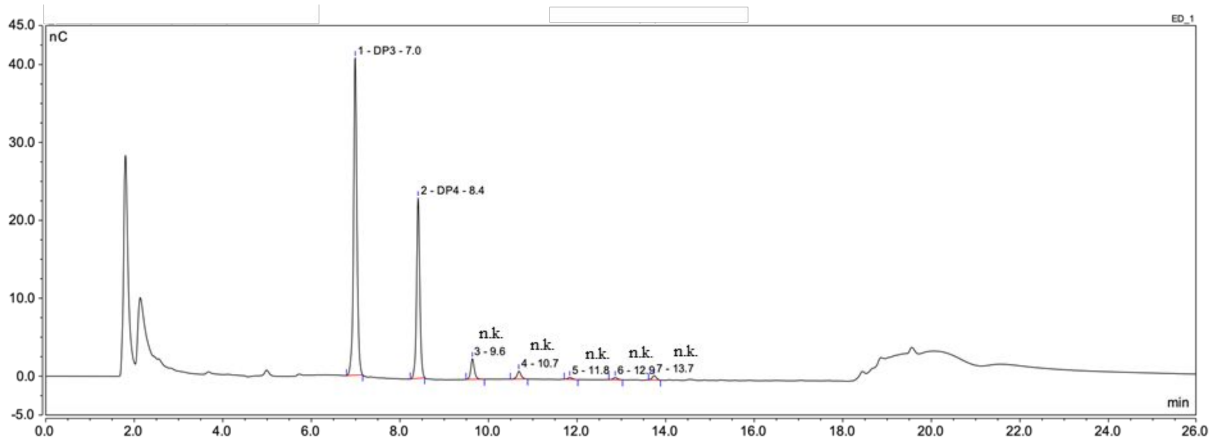

H) *Hv*EII variant V5 (L173F, W175Y, A176R):

*Hv*EII V5 (L173F, W175Y, A176R) + (1,3)-β-D-glucan

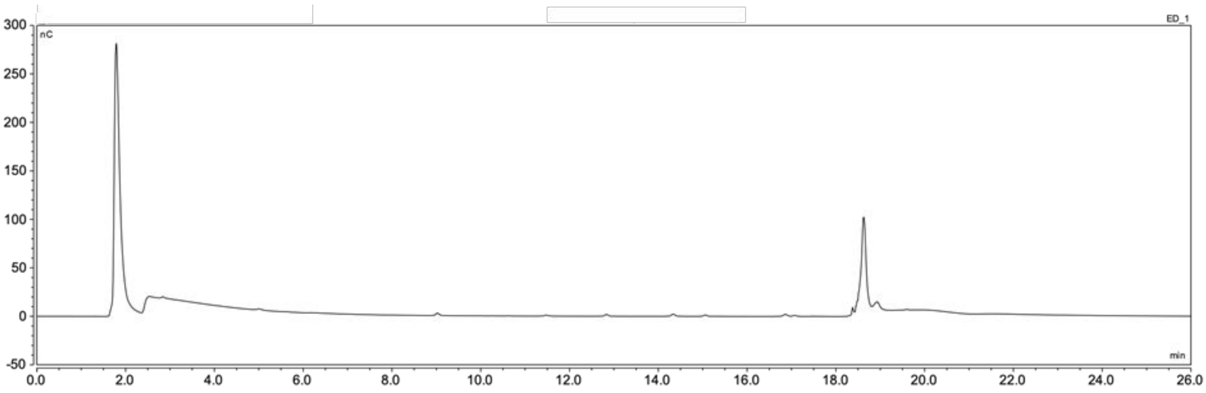

*Hv*EII V5 (L173F, W175Y, A176R) + (1,3;1,4)-β-D-glucan

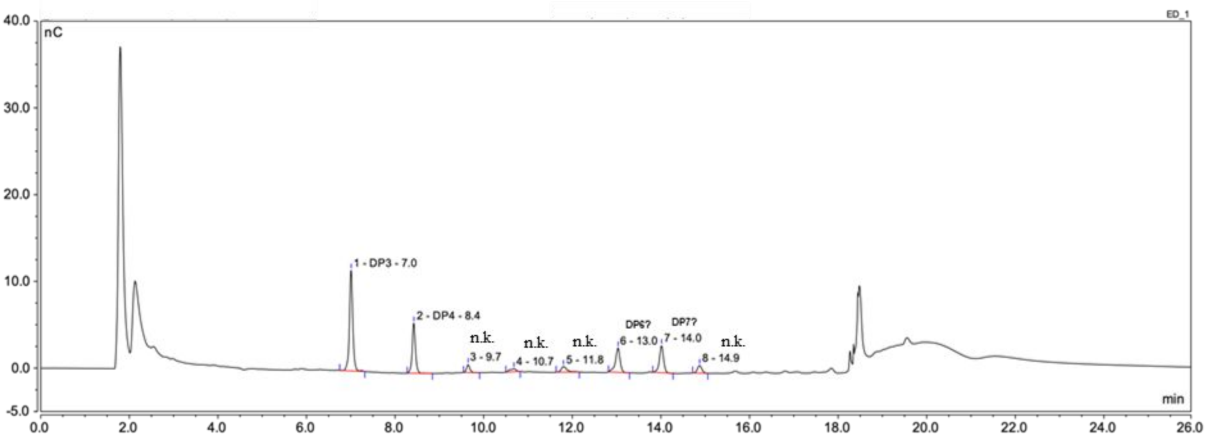

I) *Hv*EII variant V6 (S128R, Q129F):

*Hv*EII V6 (S128R, Q129F) + (1,3)-β-D-glucan

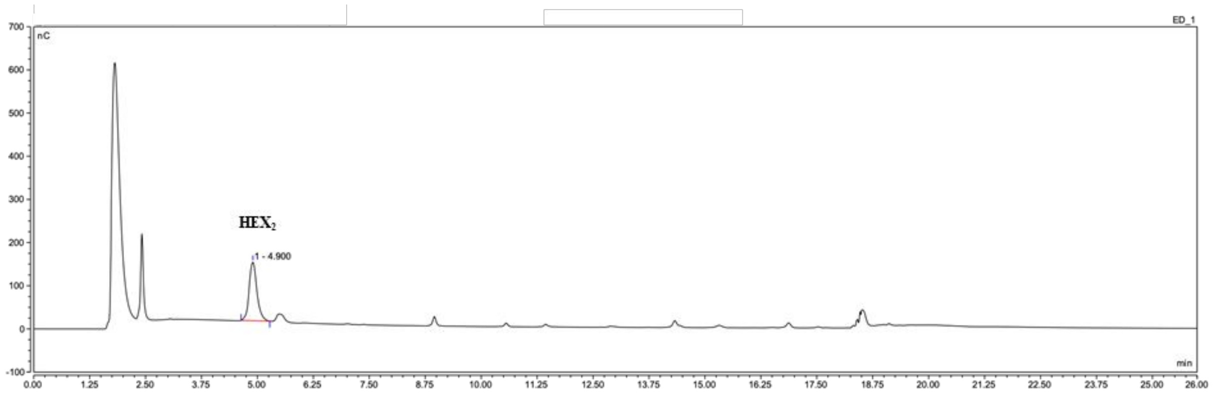

*Hv*EII V6 (S128R, Q129F) + laminarihexose

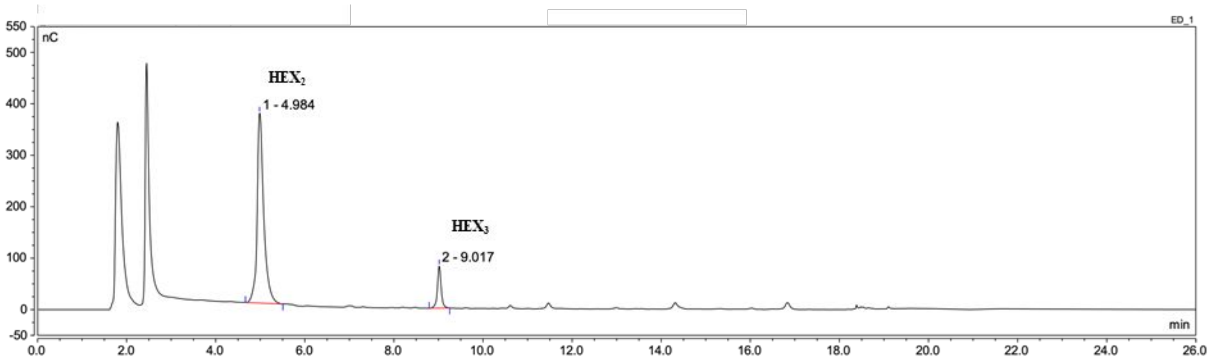

J) *Hv*EII variant V7 (L132V, G133A, V134N, F135S, S136F):

*Hv*EII V7 (L132V, G133A, V134N, F135S, S136F) + (1,3)-β-D-glucan

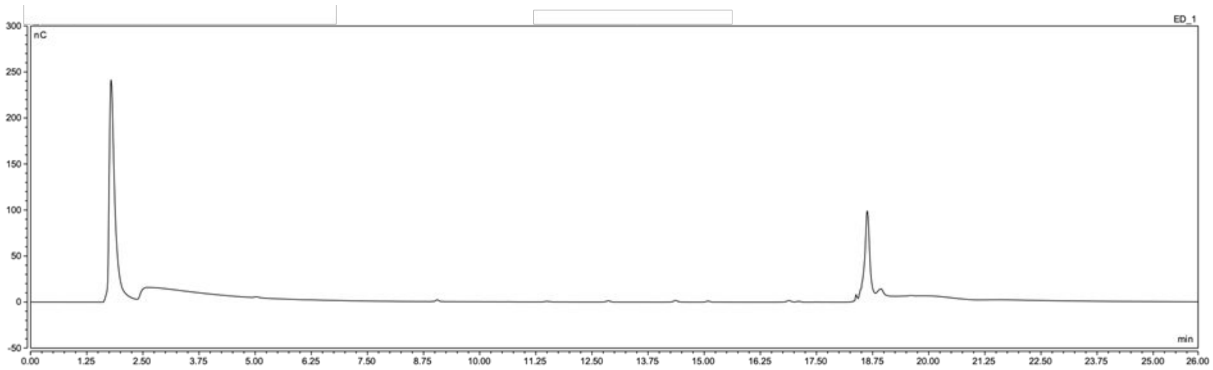

*Hv*EII V7 (L132V, G133A, V134N, F135S, S136F) + (1,3; 1,4)-β-D-glucan

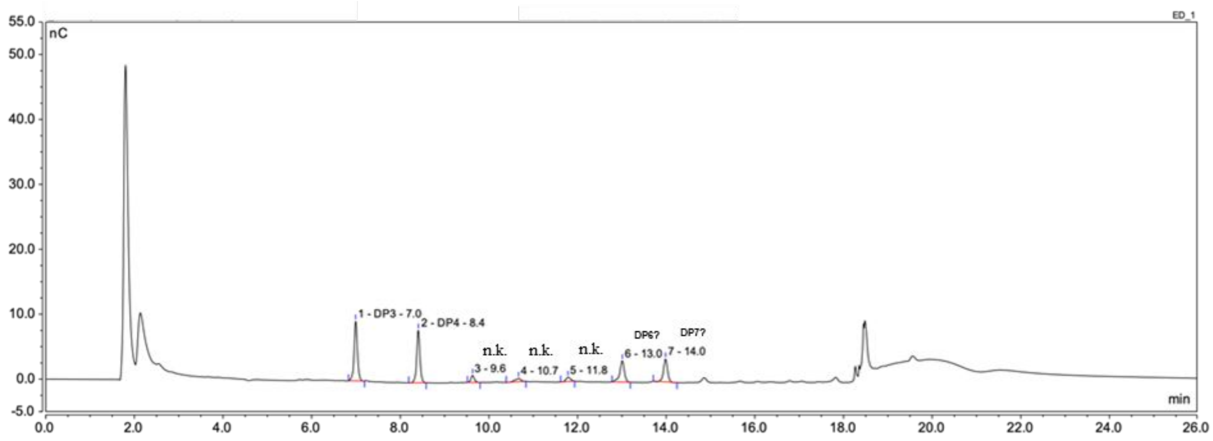

K) *Hv*EII variant V8 (V134N, F135S, S136F):

*Hv*EII V8 (V134N, F135S, S136F) + (1,3)-β-D-glucan

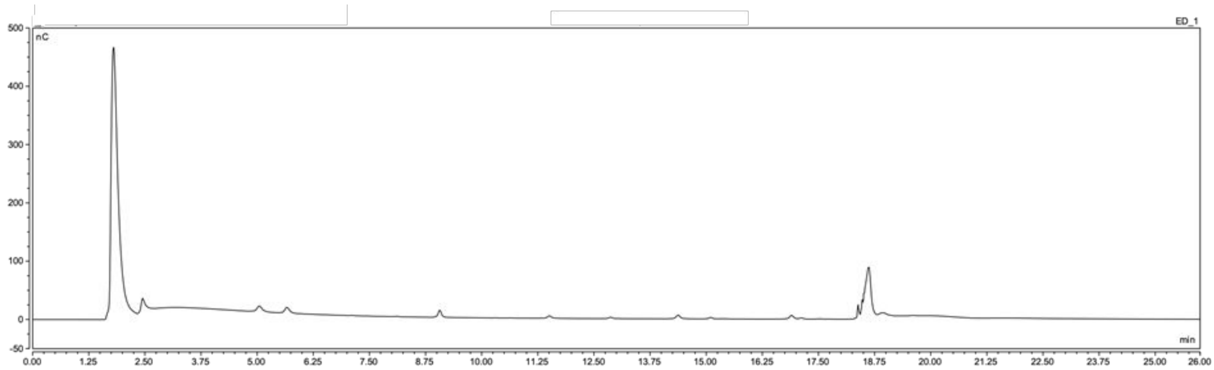

*Hv*EII V8 (V134N, F135S, S136F) + (1,3;1,4)-β-D-glucan

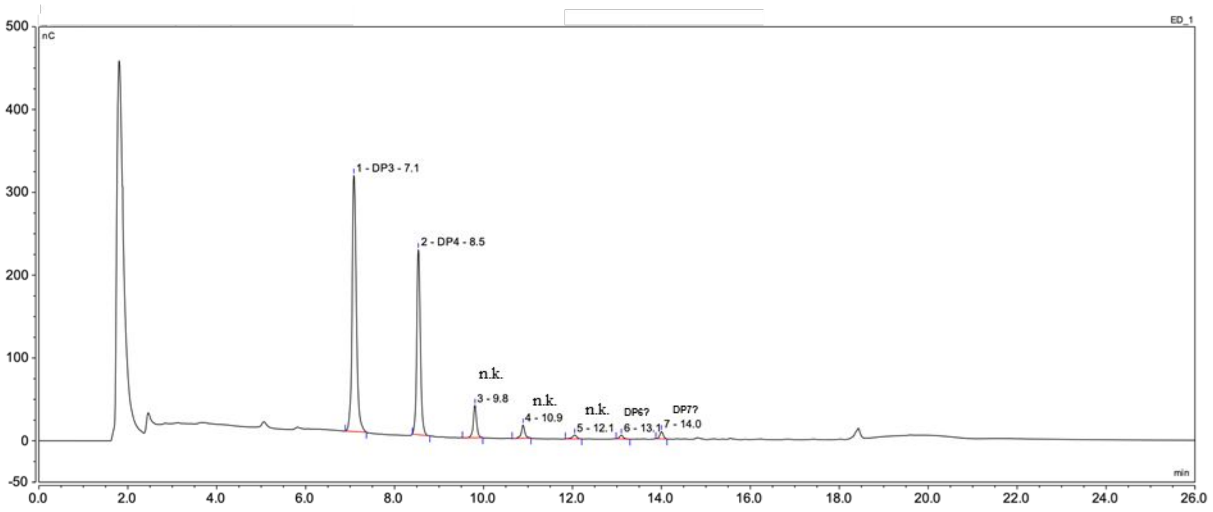

L) *Hv*EII variant V9 (A34F, P35A, Y177D):

*Hv*EII V9 (A34F, P35A, Y177D) + (1,3)-β-D-glucan

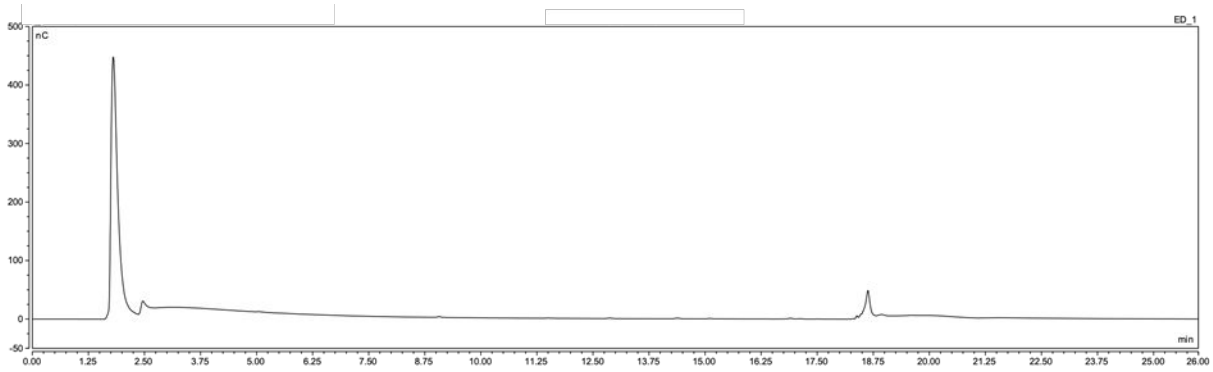

*Hv*EII V9 (A34F, P35A, Y177D) + (1,3;1,4)-β-D-glucan

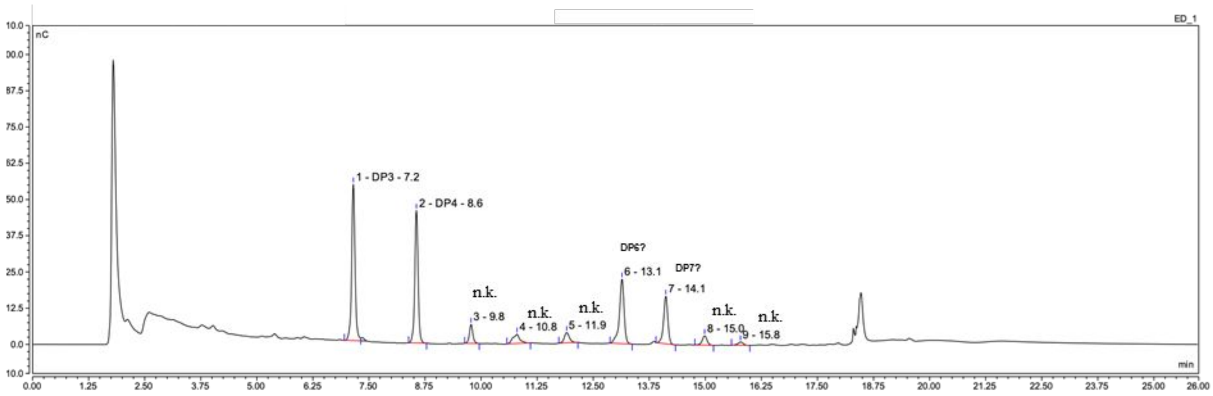

Purified putative DP6

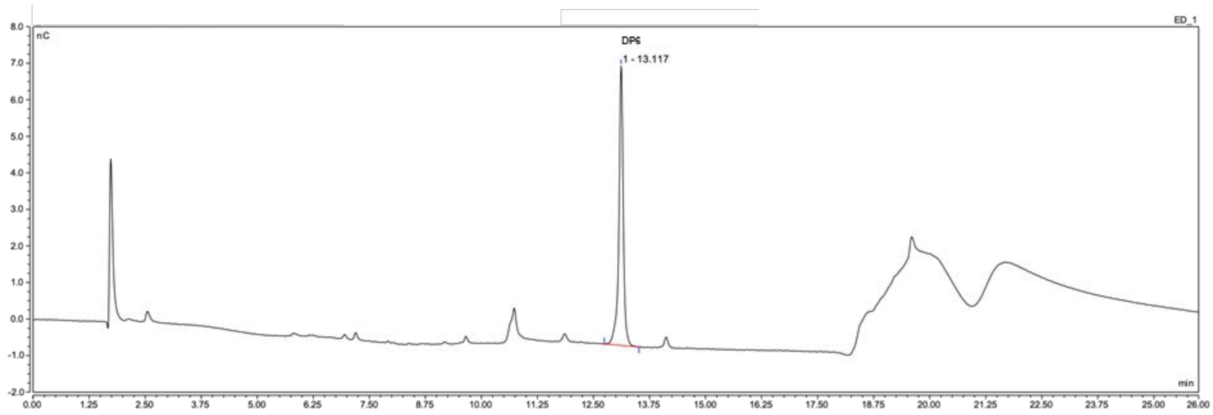

Putative DP6 treated with wild-type *Hv*EII

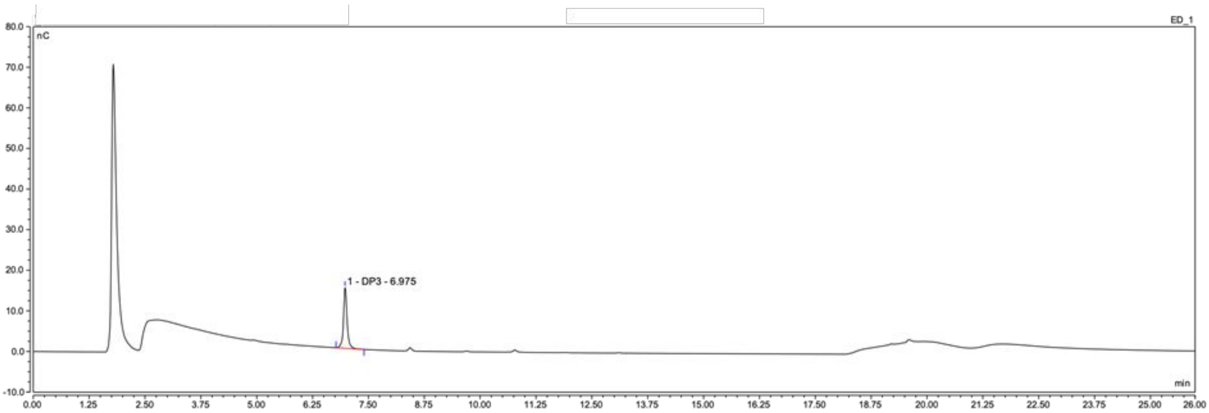

Putative DP6 treated with *Hv*EII variant V9

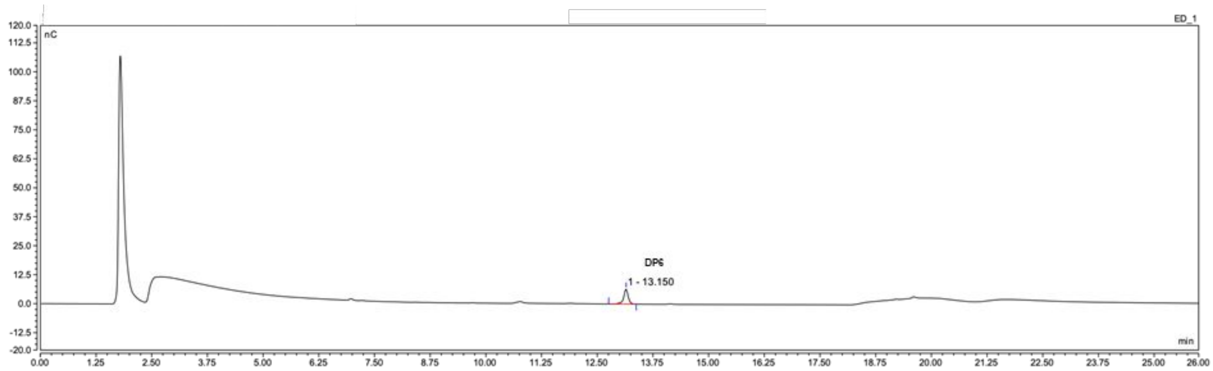

Purified putative DP7

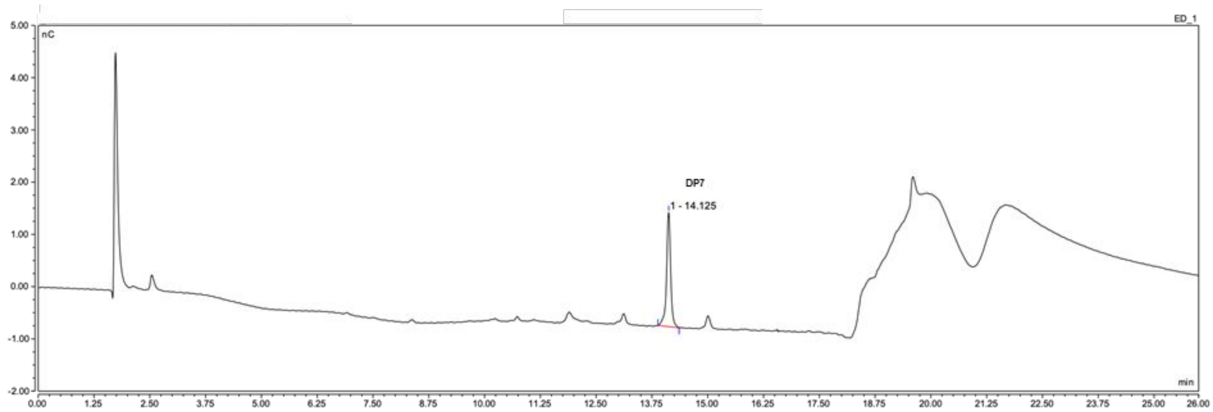

Putative DP7 treated with wild-type *Hv*EII

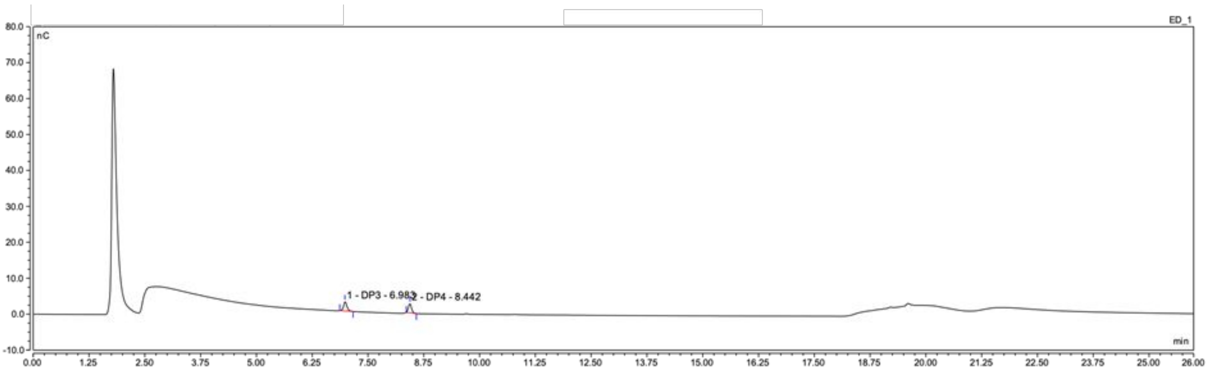

Putative DP7 treated with *Hv*EII variant V9

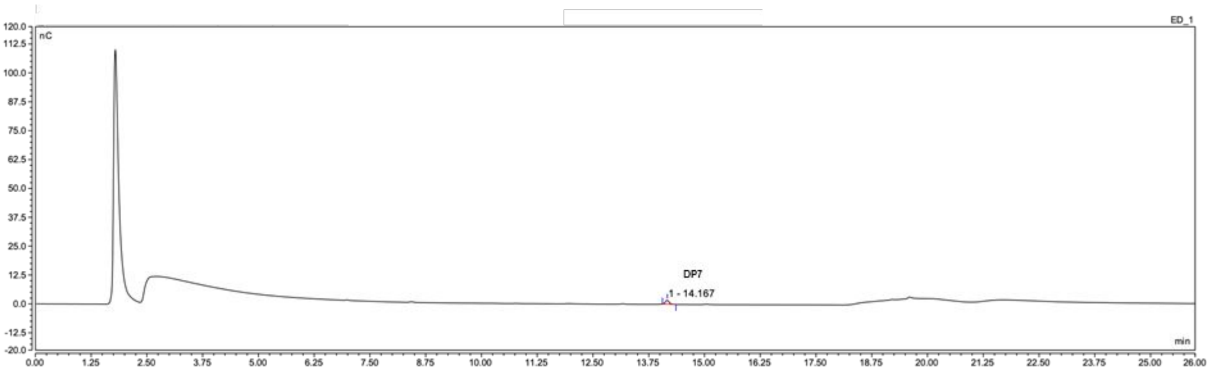

M) *Hv*EII variant V10 (A34F, P35A, S128R, Q129F, Y177D):

*Hv*EII V10 (A34F, P35A, S128R, Q129F, Y177D) + (1,3)-β-D-glucan

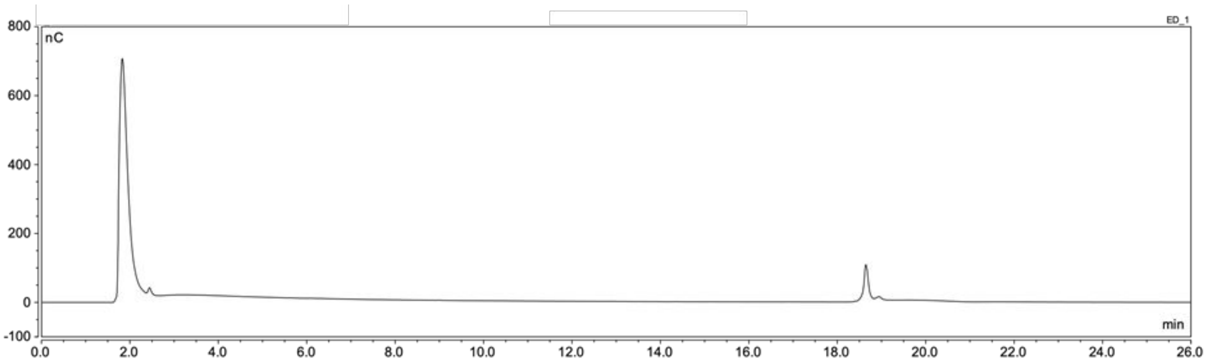

*Hv*EII V10 (A34F+P35A+S128R+Q129F+Y177D) + (1,3;1,4)-β-D-glucan

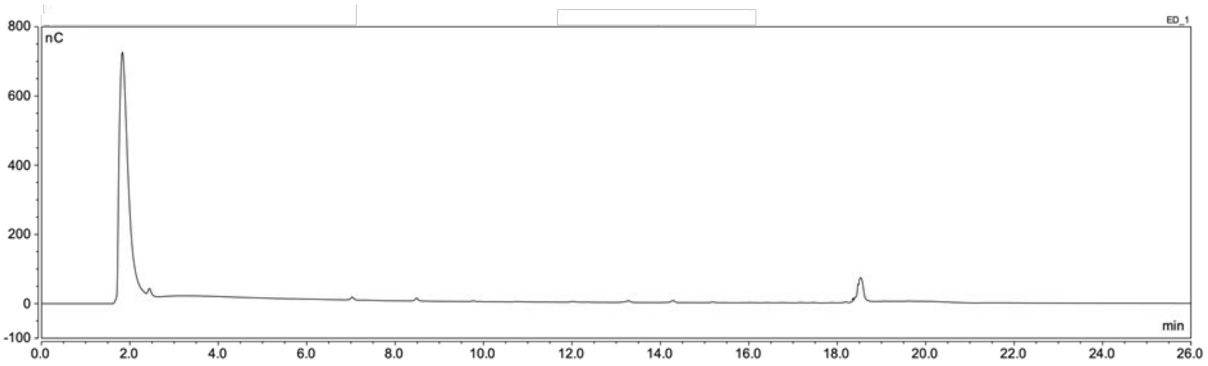

N) *Hv*EII variant V11 (A34F, P35A, N36D, Q37A, A38Q, S128R, Q129F, Y177D):

EII V11 (A34F, P35A, N36D, Q37A, A38Q, S128R, Q129F, Y177D) + (1,3)-β-D-glucan

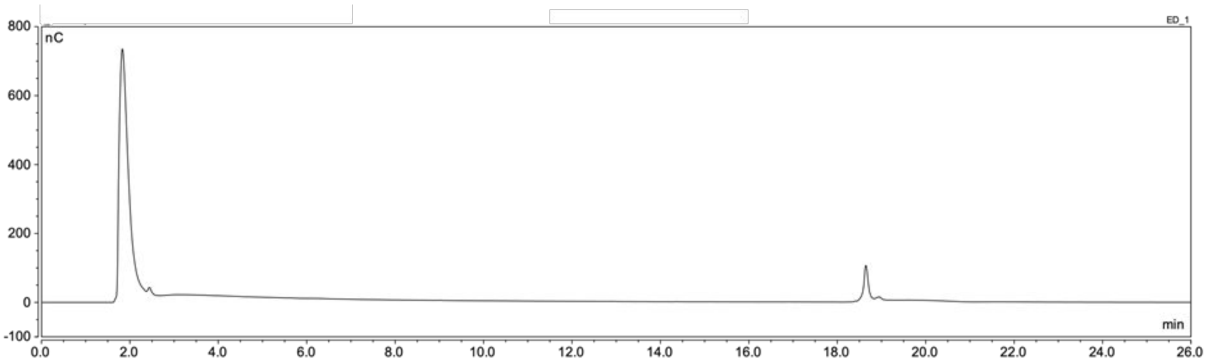

*Hv*EII V11 (A34F, P35A, N36D, Q37A, A38Q, S128R, Q129F, Y177D)

+ (1,3;1,4)-β-D-glucan

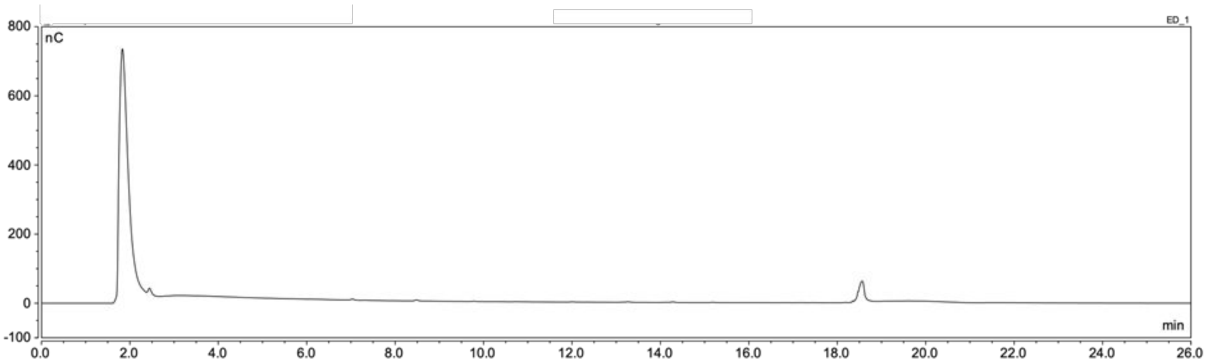

Figure S8: HPAEC analysis of hydrolysis products of (1,3)- and (1,3;1,4)-β-D-glucans treated with WT *Hv*EII and variants. A) Substrate only, B) WT *Hv*EII, C) inactivated mutant *Hv*EII E93A, D) variant V1, E) variant V2, F) variant V3, G) variant V4, H) variant V5, I) variant V6, J) variant V7, K) variant V8, L) variant V9, M) variant V10, N) variant V11. Peaks marked Hex_2 and Hex_3 correspond to laminaribiose and laminaritriose, respectively from the (1,3)-β-D-glucan. Peaks marked DP3, DP4, DP6 and DP7 correspond to oligosaccharides with degrees of polymerizations of 3, 4, 6, and 7 from (1,3;1,4)-β-D-glucan. n.k. = not known.

Supplementary figure S9: Amino acid sequences of WT *Hv*GII and variants generated by mutagenesis

A) Sequence of WT *Hv*GII (yield: about 2.9 mg/L of culture; purity about 94%; specific activity towards the (1,3)-β-D-glucan: 10.9 ± 1.2 U/mg):

IGVCYGVIGNNLPSRSDVVQLYRSKGINGMRIYFADGQALSA
LRNSGIGLILDIGNDQLANIAASTSNAASWVQNNVRPYYPAV
NIKYIAAGNEVQGGATQSILPAMRNLNAALSAAGLGAIKVST
SIRFDEVANSFPPSAGVFKNAYMTDVARLLASTGAPLLANVY
PYFAYRDNPGSISLNYATFQPGTTVRDQNNGLTYTSLFDAMV
DAVYAALEKAGAPAVKVVVSESGWPSAGGFAASAGNARTY
NQGLINHVGGGTPKKREALETYIFAMFNENQKTGDATERSF
GLFNPDKSPAYNIQF

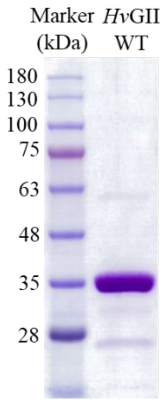

B) Sequence of *Hv*GII E94A:

IGVCYGVIGNNLPSRSDVVQLYRSKGINGMRIYFADGQALSALRNSGIGLILDIGNDQ
LANIAASTSNAASWVQNNVRPYYPAVNIKYIAAGNAVQGGATQSILPAMRNLNAAL
SAAGLGAIKVSTSIRFDEVANSFPPSAGVFKNAYMTDVARLLASTGAPLLANVYPYF
AYRDNPGSISLNYATFQPGTTVRDQNNGLTYTSLFDAMVDAVYAALEKAGAPAVK
VVVSESGWPSAGGFAASAGNARTYNQGLINHVGGGTPKKREALETYIFAMFNENQK
TGDATERSFGLFNPDKSPAYNIQF

C) Sequence of *Hv*GII variant W1 (F34A):

IGVCYGVIGNNLPSRSDVVQLYRSKGINGMRIYAADGQALSALRNSGIGLILDIGNDQ
LANIAASTSNAASWVQNNVRPYYPAVNIKYIAAGNEVQGGATQSILPAMRNLNAAL
SAAGLGAIKVSTSIRFDEVANSFPPSAGVFKNAYMTDVARLLASTGAPLLANVYPYF
AYRDNPGSISLNYATFQPGTTVRDQNNGLTYTSLFDAMVDAVYAALEKAGAPAVK
VVVSESGWPSAGGFAASAGNARTYNQGLINHVGGGTPKKREALETYIFAMFNENQK
TGDATERSFGLFNPDKSPAYNIQF

D) Sequence of *Hv*GII variant W2 (A35P):

IGVCYGVIGNNLPSRSDVVQLYRSKGINGMRIYFPDGQALSALRNSGIGLILDIGNDQ
LANIAASTSNAASWVQNNVRPYYPAVNIKYIAAGNEVQGGATQSILPAMRNLNAAL
SAAGLGAIKVSTSIRFDEVANSFPPSAGVFKNAYMTDVARLLASTGAPLLANVYPYF

AYRDNPGSISLNYATFQPGTTVRDQNNGLTYTSLFDAMVDAVYAALEKAGAPAVK
VVVSESGWPSAGGFAASAGNARTYNQGLINHVGGGTPKKREALETYIFAMFNENQK
TGDATERSFGLFNPDKSPAYNIQF

E) Sequence of *Hv*GII variant W3 (F34A, A35P):
IGVCYGVIGNNLPSRSDVVQLYRSKGINGMRIYAPDGQALSALRNSGIGLILDIGNDQ
LANIAASTSNAASWVQNNVRPYYPAVNIKYIAAGNEVQGGATQSILPAMRNLNAAL
SAAGLGAIKVSTSIRFDEVANSFPPSAGVFKNAYMTDVARLLASTGAPLLANVYPYF
AYRDNPGSISLNYATFQPGTTVRDQNNGLTYTSLFDAMVDAVYAALEKAGAPAVK
VVVSESGWPSAGGFAASAGNARTYNQGLINHVGGGTPKKREALETYIFAMFNENQK
TGDATERSFGLFNPDKSPAYNIQF

F) Sequence of *Hv*GII variant W4 (F34A, A35P, D36N, G37Q, Q38A):
IGVCYGVIGNNLPSRSDVVQLYRSKGINGMRIYAPNQAALSALRNSGIGLILDIGNDQ
LANIAASTSNAASWVQNNVRPYYPAVNIKYIAAGNEVQGGATQSILPAMRNLNAAL
SAAGLGAIKVSTSIRFDEVANSFPPSAGVFKNAYMTDVARLLASTGAPLLANVYPYF
AYRDNPGSISLNYATFQPGTTVRDQNNGLTYTSLFDAMVDAVYAALEKAGAPAVK
VVVSESGWPSAGGFAASAGNARTYNQGLINHVGGGTPKKREALETYIFAMFNENQK
TGDATERSFGLFNPDKSPAYNIQF

G) Sequence of *Hv*GII variant W5 (D175Y; yield: about 0.24 mg/L of culture; purity > 95%; specific activity towards the (1,3)-β-D-glucan: 11.2 ± 0.0 U/mg):
IGVCYGVIGNNLPSRSDVVQLYRSKGINGMRIYFADGQALSALR
NSGIGLILDIGNDQLANIAASTSNAASWVQNNVRPYYPAVNIKY
IAAGNEVQGGATQSILPAMRNLNAALSAAGLGAIKVSTSIRFDE
VANSFPPSAGVFKNAYMTDVARLLASTGAPLLANVYPYFAYRY
NPGSISLNYATFQPGTTVRDQNNGLTYTSLFDAMVDAVYAALE
KAGAPAVKVVVSESGWPSAGGFAASAGNARTYNQGLINHVGG
GTPKKREALETYIFAMFNENQKTGDATERSFGLFNPDKSPAYNIQF

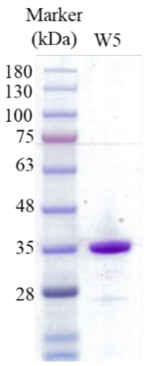

H) Sequence of *Hv*GII variant W6 (F171L, Y173W, R174A, D175Y):
IGVCYGVIGNNLPSRSDVVQLYRSKGINGMRIYFADGQALSALRNSGIGLILDIGNDQ
LANIAASTSNAASWVQNNVRPYYPAVNIKYIAAGNEVQGGATQSILPAMRNLNAAL

SAAGLGAIKVSTSIRFDEVANSFPPSAGVFKNAYMTDVARLLASTGAPLLANVYPYL
AWAYNPGSISLNYATFQPGTTVRDQNNGLTYTSLFDAMVDAVYAALEKAGAPAVK
VVVSESGWPSAGGFAASAGNARTYNQGLINHVGGGTPKKREALETYIFAMFNENQK
TGDATERSFGLFNPDKSPAYNIQF

I) Sequence of *Hv*GII variant W7 (R129S, F130Q):

IGVCYGVIGNNLPSRSDVVQLYRSKGINGMRIYFADGQALSALRNSGIGLILDIGNDQ
LANIAASTSNAASWVQNNVRPYYPAVNIKYIAAGNEVQGGATQSILPAMRNLNAAL
SAAGLGAIKVSTSISQDEVANSFPPSAGVFKNAYMTDVARLLASTGAPLLANVYPYF
AYRDNPGSISLNYATFQPGTTVRDQNNGLTYTSLFDAMVDAVYAALEKAGAPAVK
VVVSESGWPSAGGFAASAGNARTYNQGLINHVGGGTPKKREALETYIFAMFNENQK
TGDATERSFGLFNPDKSPAYNIQF

J) Sequence of *Hv*GII variant W8 (D131A, E132I, V133L, A134G; yield: about 2.1 mg/L of culture; purity > 95%; specific activity towards the (1,3)-β-D-glucan: 1.0 ± 0.1 U/mg):

IGVCYGVIGNNLPSRSDVVQLYRSKGINGMRIYFADGQALSALR
NSGIGLILDIGNDQLANIAASTSNAASWVQNNVRPYYPAVNIKY
IAAGNEVQGGATQSILPAMRNLNAALSAAGLGAIKVSTSIRFAIL
GNSFPPSAGVFKNAYMTDVARLLASTGAPLLANVYPYFAYRDN
PGSISLNYATFQPGTTVRDQNNGLTYTSLFDAMVDAVYAALEK
AGAPAVKVVVSESGWPSAGGFAASAGNARTYNQGLINHVGGG
TPKKREALETYIFAMFNENQKTGDATERSFGLFNPDKSPAYNIQF

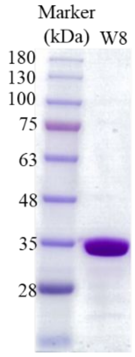

K) Sequence of *Hv*GII variant W9 (S136F, F137S; yield: about 0.5 mg/L of culture; purity > 95%; specific activity towards the (1,3)-β-D-glucan: 7.5 ± 0.2 U/mg):

IGVCYGVIGNNLPSRSDVVQLYRSKGINGMRIYFADGQALSALR
NSGIGLILDIGNDQLANIAASTSNAASWVQNNVRPYYPAVNIKY
IAAGNEVQGGATQSILPAMRNLNAALSAAGLGAIKVSTSIRFDEV
ANFSPPSAGVFKNAYMTDVARLLASTGAPLLANVYPYFAYRDN
PGSISLNYATFQPGTTVRDQNNGLTYTSLFDAMVDAVYAALEK
AGAPAVKVVVSESGWPSAGGFAASAGNARTYNQGLINHVGGG
TPKKREALETYIFAMFNENQKTGDATERSFGLFNPDKSPAYNIQF

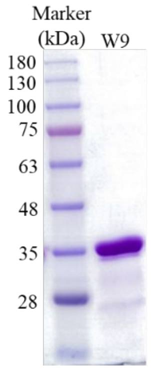

Figure S9: Amino acid sequences of WT *Hv*GII and variants. Amino acid residues substituted with the residues from *Hv*GII are colored black. A) WT *Hv*GII, B) inactivated mutant *Hv*GII E94A, C) variant W1, D) variant W2, E) variant W3, F) variant W4, G) variant W5, H) variant W6, I) variant W7, J) variant W8, K) variant W9. A, G, J and K also show SDS-PAGE gels of the purified proteins to indicate their molecular weights compared with a molecular weight marker ladder.

Supplementary figure S10: Hydrolysis products of WT *Hv*GII and variants generated by mutagenesis analysed by HPAEC

A) Wild-type *Hv*GII:

Wild-type *Hv*GII + (1,3)-β-D-glucan

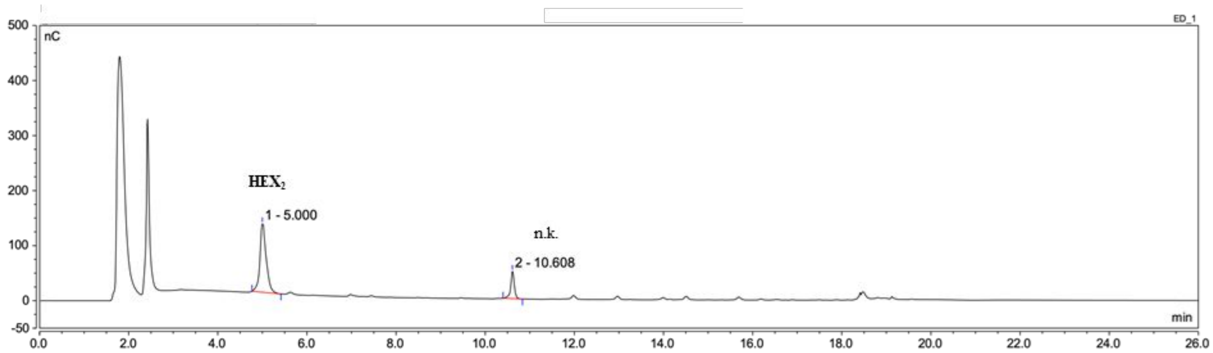

Wild-type *Hv*GII + (1,3;1,4)-β-D-glucan

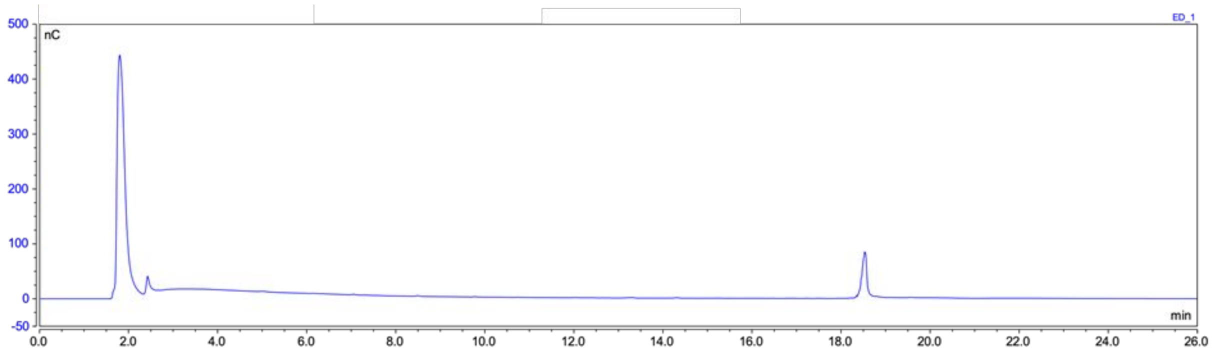

B) *Hv*GII E94A mutant

*Hv*GII mutant E94A + (1,3)-β-D-glucan

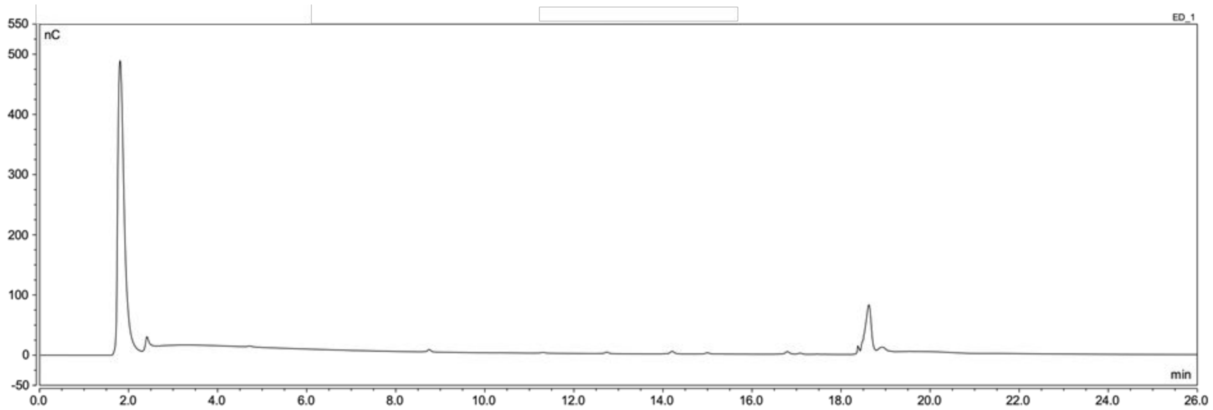

*Hv*GII mutant E94A + (1,3;1,4)-β-D-glucan

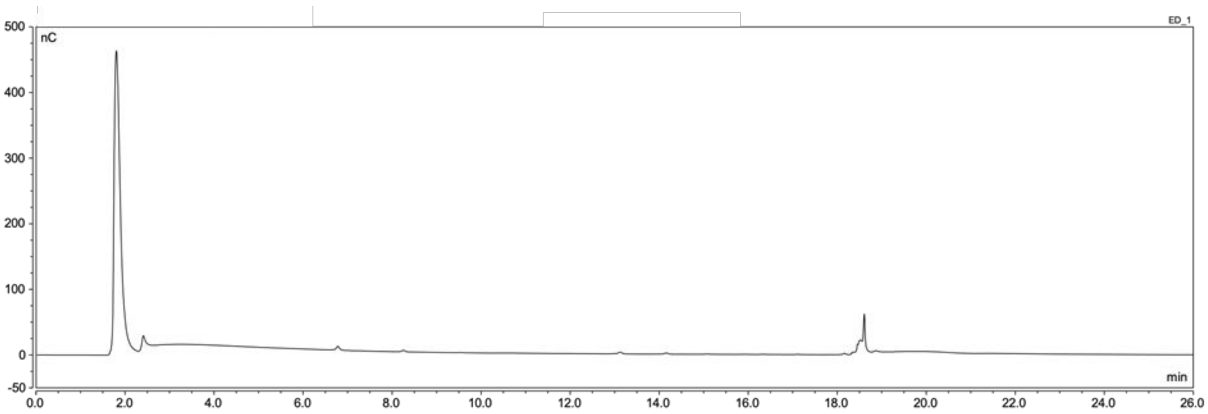

C) *Hv*GII variant W1 (F34A):

*Hv*GII W1 (F34A) + (1,3)-β-D-glucan

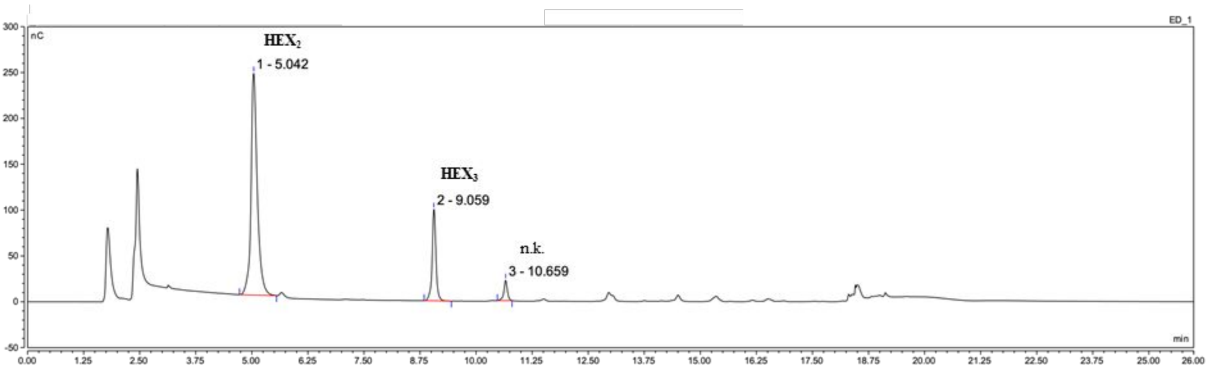

*Hv*GII W1 (F34A) + (1,3;1,4)-β-D-glucan

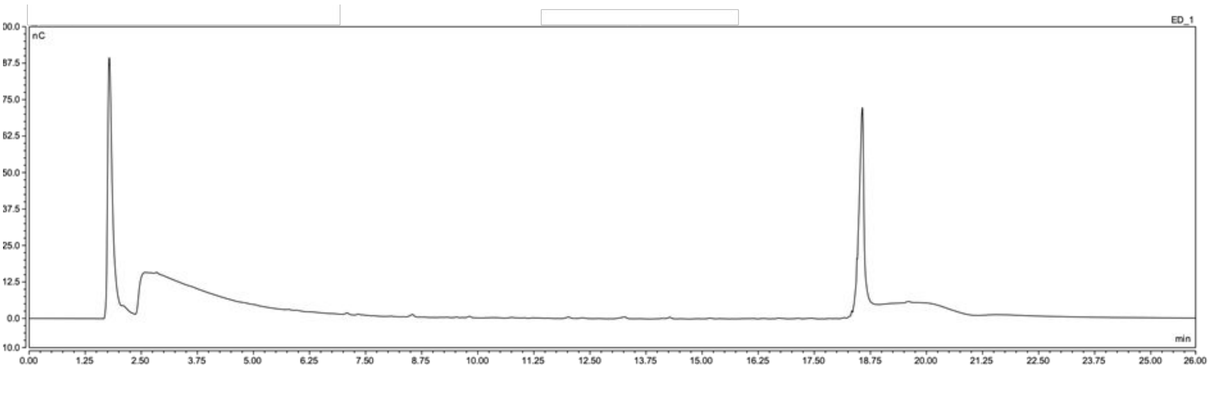

D) *Hv*GII variant W2 (A35P):

*Hv*GII W2 (A35P) + (1,3)-β-D-glucan

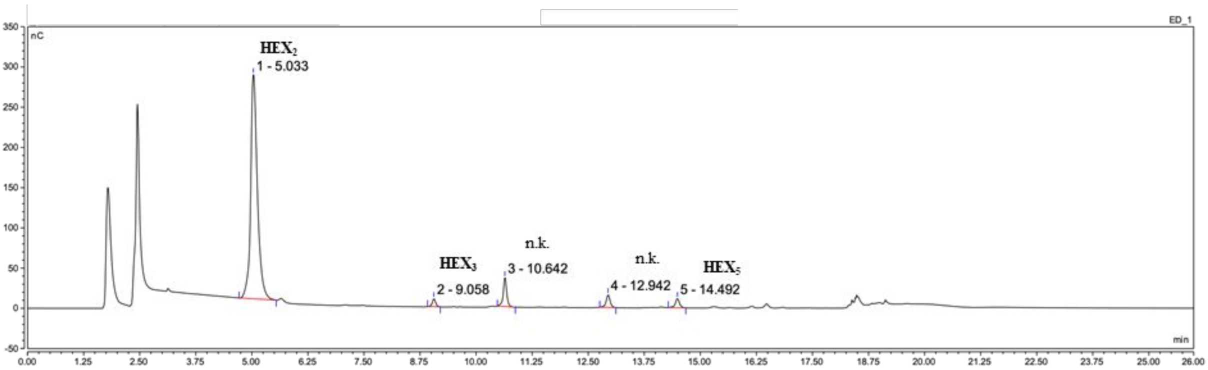

*Hv*GII W2 (A35P) + (1,3;1,4)-β-D-glucan

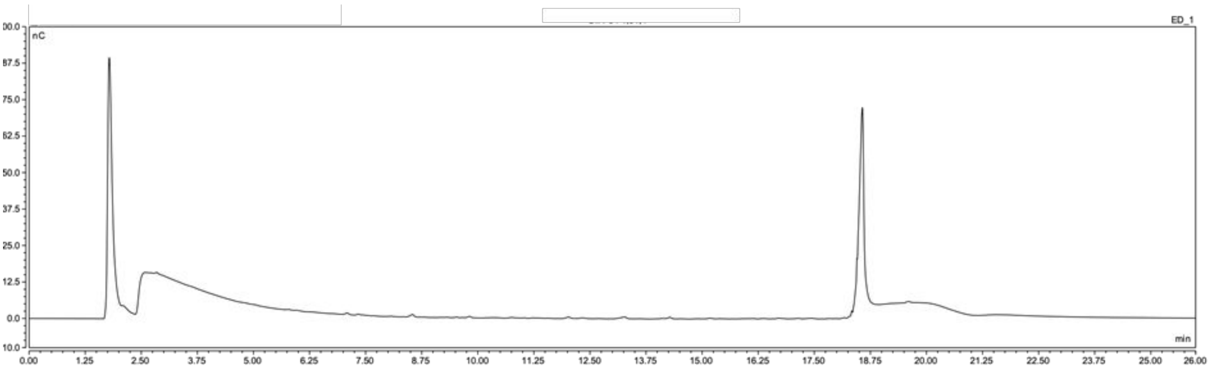

E) *Hv*GII variant W3 (F34A, A35P):

*Hv*GII W3 (F34A, A35P) + (1,3)-β-D-glucan

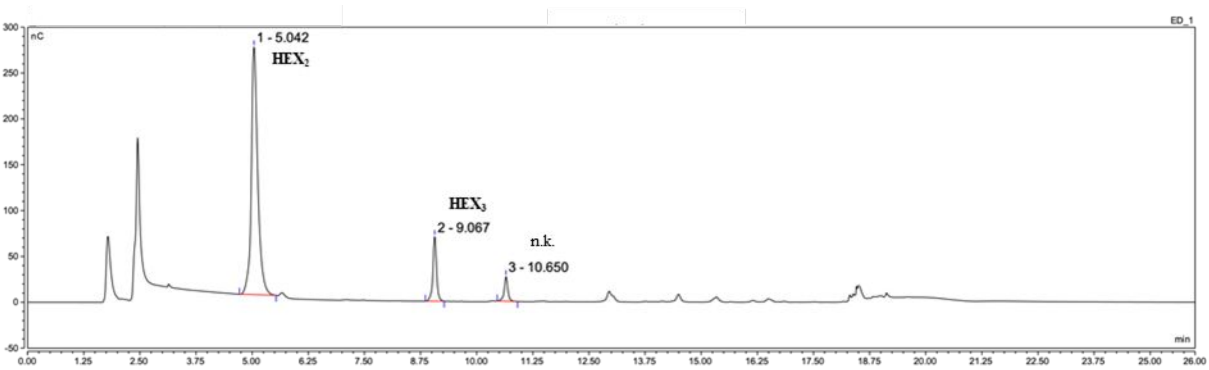

*Hv*GII W3 (F34A, A35P) + (1,3;1,4)-β-D-glucan

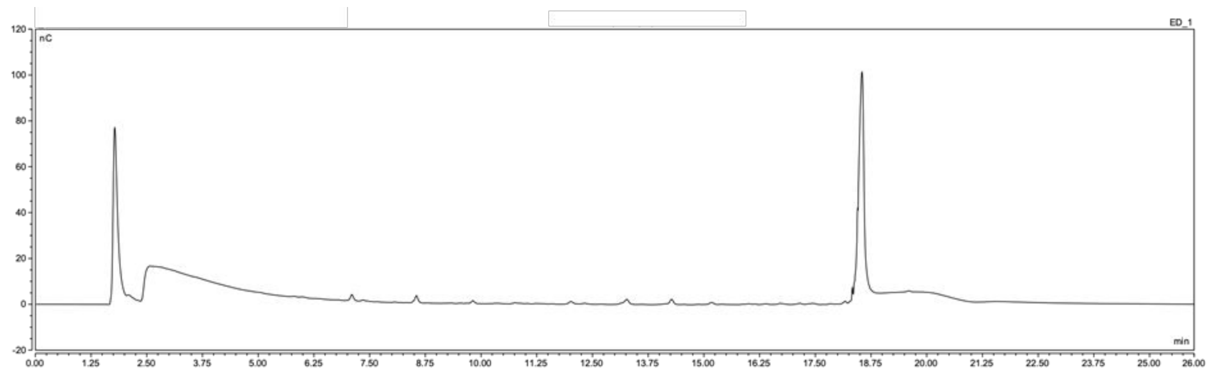

F) *Hv*GII variant W4 (F34A, A35P, D36N, G37Q, Q38A):

*Hv*GII W4 (F34A, A35P, D36N, G37Q, Q38A) + (1,3)-β-D-glucan

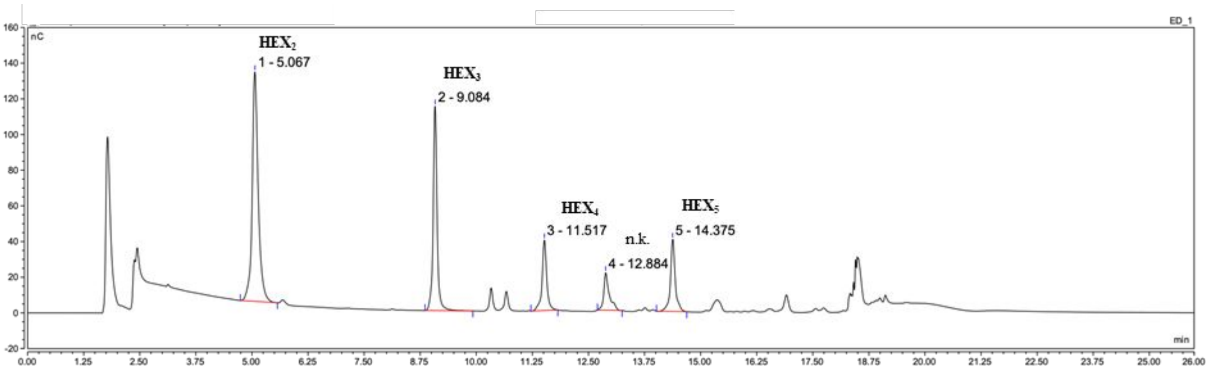

*Hv*GII W4 (F34A, A35P, D36N, G37Q, Q38A) + (1,3;1,4)-β-D-glucan

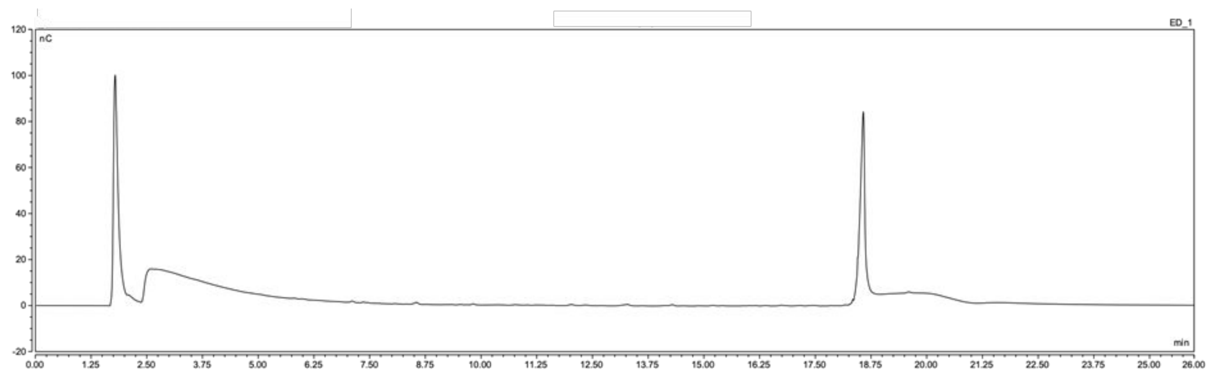

G) *Hv*GII variant W5 (D175Y):

*Hv*GII W5 (D175Y) + (1,3)-β-D-glucan

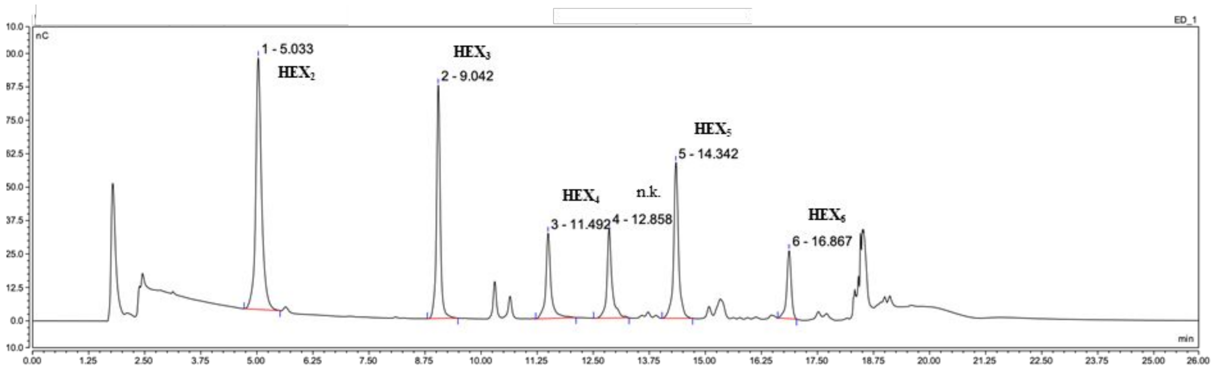

*Hv*GII W5 (D175Y) + (1,3;1,4)-β-D-glucan

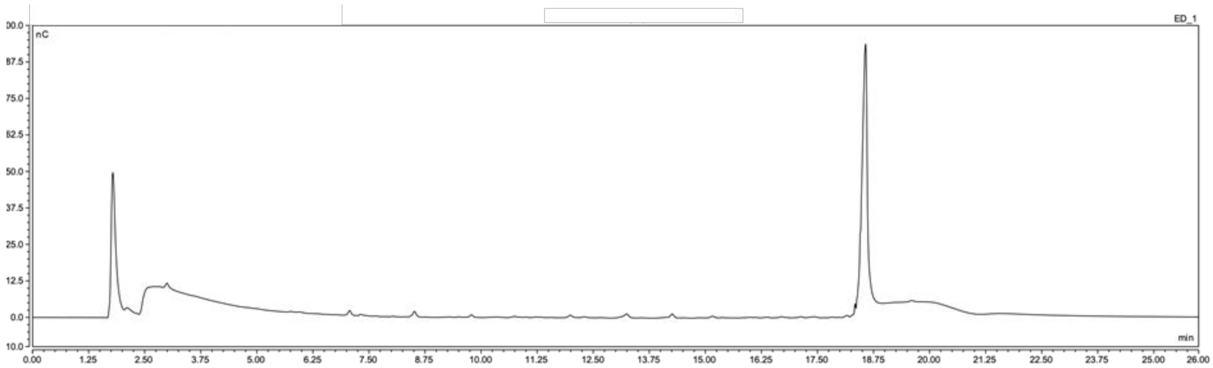

H) *Hv*GII variant W6 (F171L, Y173W, R174A, D175Y):

*Hv*GII W6 (F171L, Y173W, R174A, D175Y) + (1,3)-β-D-glucan

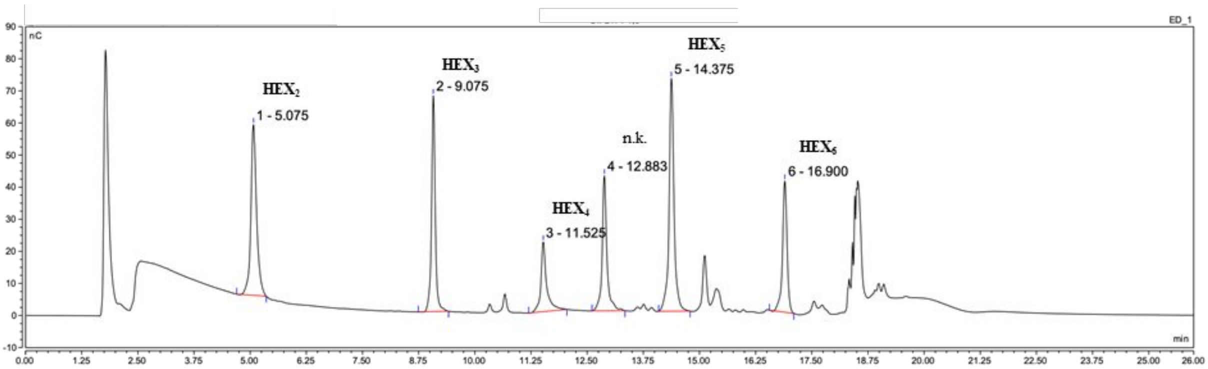

*Hv*GII W6 (F171L, Y173W, R174A, D175Y) + (1,3;1,4)-β-D-glucan

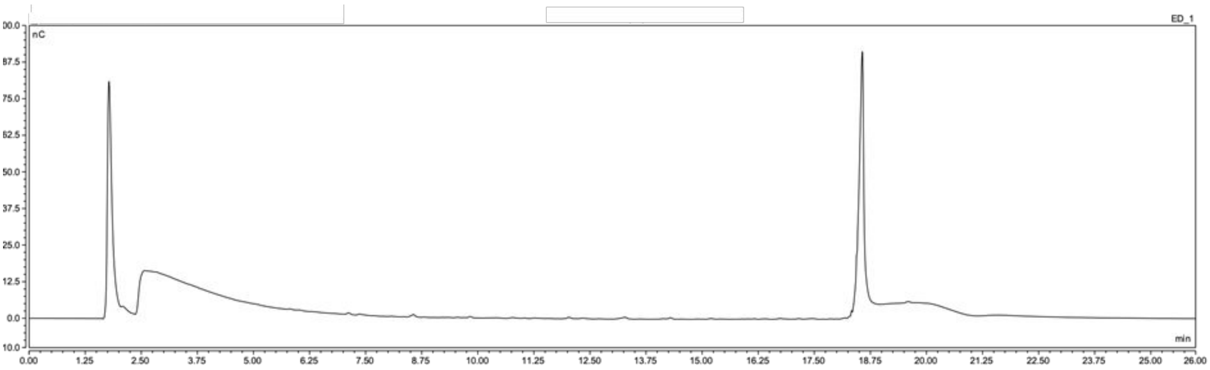

I) *Hv*GII variant W7 (R129S, F130Q):

*Hv*GII W7 (R129S, F130Q) + (1,3)-β-D-glucan

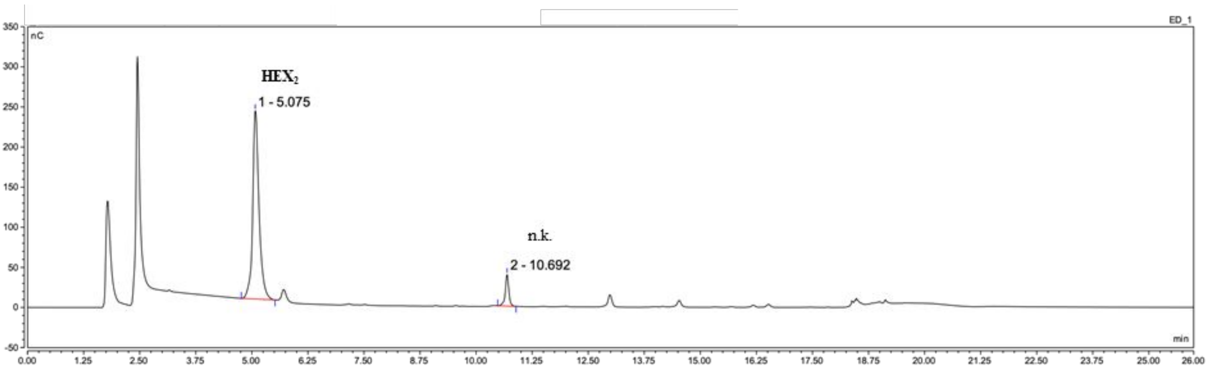

*Hv*GII W7 (R129S, F130Q) + (1,3;1,4)-β-D-glucan

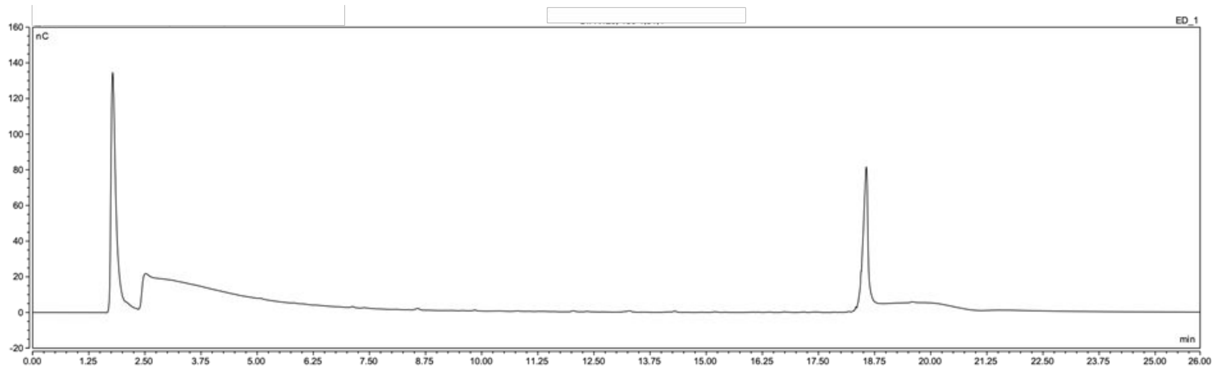

J) *Hv*GII variant W8 (D131A, E132I, V133L, A134G):

*Hv*GII W8 (D131A, E132I, V133L, A134G) + (1,3)-β-D-glucan

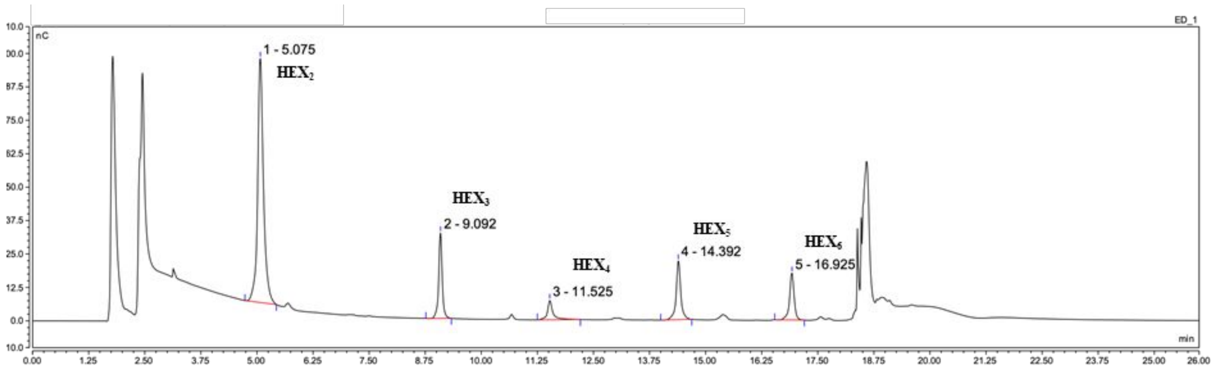

*Hv*GII W8 (D131A, E132I, V133L, A134G) + (1,3;1,4)-β-D-glucan

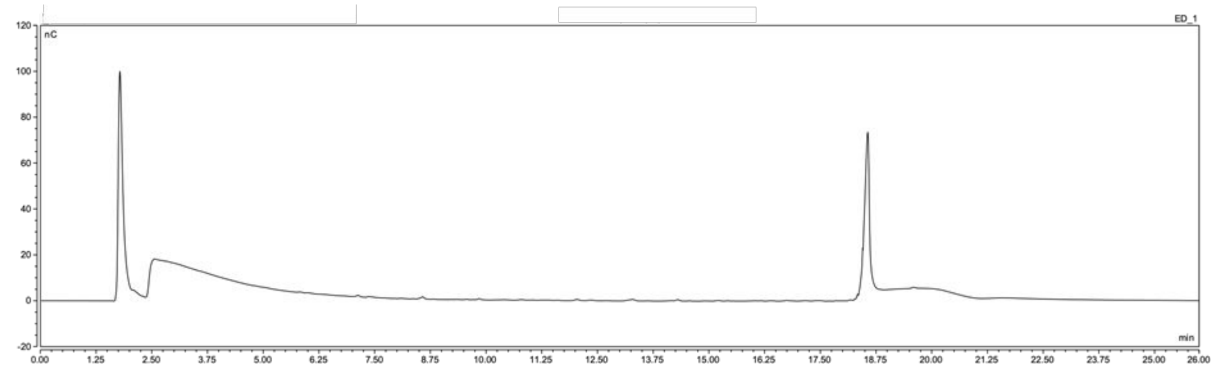

K) *Hv*GII variant W9 (S136F, F137S):

*Hv*GII W9 (S136F, F137S) + (1,3)-β-D-glucan

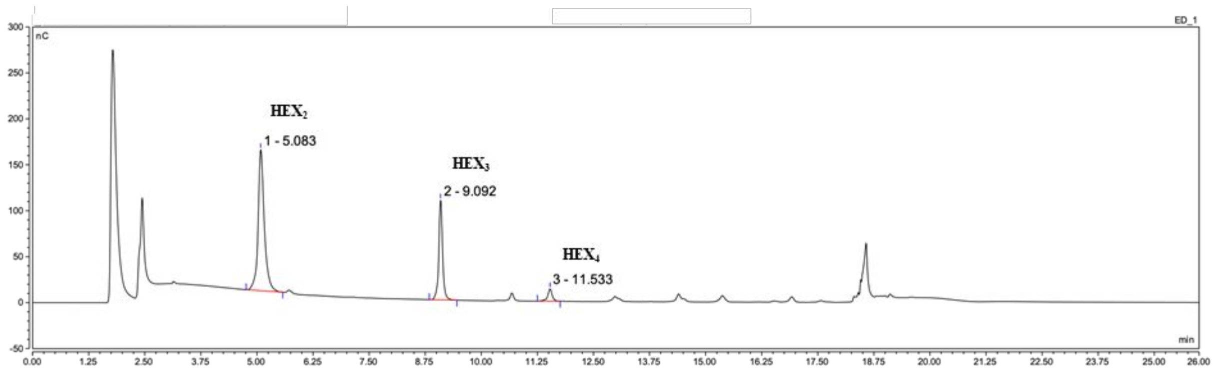

*Hv*GII W9 (S136F, F137S) + (1,3;1,4)-β-D-glucan

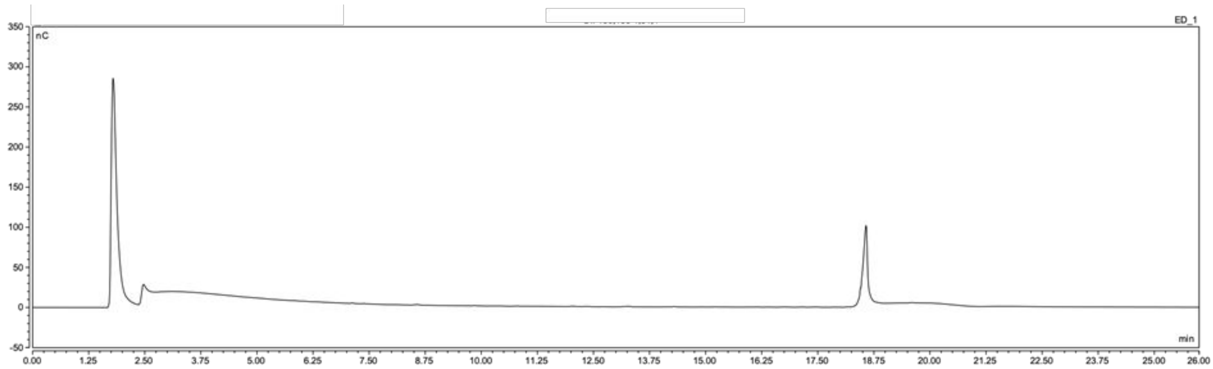

Figure S10: HPAEC analysis of hydrolysis products of (1,3)- and (1,3;1,4)-β-D-glucans treated with WT *Hv*GII and variants. A) WT *Hv*GII, B) inactivated mutant *Hv*GII E94A, C) variant W1, D) variant W2, E) variant W3, F) variant W4, G) variant W5, H) variant W6, I) variant W7, J) variant W8, K) variant W9. Peaks marked Hex_2, Hex_3, Hex_4 Hex_5 and Hex_6 correspond to laminaribiose, laminaritriose, laminaritetraose, laminaripentaose and laminarihexaose, respectively from the (1,3)-β-D-glucan. n.k. = not known.

Supplementary figure S11: Amino acid sequences of *Hv*EII and *Hv*GII hybrid variants

A) Sequence of *Hv*EII variant ES2 with sequence change from 61 to 120 (*Hv*GII sequence in red):

IGVCYGMSANNLPAASTVVSMFKFNGIKSMRLYAPNQAALQAVGGTGINVVVGAP
NDVLSNIAASTSNAASWVQNNVRPYYPAVNIKYIAAGNEVQGGATQSILPAMRNLN
AALSAAGLGHIKVTTSVSQAILGVFSPPSAGSFTGEAAAFMGPVVQFLARTNAPLMA
NIYPYLAWAYNPSAMDMGYALFNASGTVVRDGAYGYQNLFDTTVDAFYTAMGKH
GGSSVKLVVSESGWPSGGGTAATPANARFYNQHLINHVGRGTPRHPGAIETYIFAMF
NENQKDSGVEQNWGLFYPNMQHVYPINF

B) Sequence of *Hv*EII variant ES3 with sequence change from 120 to 179 (*Hv*GII sequence in red):

IGVCYGMSANNLPAASTVVSMFKFNGIKSMRLYAPNQAALQAVGGTGINVVVGAP
NDVLSNLAASPAAAASWVKSNIQAYPKVSFRYVCVGNEVAGGATRNLVPAMKNVH
GALVAAGLGAIKVSTSIRFDEVANSFPPSAGVFKNAYMTDVARLLASTGAPLLANVY
PYFAYRDNPSAMDMGYALFNASGTVVRDGAYGYQNLFDTTVDAFYTAMGKHGGS
SVKLVVSESGWPSGGGTAATPANARFYNQHLINHVGRGTPRHPGAIETYIFAMFNEN
QKDSGVEQNWGLFYPNMQHVYPINF

C) Sequence of *Hv*EII variant ES4 with sequence change from 180 to 237 (*Hv*GII sequence in red; purity > 95%; specific activity towards the (1,3;1,4)-β-D-glucan: 7.7 ± 0.4 U/mg):

IGVCYGMSANNLPAASTVVSMFKFNGIKSMRLYAPNQAALQAV
GGTGINVVVGAPNDVLSNLAASPAAAASWVKSNIQAYPKVSFR
YVCVGNEVAGGATRNLVPAMKNVHGALVAAGLGHIKVTTSVS
QAILGVFSPPSAGSFTGEAAAFMGPVVQFLARTNAPLMANIYPY
LAWAYNPGSISLNYATFQPGTTVRDQNNGLTYTSLFDAMVDAV
YAALEKAGAPAVKVVVSESGWPSGGGTAATPANARFYNQHLIN
HVGRGTPRHPGAIETYIFAMFNENQKDSGVEQNWGLFYPNMQH
VYPINF

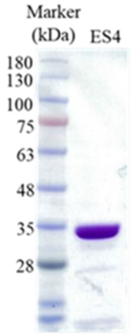

D) Sequence of *Hv*EII variant ES5 with sequence change from 238 to 307 (*Hv*GII sequence in red):

IGVCYGMSANNLPAASTVVSMFKFNGIKSMRLYAPNQAALQAVGGTGINVVVGAP
NDVLSNLAASPAAAASWVKSNIQAYPKVSFRYVCVGNEVAGGATRNLVPAMKNVH
GALVAAGLGHIKVTTSVSQAILGVFSPPSAGSFTGEAAAFMGPVVQFLARTNAPLMA
NIYPYLAWAYNPSAMDMGYALFNASGTVVRDGAYGYQNLFDTTVDAFYTAMGKH
GGSSVKLVVSESGWPSAGGFAASAGNARTYNQGLINHVGGGTPKKREALETYIFAM
FNENQKTGDATERSFGLFNPDKSPAYNIQ

E) Sequence of *Hv*GII variant GS1 with sequence change from 1 to 60 (*Hv*GII sequence in red):

IGVCYGMSANNLPAASTVVSMFKFNGIKSMRLYAPNQAALQAVGGTGINVVVGAP
NDVLSNIAASTSNAASWVQNNVRPYYPAVNIKYIAAGNEVQGGATQSILPAMRNLN
AALSAAGLGAIKVSTSIRFDEVANSFPPSAGVFKNAYMTDVARLLASTGAPLLANVY
PYFAYRDNPGSISLNYATFQPGTTVRDQNNGLTYTSLFDAMVDAVYAALEKAGAPA
VKVVVSESGWPSAGGFAASAGNARTYNQGLINHVGGGTPKKREALETYIFAMFNEN
QKTGDATERSFGLFNPDKSPAYNIQF

F) Sequence of *Hv*GII variant GS2 with sequence change from 61 to 120 (*Hv*GII sequence in red):

IGVCYGVIGNNLPSRSDVVQLYRSKGINGMRIYFADGQALSALRNSGIGLILDIGNDQ
LANLAASPAAAASWVKSNIQAYPKVSFRYVCVGNEVAGGATRNLVPAMKNVHGAL
VAAGLGAIKVSTSIRFDEVANSFPPSAGVFKNAYMTDVARLLASTGAPLLANVYPYF
AYRDNPGSISLNYATFQPGTTVRDQNNGLTYTSLFDAMVDAVYAALEKAGAPAVK
VVVSESGWPSAGGFAASAGNARTYNQGLINHVGGGTPKKREALETYIFAMFNENQK
TGDATERSFGLFNPDKSPAYNIQF

G) Sequence of *Hv*GII variant GS3 with sequence change from 121 to 177 (*Hv*GII sequence in red):

IGVCYGVIGNNLPSRSDVVQLYRSKGINGMRIYFADGQALSALRNSGIGLILDIGNDQ
LANIAASTSNAASWVQNNVRPYYPAVNIKYIAAGNEVQGGATQSILPAMRNLNAAL
SAAGLGHIKVTTSVSQAILGVFSPPSAGSFTGEAAAFMGPVVQFLARTNAPLMANIYP
YLAWAYNPGSISLNYATFQPGTTVRDQNNGLTYTSLFDAMVDAVYAALEKAGAPA
VKVVVSESGWPSAGGFAASAGNARTYNQGLINHVGGGTPKKREALETYIFAMFNEN
QKTGDATERSFGLFNPDKSPAYNIQF

H) The sequence of *Hv*GII variant GS4 with sequence change from 178 to 235 (*Hv*GII sequence in red):

IGVCYGVIGNNLPSRSDVVQLYRSKGINGMRIYFADGQALSALRNSGIGLILDIGNDQ
LANIAASTSNAASWVQNNVRPYYPAVNIKYIAAGNEVQGGATQSILPAMRNLNAAL
SAAGLGAIKVSTSIRFDEVANSFPPSAGVFKNAYMTDVARLLASTGAPLLANVYPYF
AYRDNPSAMDMGYALFNASGTVVRDGAYGYQNLFDTTVDAFYTAMGKHGGSSVK
LVVSESGWPSAGGFAASAGNARTYNQGLINHVGGGTPKKREALETYIFAMFNENQK
TGDATERSFGLFNPDKSPAYNIQF

I) The sequence of *Hv*GII variant GS5 with sequence change from 236 to 305 (*Hv*GII sequence in red):

IGVCYGVIGNNLPSRSDVVQLYRSKGINGMRIYFADGQALSALRNSGIGLILDIGNDQ
LANIAASTSNAASWVQNNVRPYYPAVNIKYIAAGNEVQGGATQSILPAMRNLNAAL
SAAGLGAIKVSTSIRFDEVANSFPPSAGVFKNAYMTDVARLLASTGAPLLANVYPYF
AYRDNPGSISLNYATFQPGTTVRDQNNGLTYTSLFDAMVDAVYAALEKAGAPAVK
VVVSESGWPSGGGTAATPANARFYNQHLINHVGRGTPRHPGAIETYIFAMFNENQK
DSGVEQNWGLFYPNMQHVYPINF

Figure S11: Amino acid sequences of *Hv*EII and *Hv*GII hybrid variants. These variants are represented schematically in Figure 7B in the main text. Amino acid residues from *Hv*EII and *Hv*GII are colored black and red, respectively. A) ES2, B) ES3, C) ES4, D) ES5, E) GS1, F) GS2, G) GS3, H) GS4, I) GS5.

Supplementary figure S12: Hydrolysis products of *Hv*EII and *Hv*GII hybrid variants analysed by HPAEC

A) *Hv*EII variant ES2:

*Hv*EII variant ES2 + (1,3)-β-D-glucan

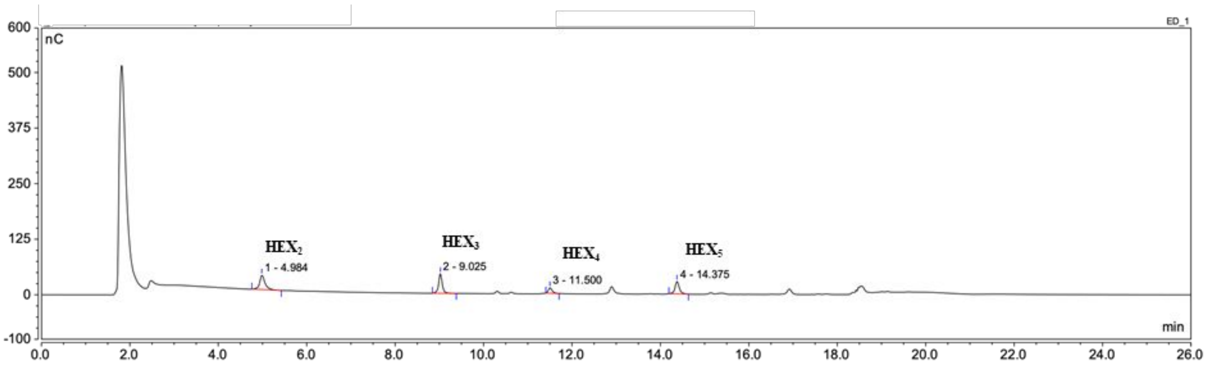

*Hv*EII variant ES2 + (1,3;1,4)-β-D-glucan

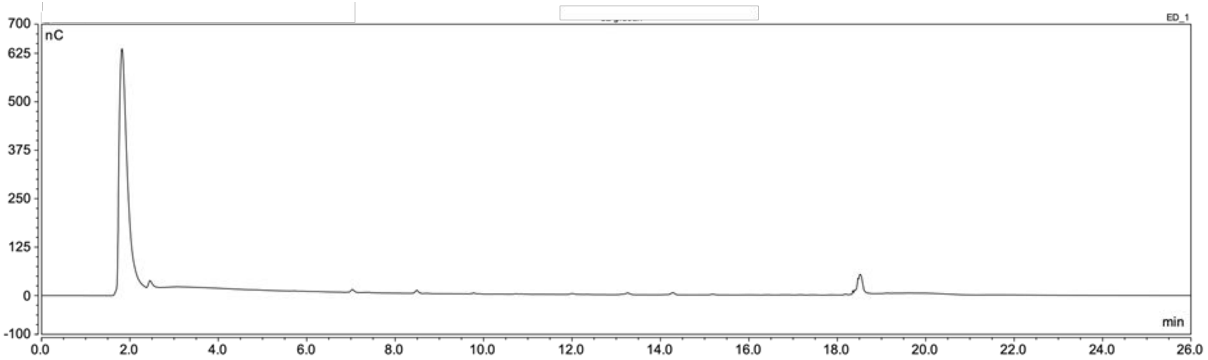

B) *Hv*EII variant ES3:

*Hv*EII variant ES3 + (1,3)-β-D-glucan

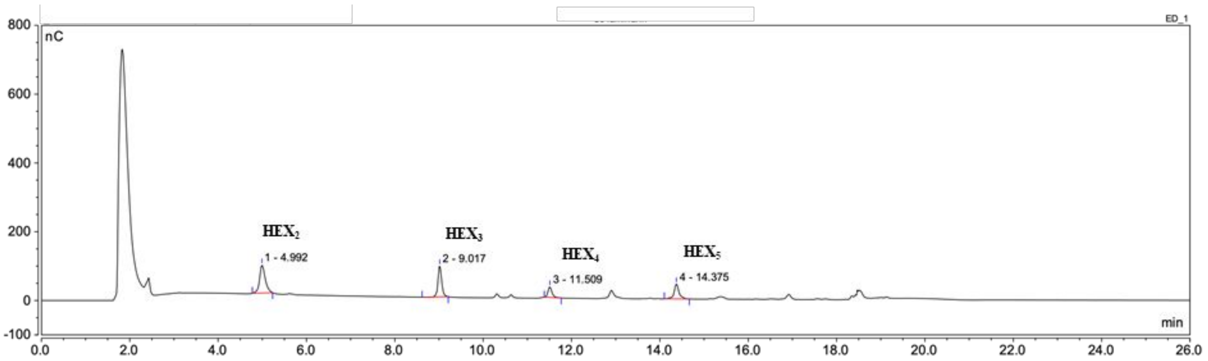

*Hv*EII variant ES3 + (1,3;1,4)-β-D-glucan

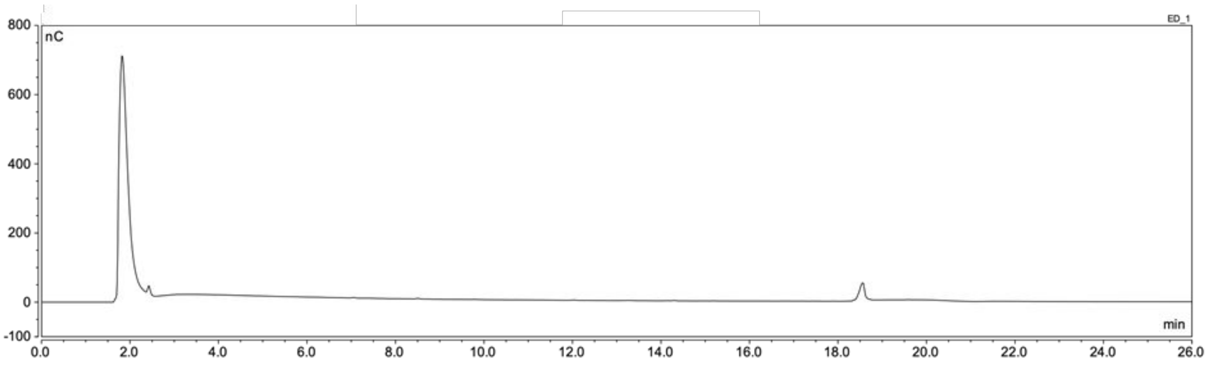

C) *Hv*EII variant ES5:

*Hv*EII variant ES5 + (1,3)-β-D-glucan

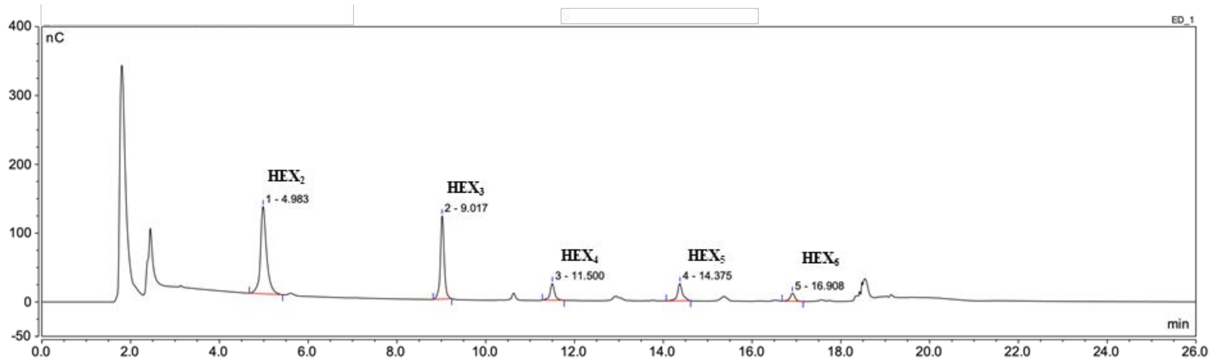

*Hv*EII variant ES5 + (1,3;1,4)-β-D-glucan

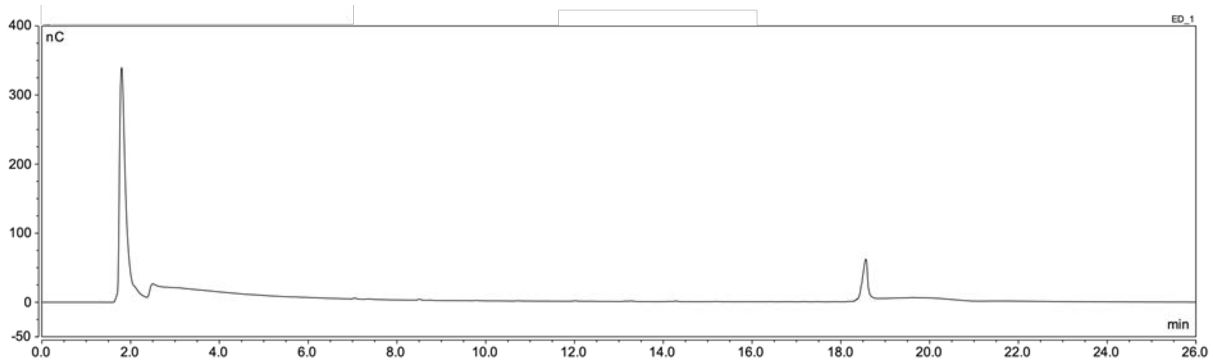

D) *Hv*GII variant GS1:

*Hv*GII variant GS1 + (1,3)-β-D-glucan

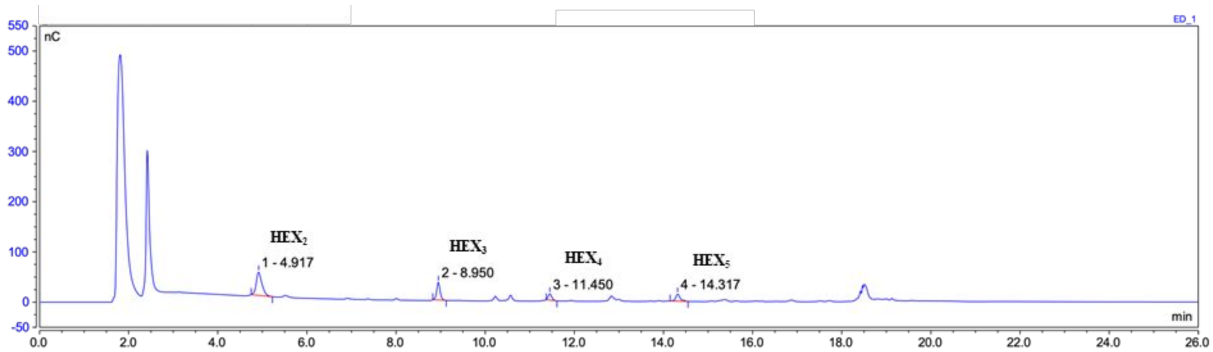

*Hv*GII variant GS1 + (1,3;1,4)-β-D-glucan

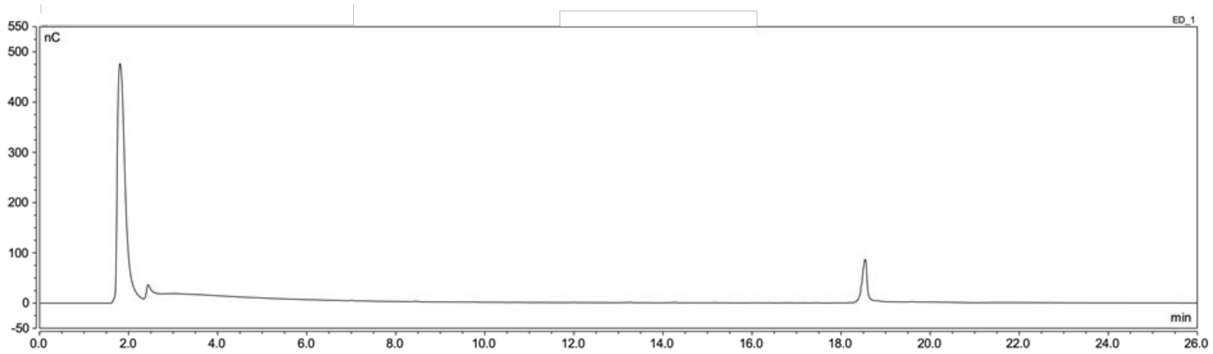

E) *Hv*GII variant GS2:

*Hv*GII variant GS2 + (1,3)-β-D-glucan

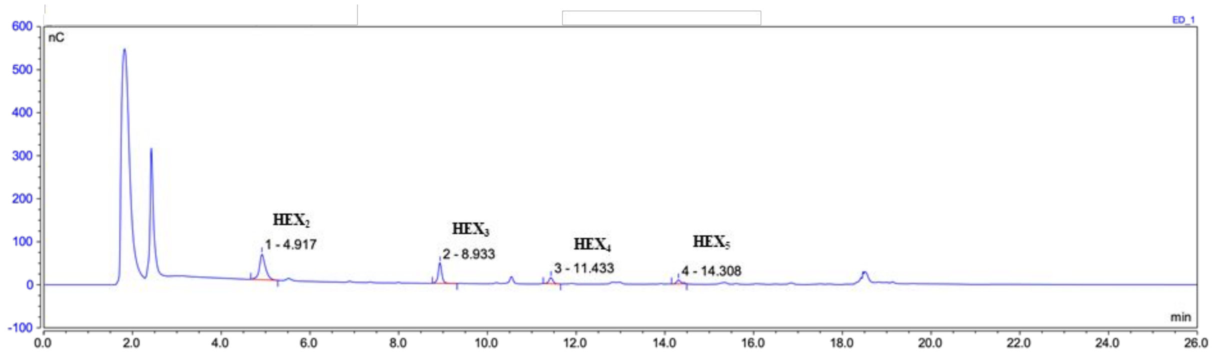

*Hv*GII variant GS2 + (1,3;1,4)-β-D-glucan

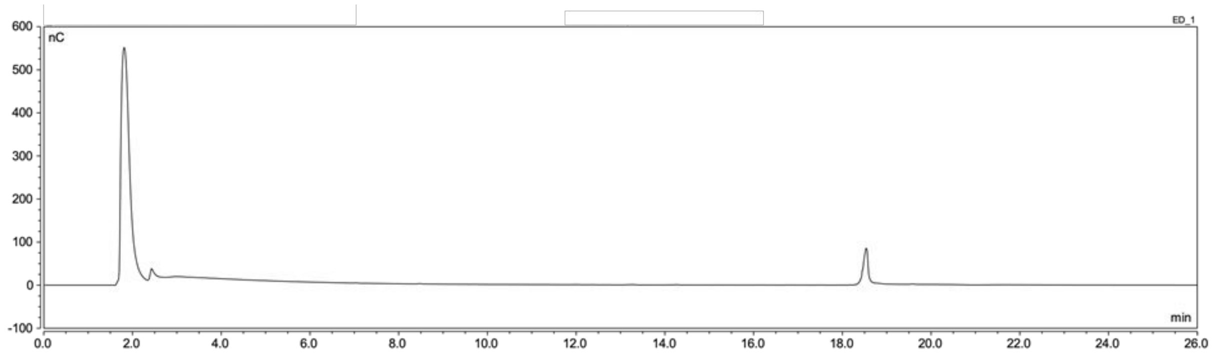

F) *Hv*GII variant GS3:

*Hv*GII variant GS3 + (1,3)-β-D-glucan

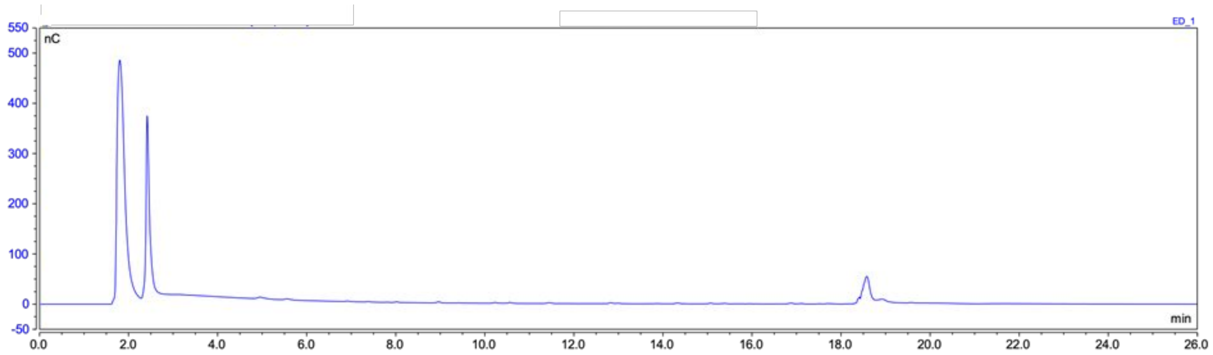

*Hv*GII variant GS3 + (1,3;1,4)-β-D-glucan

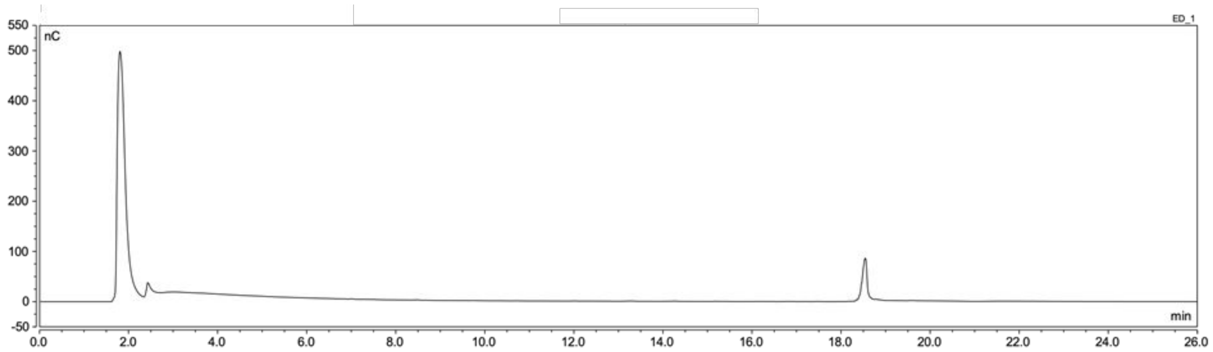

G) *Hv*GII variant GS4:

*Hv*GII variant GS4 + (1,3)-β-D-glucan

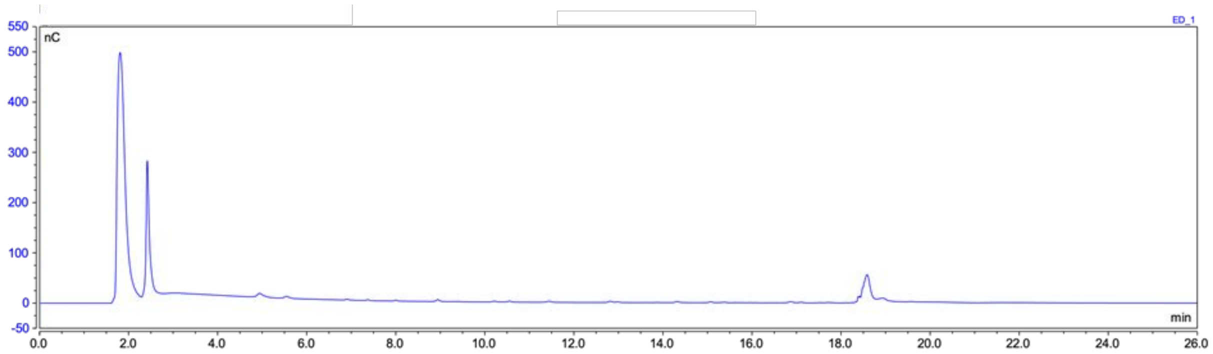

*Hv*GII variant GS4 + (1,3;1,4)-β-D-glucan

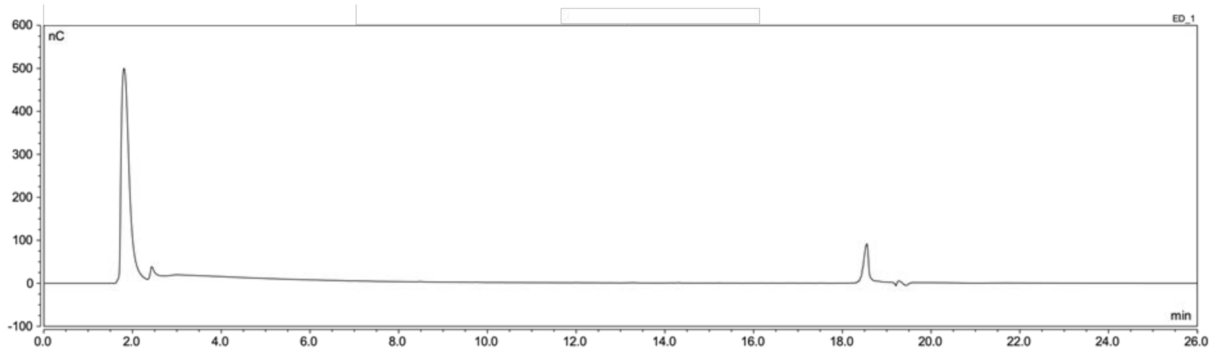

H) *Hv*GII variant GS5:

*Hv*GII variant GS5 + (1,3)-β-D-glucan

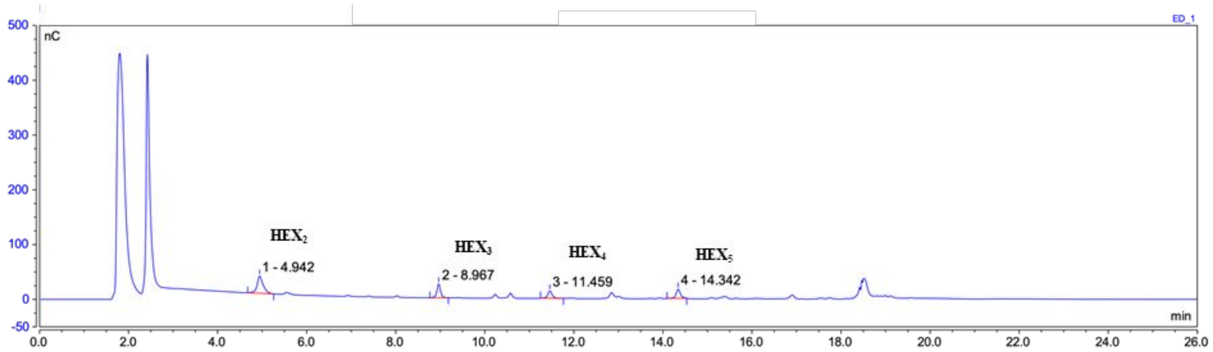

*Hv*GII variant GS5 + (1,3;1,4)-β-D-glucan

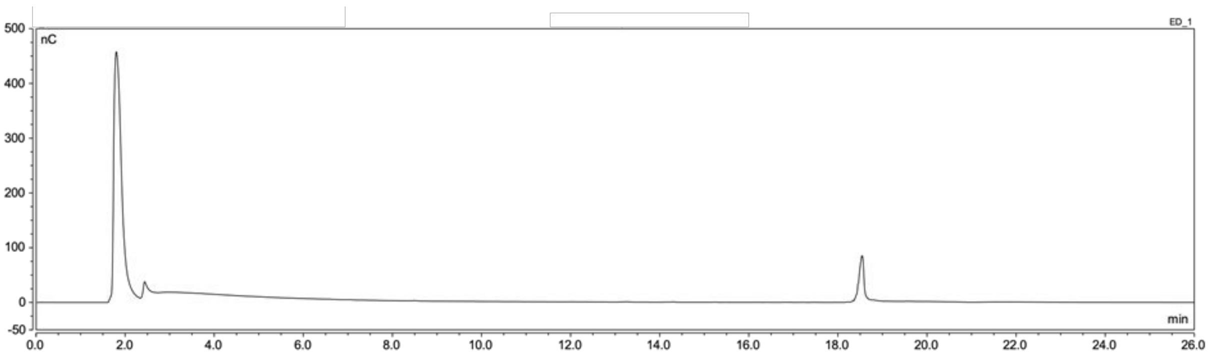

Figure S12: HPAEC analysis of hydrolysis products of (1,3)- and (1,3;1,4)-β-D-glucans treated with *Hv*EII and *Hv*GII hybrid variants. A) *Hv*EII varian ES2, B) *Hv*EII variant ES3, C) *Hv*EII variant ES5, D) *Hv*GII variant GS1, E) *Hv*GII variant GS2, F) *Hv*GII variant GS3, G) *Hv*GII variant GS4 and H) *Hv*GII variant GS5. Peaks marked Hex_2, Hex_3, Hex_4 Hex_5 and Hex_6 correspond to laminaribiose, laminaritriose, laminaritetraose, laminaripentaose and laminarihexaose, respectively from the (1,3)-β-D-glucan.

Supplementary figure S13: MALDI-TOF analysis of hydrolysis products of laminarin (1,3-β-D-glucan) digested by *Hv*EII variant V6

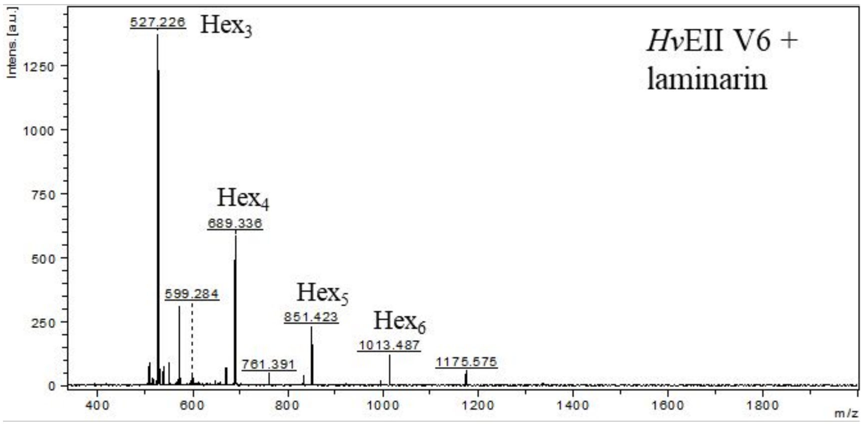

Figure S13: Hydrolysis products of *Hv*EII variant V6 analysed by MALDI-TOF MS. Molecular ions of $[M + Na]^+$ corresponding to laminaritriose, laminaritetraose, laminaripentaose and laminarihexaose are labelled as in Hex_3, Hex_4, Hex_5, and Hex_6, respectively.

Supplementary figure S14: MALDI-TOF analysis of hydrolysis products of laminarin (1,3-β-D-glucan) digested by *Hv*EII hybrid variant ES4

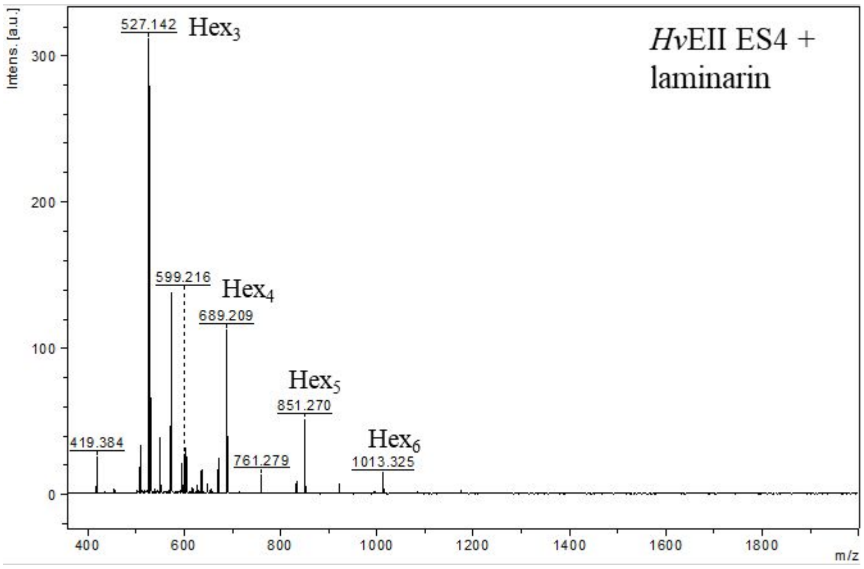

Figure S14: Hydrolysis products of *Hv*EII hybrid variant ES4 analysed by MALDI-TOF MS. Molecular ions of $[M + Na]^{+}$ corresponding to laminaritriose, laminaritetraose, laminaripentaose and laminarihexaose are labelled as in Hex_3, Hex_4, Hex_5, and Hex_6, respectively.

Supplementary figure S15: MALDI-TOF analysis of hydrolysis products of laminarin (1,3-β-D-glucan) digested by *Hv*EII hybrid variant ES5

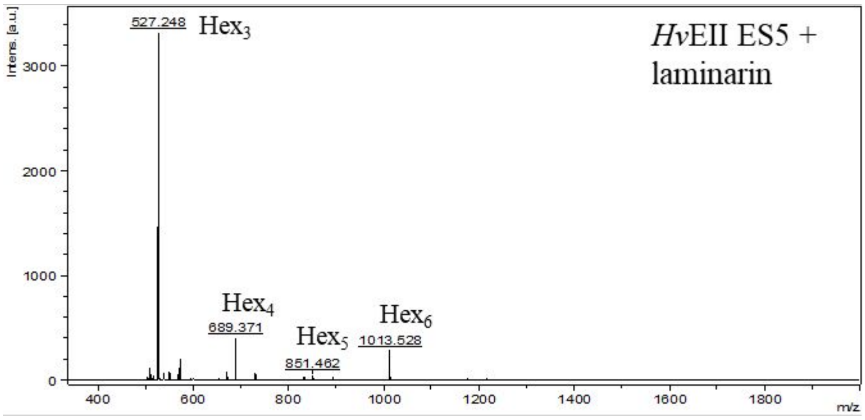

Figure S15: Hydrolysis products of *Hv*EII hybrid variant ES5 analysed by MALDI-TOF MS. Molecular ions of $[M + Na]^+$ corresponding to laminaritriose, laminaritetraose, laminaripentaose and laminarihexaose are labelled as in Hex_3, Hex_4, Hex_5, and Hex_6, respectively.

Table S1: Accession numbers of (1,3;1,4)-β-D-glucanases and (1,3)-β-D-glucanases in phylogenetic trees

Accession number	Name
CAB68132.1	*Arabidopsis thaliana* (1,3)-β-D-glucanase
EAA60742.1	*Aspergillus nidulans* FGSC A4 (1,3)-β-D-glucanase
AAP33176.1	*Avena sativa* (1,3)-β-D-glucanase
CAA53545.1	*Beta vulgaris subsp. vulgaris* (1,3)-β-D-glucanase
CAA09765.1	*Cichorium intybus* (1,3)-β-D-glucanase
CAA03908.1	*Citrus sinensis* (1,3)-β-D-glucanase
AAA33946.1	*Glycine max* (1,3)-β-D-glucanase
P34742	*Hordeum vulgare* (1,3)-β-D-glucanase isoenzyme GI
P15737.1	*Hordeum vulgare* (1,3)-β-D-glucanase isoenzyme GII
Q02126.1	*Hordeum vulgare* (1,3)-β-D-glucanase isoenzyme GIII
Q02437	*Hordeum vulgare* (1,3)-β-D-glucanase isoenzyme GIV
Q02438	*Hordeum vulgare* (1,3)-β-D-glucanase isoenzyme GV
BAJ93582.1	*Hordeum vulgare* (1,3)-β-D-glucanase isoenzyme GVII
AAB41551.1	*Medicago sativa* (1,3)-β-D-glucanase
AAD33880.1	*Nicotiana tabacum* (1,3)-β-D-glucanase
AAK58515.1	*Olea europaea* (1,3)-β-D-glucanase
AAT01345.1	*Oryza sativa Japonica* Group (1,3)-β-D-glucanase
AAA33648.1	*Pisum sativum* (1,3)-β-D-glucanase
AAA34648.1	*Saccharomyces cerevisiae* (1,3)-β-D-glucanase
AGQ89281.1	*Saccharum hybrid cultivar* (1,3)-β-D-glucanase
CAE52322.1	*Solanum tuberosum* (1,3)-β-D-glucanase
EES02042.1	*Sorghum bicolor* (1,3)-β-D-glucanase
CAA77085.1	*Triticum aestivum* (1,3)-β-D-glucanase
PWZ29984.1	glucan *endo*-(1,3)-β-glucanase GII *Zea mays*
XP_034596520.1	glucan *endo*-(1,3)-β-glucanase GII-like *Setaria viridis*
XP_004971233.1	glucan *endo*-(1,3)-β-glucanase GII *Setaria italic*
ADM86867.1	β-(1,3)-glucanase *Oryza sativa japonica*
XP_037408323.1	glucan *endo*-(1,3)-β-glucanase GII-like *Triticum dicoccoides*
XP_020198692.1	glucan *endo*-(1,3)-β-glucanase GII-like *Aegilops tauschii*
XP_024313961.1	glucan *endo*-(1,3)-β-glucanase GII *Brachypodium distachyo*
XP_002459077.1	glucan *endo*-(1,3)-β-glucanase GII *Sorghum bicolor*
3DGT (PDB Database)	*endo*-(1,3)-β-glucanase *Streptomyces sioyaensis*
Q02345	*Hordeum vulgare* (1,3; 1,4)-β-D-glucanase isoenzyme EI
P12257.1	*Hordeum vulgare* (1,3; 1,4)-β-D-glucanase isoenzyme EII
XP_037463194.1	Lichenase-2-like *Triticum dicoccoides*
XP_020194728.1	Lichenase-2 *Aegilops tauschii*
XP_003568572.1	Lichenase-2 *Brachypodium distachyon*
XP_034584561.1	Lichenase-2-like *Setaria viridis*
XP_004962259.1	Lichenase-2 *Setaria italic*
PWZ18571.1	Lichenase-2 *Zea mays*
XP_021303353.1	Lichenase-2-like *Sorghum bicolor*
XP_015639379.1	Lichenase-2 *Oryza sativa japonica*
